# Additive Manufacturing Post-Processing Treatments, a Review with Emphasis on Mechanical Characteristics

**DOI:** 10.3390/ma16134610

**Published:** 2023-06-26

**Authors:** Alin Diniță, Adrian Neacșa, Alexandra Ileana Portoacă, Maria Tănase, Costin Nicolae Ilinca, Ibrahim Naim Ramadan

**Affiliations:** Mechanical Engineering Department, Petroleum-Gas University of Ploiești, 100680 Ploiesti, Romania; adinita@upg-ploiesti.ro (A.D.); adnea@upg-ploiesti.ro (A.N.); maria.tanase@upg-ploiesti.ro (M.T.); costinilinca@yahoo.com (C.N.I.); ing_ramadan@yahoo.com (I.N.R.)

**Keywords:** additive technology, 3D printing, heat treatment, polymers, high-entropy alloys

## Abstract

Additive manufacturing (AM) comes in various types of technologies and comparing it with traditional fabrication methods provides the possibility of producing complex geometric parts directly from Computer-Aided Designs (CAD). Despite answering challenges such as poor workability and the need for tooling, the anisotropy of AM constructions is the most serious issue encountered by their application in industry. In order to enhance the microstructure and functional behavior of additively fabricated samples, post-processing treatments have gained extensive attention. The aim of this research is to provide critical, comprehensive, and objective methods, parameters and results’ synthesis for post-processing treatments applied to AM builds obtained by 3D printing technologies. Different conditions for post-processing treatments adapted to AM processes were explored in this review, and demonstrated efficiency and quality enhancement of parts. Therefore, the collected results show that mechanical characteristics (stress state, bending stress, impact strength, hardness, fatigue) have undergone significant improvements for 3D composite polymers, copper-enhanced and aluminum-enhanced polymers, shape memory alloys, high-entropy alloys, and stainless steels. However, for obtaining a better mechanical performance, the research papers analyzed revealed the crucial role of related physical characteristics: crystallinity, viscosity, processability, dynamic stability, reactivity, heat deflection temperature, and microstructural structure.

## 1. Introduction

The technology of 3D printing has rapidly gained popularity over the past few years due to its versatility and ability to produce complex parts and structures. Applications of 3D printing include the manufacturing of various products such as medical devices, aerospace components, and consumer goods. Despite its benefits, there are certain limitations associated with 3D printing that include poor surface quality, dimensional inaccuracies, and low mechanical strength. These limitations highlight the need for post-processing treatments such as sanding, polishing, and heat treatment to enhance the properties and functionality of the 3D-printed parts. 

**Figure 1:**
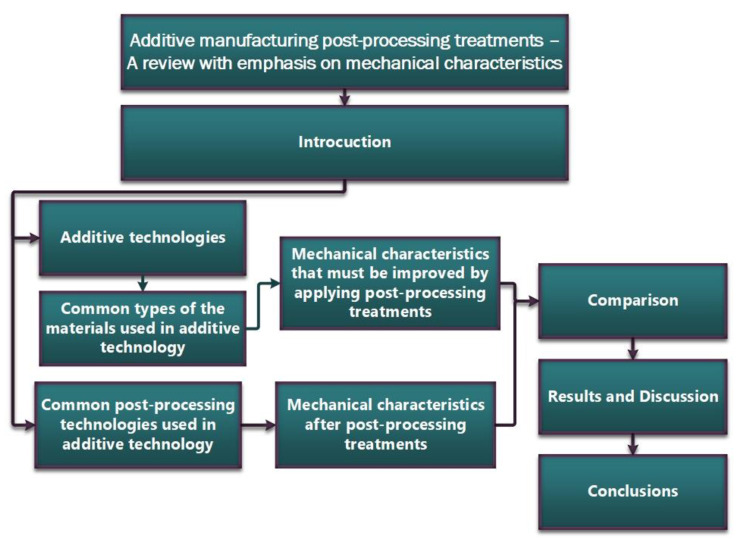
Graphical abstract (Source: authors as the basis of article content).

As can be seen in the graphical abstract (Figure 1), this review article aims to highlight the applications of post-processing of 3D-printed parts and their associated limitations, as well as the importance of these treatments to overcome these limitations and improve the quality and performance of the 3D-printed parts.

Additive printing technology and its applications are vast, ranging from healthcare to aerospace. Some of the most significant applications of 3D printing technology are:

- Healthcare

Prosthetics, implants, and surgical instruments. In some cases, 3D printing has been used to produce patient-specific models for surgical planning, allowing for more accurate procedures.

- Aerospace

Light and complex parts for aircraft, such as engine components and wing structures. This technology has allowed for faster and more efficient production processes.

- Manufacturing

Prototyping and production of small batches of parts, such as in the automotive and electronics industries.

- Architecture and construction

Production of small-scale models of buildings, allowing architects and engineers to visualize designs and make modifications before construction begins.

- Education

Used in education to teach students about engineering, design, and manufacturing principles.

The 3D printing materials refer to the substances used in the creation of 3D-printed objects. They come in a variety of forms, including filaments, resins, and powders, and can be made from a range of materials, including plastic, metal, and ceramic.

Some popular 3D printing materials for the filaments include:

- PLA (Polylactic Acid)

Biodegradable and eco-friendly plastic commonly used for its ease of printing and low warping tendency. It is ideal for creating prototypes, toys, and figurines.

- ABS (Acrylonitrile Butadiene Styrene)

Strong, durable, and lightweight plastic that is often used in the creation of products such as phone cases and automotive parts.

- PETG (Glycol-Modified Polyethylene Terephthalate)

Flexible, food-safe plastic that is easy to print and resistant to impacts, chemicals, and UV light.

Resins are another popular 3D printing material. They are typically used with SLA (Stereolithography) and DLP (Digital Light Processing) printers and offer higher precision and accuracy than filaments. Some common resins include:

- UV resin

Fast-curing, high-resolution resin that is ideal for creating intricate and detailed models.

- Flexible resin

Bendable and soft resin that is suitable for creating flexible and soft-touch parts, such as phone cases.

- Castable resin

This resin can be melted and cast in metal to produce high-quality jewelry and other metal objects.

In addition, there are 3D printing powders, which include materials such as metal and ceramic. These materials are typically used with SLS (Selective Laser Sintering) printers and are best suited for creating complex and robust objects, such as aerospace parts and medical implants.

Popular 3D-printing metallic filament materials include:

- Titanium

This is one of the most commonly used metals for 3D printing. It has excellent strength, light weight, and high corrosion resistance.

- Stainless steel

Known for its durability, corrosion resistance, and high strength-to-weight ratio. Widely used in aeronautical and medical applications.

- Aluminum

Aluminum is light, strong, and has good thermal and electrical conductivity. It is commonly used in the aerospace, automotive, and consumer electronics industries.

- Inconel

Inconel is renowned for its high strength, excellent resistance to corrosion and oxidation, and high temperature resistance. The aerospace and power industries use it extensively.

- Cobalt Chromium

This metal is known for its excellent biocompatibility, high strength, and corrosion resistance and is used in the medical and dental industries.

- Copper

Known for its high thermal and electrical conductivity, this metal is commonly used in the electronics industry.

- Gold

Widely used in jewelry, this metal is highly prized for its unique aesthetic qualities.

- Nickel

It is known for having high strength, resisting corrosion, and withstanding high temperatures. It is widely used in the aerospace and energy industries.

- Bronze

Renowned for its durability, corrosion resistance, and unique appearance. It is often the material of choice for art and sculpture.

- Silver

Valued for its unique aesthetic qualities, this metal is often used in jewelry and decorative objects.

There are a wide range of 3D printing materials available, each with its own unique properties and capabilities. When choosing a material, it is important to consider the desired end-product, the printing method, and the desired level of precision and accuracy.

As stated in the previous paragraphs, the additive printing technology has revolutionized many industries, it is still in its early stages of development, and limitations such as surface finish, strength, accuracy, and cost mean that post-processing treatments are often necessary.

Heat treatment is a process commonly applied to 3D-printed parts to improve their mechanical properties. The process involves exposing the parts to high temperatures to cause a change in the microstructure of the material, resulting in a desired change in properties such as increased strength, improved dimensional stability, and reduced brittleness.

However, despite the many benefits of 3D printing, there are also limitations that prove the need for post-processing treatments. Some of the limitations include:

- Surface finish

The printed parts often have a rough and porous surface that requires post-processing treatments such as sanding, smoothing, and painting.

- Strength and durability

The 3D-printed parts are often weaker than conventionally manufactured parts and require additional post-processing treatments such as reinforcement and hardening.

- Accuracy

The additive printing technology is still developing, and the accuracy of printed parts can vary depending on the technology used. Post-processing treatments such as finishing and calibration are often necessary to achieve the desired level of accuracy.

- Costs

The technology can be expensive, and post-processing treatments can add to the overall cost of the process.

There are several types of heat treatment commonly applied to 3D-printed parts, including annealing, normalizing, hardening, and tempering. Annealing is a process of heating and cooling the parts to reduce internal stress and improve their toughness and ductility. Normalizing involves heating the parts to a temperature above the critical range and cooling them in still air to achieve a uniform grain structure. Hardening is a process of heating and rapidly cooling the parts to form a hard, strong, and brittle structure. Tempering is a process of reheating the hardened parts to a lower temperature to reduce their brittleness and improve toughness.

The choice of heat treatment depends on the material and the desired properties of the parts. For example, Acrylonitrile Butadiene Styrene (ABS) parts are typically annealed, while stainless steel parts are hardened and tempered. The process parameters, including the temperature, cooling rate, and the time at temperature, are critical to achieving the desired properties and must be carefully controlled.

The heat treatment is an important post-processing step in the manufacture of 3D-printed parts. It can significantly improve the mechanical properties of the parts, making them more suitable for a wide range of applications. However, it requires a careful control of the process parameters to achieve the desired results and must be tailored to the specific material and application requirements.

The parameters for heat treatment of 3D-printed parts include:

- Heat treatment temperature

This is a crucial factor for heat treatment, as it determines the microstructural changes that occur in the material of the 3D parts. The temperature must be carefully controlled to ensure that the desired properties are achieved.

- Duration of heat treatment

The time taken to heat treat the 3D parts is also important. The longer the 3D part is held at the desired temperature, the more time it has to reach equilibrium, and the greater the chances of the desired microstructural changes occurring.

- Atmosphere in the heat treatment furnace

The atmosphere surrounding the 3D part during heat treatment is also important. Controlled atmospheres can be used to prevent unwanted reactions during the heat treatment process.

- Cooling rate

The rate at which the 3D part is cooled after heat treatment also affects its properties.

- Equipment (furnace for heat treatment)

The furnace used for heat treatment can also affect the outcome. For example, the type of furnace used can impact the temperature uniformity, heating rate, and cooling rate of the 3D-printed parts.

- Post-processing operations

Any post-processing, such as machining, sanding, or polishing, should be performed after heat treatment to avoid affecting the desired properties.

The parameters for heat treatment of 3D-printed parts are crucial for achieving the desired properties in the final product. Careful consideration and control of temperature, time, atmosphere, cooling rate, equipment, and post-processing is essential for successful heat treatment.

**Figure 2:**
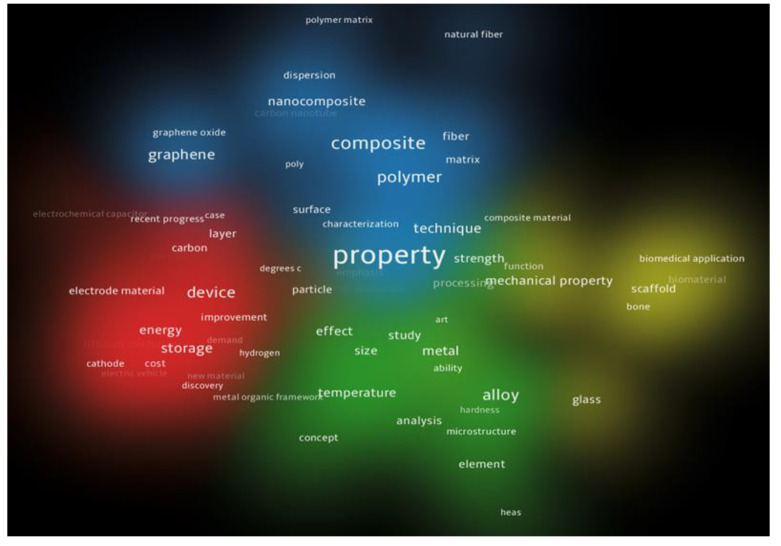
Common words in scientific publications (Source: authors based on articles analyzed).

In order to analyze the most relevant concepts in the field of this review, a bibliometric analysis was used, using the academic platform Web of Science as the source of scientific articles analyzing 3D printing materials and post-processing treatments. Therefore, the content of 100 highly cited articles related to these topics on Web of Science were explored to highlight the structure of the scientific field, using content analysis that examined the most common words (3D printing technologies, materials, post-processing treatments, mechanical properties) and the relationship between these words. The empirical analysis showed that the most common words in the full content of the selected articles, apart from the keywords used, are presented in Figure 2, including “mechanical property” and “structure”.

**Figure 3:**
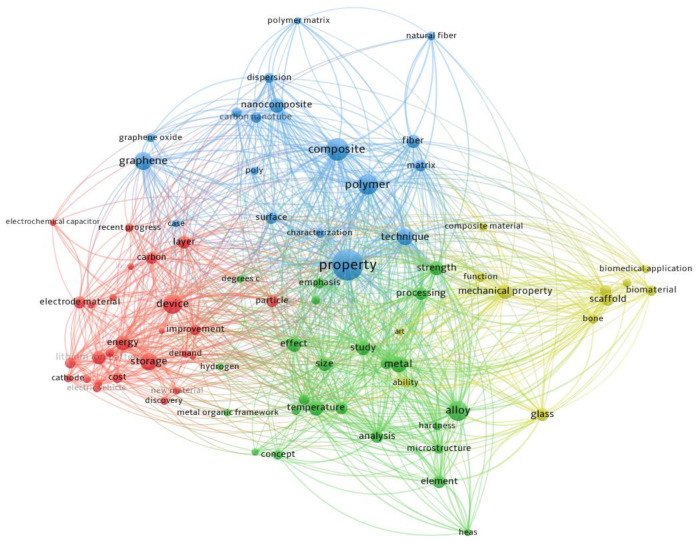
Word network in renewable energy transition scientific publications’ content (Source: authors based on articles analyzed).

From the analysis of the clusters shown in Figure 2 and Figure 3, the nature of the materials and the different technologies used to make parts by additive technologies for various industries are closely related to the size of the mechanical characteristics needed to meet the demands of practical applications.

From the above, it can be seen that additive technology, also known as additive manufacturing, is a manufacturing process that uses a 3D model created with CAD technology to build a three-dimensional object from a variety of materials by depositing successive thin layers of material. A literature review of over 100 key papers in the field showed that this technology has revolutionized the way parts and components are produced in various industries, allowing the creation of more complex and precise parts than using traditional technologies. However, parts produced using additive technology can be susceptible to both internal stress parity and other technological problems that can affect their strength and durability. Therefore, heat treatments are often required to improve the properties of additive parts. These heat treatments can include various types of heat treatments and related technological operations. The following are the main advantages and disadvantages of using heat treatments for additive manufacturing parts.

It is also clear from the literature review that heat treatment of additive manufactured parts can provide a number of benefits, which are listed below:

- Improved mechanical properties of additive parts, including hardness and strength. Additive parts can have internal stresses and micro-cracks that can affect their strength and durability. Heat treatments can reduce these stresses and harden the material, improving part strength and durability;
- Elimination of internal stresses. The 3D printing process can create internal stresses in parts, and heat treatments can help eliminate these stresses, reducing the risk of parts breaking or cracking. The process can also reduce thermal distortion and shrinkage that occurs during the manufacturing process, improving the dimensional stability of parts;
- Improved geometric accuracy. Heat treatment can help reduce non-uniform deformation and shrinkage during the production process, which can improve the geometric accuracy of parts;
- Uniformity/homogeneity of part structure. The 3D printing process can produce parts with a porous structure, and heat treatments can help strengthen the structure by eliminating porosity and voids, thus increasing the homogeneity of the materials used in 3D printing, which can lead to better quality printed parts;
- Impurity removal. The 3D printing process can introduce impurities into the material used, and heat treatments can help remove these impurities;
- Improved adhesion between layers. Heat treatments can increase adhesion between layers of material, which can lead to better stability and strength of printed parts;
- Reduced brittleness. Some materials used in 3D printing can be brittle, and heat treatments can help reduce brittleness by improving hardness and strength;
- Improved ductility. Heat treatments can increase the ductility of some materials, resulting in a better ability to deform under stress;
- Increased corrosion resistance. Parts produced by additive technology can be more susceptible to corrosion than parts produced by other methods. Heat treatments can increase the corrosion resistance of materials, which can extend the life of printed parts;
- Improved surface finish. Heat treatments can also provide the appropriate surface roughness required for certain technical applications, improving the quality and appearance of parts;
- Improved ductility. Parts produced by additive technology can be more brittle than parts produced by other methods. Heat treatments can improve the ductility of the material, making it less susceptible to cracking or fracture;
- Increased thermal and electrical conductivity. Parts produced by additive technology may have lower thermal and/or electrical conductivity than parts produced by other methods. Heat treatments can increase the thermal and/or electrical conductivity of parts, improving their ability to transfer heat;

The following are some of the categories of reasons why problems/disadvantages may arise in the use of post-processing heat treatments:

- Part distortion. If inappropriate temperature regimes are used, they can lead to deformation of additive manufactured parts;
- Undesirable changes in mechanical properties. Additive technology is a manufacturing process that involves the variation of many specific factors that give parts certain mechanical properties. Heat treatments can affect these properties, often in unexpected ways, which can lead to reduced performance;
- Increased porosity. Post-treatment heat treatments can lead to increased porosity in additive manufactured parts, which can adversely affect both the strength and durability of the parts;
- Irregularities on part surfaces. Heat treatments applied to parts produced by additive technology can cause irregularities on the surface of the parts, which can affect their accuracy and quality;
- Increased production time. The length of heat-treatment cycles often involves additional heating, holding, and cooling time, which can significantly increase the production time of parts;
- Higher production costs. Due to the high consumption of equipment/tools, energy, consumables, and labor, heat treatment involves additional costs that can significantly increase the production cost of parts;
- Creation of critical situations in the production process. Heat treatments can be difficult to apply during the part production process, which can lead to complications and delays;
- Increased risk of damage. Both high temperatures and sudden temperature changes can cause damage to parts during heat treatment, resulting in rejection;
- Dimensional limitations. Due to the volume of the furnaces, part dimensions can be limited to small values, which can restrict design and manufacturing options;
- Inherent risks of failure in service. If not carried out correctly, heat treatments can cause parts to fail in service, which can be dangerous in critical applications;

In the next subsections, the materials used and the method of analysis used to carry out this review are presented in relation to the use of heat treatments on parts produced by technological processes specific to additive technologies.

## 2. Studied Additive Technologies

**Figure 4:**
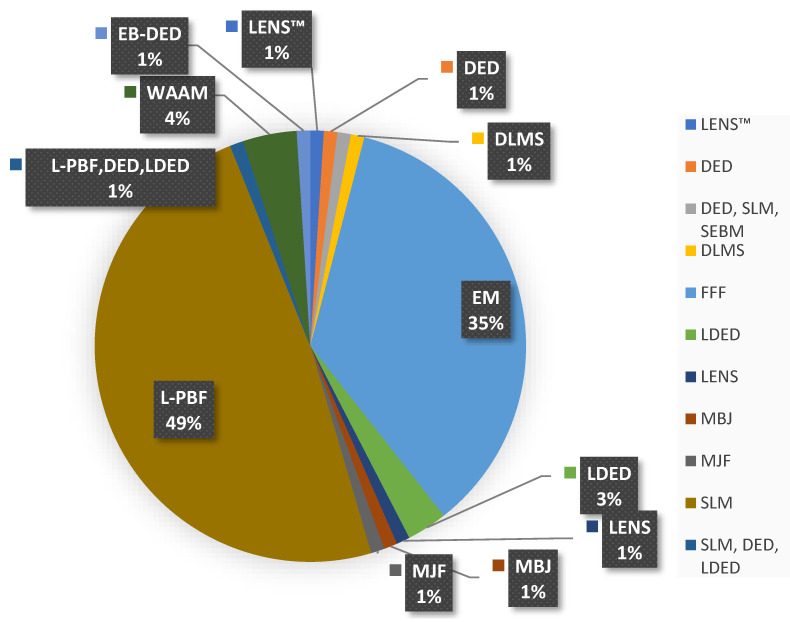
Distribution of studied additive technologies.

The present study conducted a comprehensive analysis of several additive technologies and their respective variations. It is known that the diverse post-treatment procedures employed are inherently specific to the particular type of additive manufacturing process utilized. It is observable from the graphical data presented in Figure 4 that a substantial proportion of the post-treatment techniques employed in the analyzed literature are utilized for parts fabricated via Laser Beam Bed Fusion (L-PBF) and represents a significant percentage of 49% of the post-treatment techniques applied; in addition, parts generated through the technology material extrusion (ME) comprise 35% of the total. The remaining post-treatment methodologies are distributed across a range of additive manufacturing processes, including Laser Engineered Net Shaping™ (LENS™), Directed Energy Deposition (DED), Selective Electron Beam Melting (SEBM), Digital Light Processing Stereolithography (DLMS), Laser Direct Energy Deposition (LDED), Laser Powder Bed Fusion (L-PBF), Material Jetting (MBJ), Multi Jet Fusion (MJF), and Wire Arc Additive Manufacturing (WAAM), among others, in varying proportions, that are shown in Figure 4.

### 2.1. Laser Powder Bed Fusion (L-PBF)

**Figure 5:**
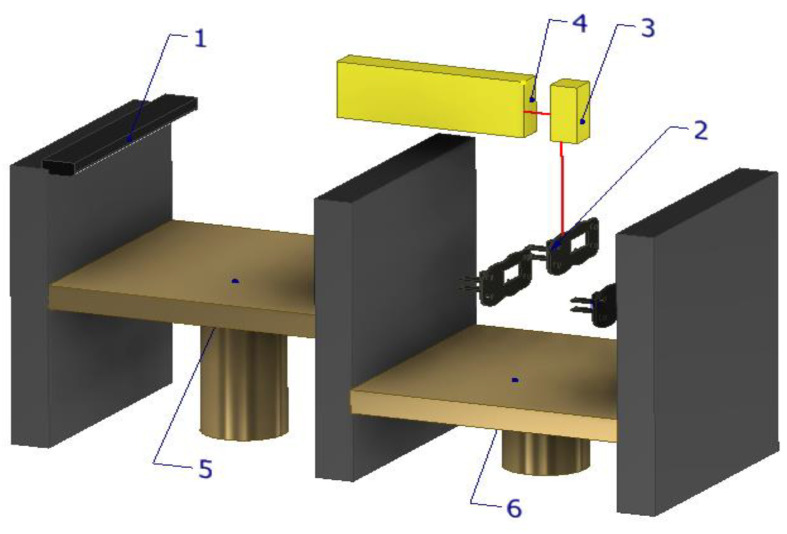
Principal graphical scheme of L-PBF.

Laser Powder Bed Fusion (L-PBF L-PBF [1] represents the most extensively utilized powder bed-based additive manufacturing (AM) technique. The L-PBF process employs a laser beam for melting and fusing metal powders together, with a thin layer of powder uniformly distributed over the substrate or a previously deposited layer (Figure 5). The laser beam selectively melts and fuses the powder particles in accordance with the computer-aided design (CAD) model. In order to fabricate parts with optimized microstructure and properties that are free of defects, the L-PBF technique necessitates the careful adjustment of several process parameters, including laser power, laser scanning speed, layer thickness, hatching distance, and scanning strategy. Typically, depending on the reactivity of the metallic powders employed, the L-PBF process is performed in a closed chamber that is filled with an inert atmosphere, such as argon or nitrogen. The principal motivation for applying post-treatments to L-PBF parts are presented as follows and the primary factors behind the implementation of post-processing techniques on L-PBF parts lies in the pursuit of enhanced material properties and improved functional performance of the fabricated components:

- Residual stress [2,3];
- Microstructure high porosity [4,5];
- Microstructure instability [5,6,7,8];
- Homogenization, microstructural refinement, and martensite-to-austenite reversion [9];
- Parts distortion [6];
- Heterogeneous microstructure [10];
- Coarse plate martensite with uneven distribution [11];
- Anisotropic microstructures [12];
- Low material density [13];
- Martensite matrix with trace amount of austenite phase [14,15];
- Enhance superelasticity of NiTi structures [16,17,18];
- Stress-induced cracks and residual porosity [19];
- Intermetallic precipitates [20];
- Internal defects, such as entrapped-gas-pores or lack-of-fusion [21];
- High nitrogen content [22];
- Porosity, grain morphology and precipitates [23,24].

The principal scheme (Figure 5) of L-PBF involves several key components, including a build platform, a powder bed, a laser system, and a computer control system. The process begins with a thin layer of metal powder spread uniformly across the build platform. The material used in powder form is leveled using the leveling cylinder (1). The raw material powder acts as a support for the printed parts (2). A cross section of the part is melted by the laser, solidifying afterwards. The laser beam is focused by a laser generator system (4) and passes through a scanning mirror (3). A new layer of powder from the powder reservoir (5) is reapplied and then, the construction platform (6) lowers with a layer thickness. The process is repeated until a solid part is printed. The only difference between SLS and L-PBF is that L-PBF relies on the complete melting of metal powder particles. Laser beam bed fusion technology can be applied to any metal. Overall, the principal scheme of L-PBF involves the precise control of laser melting and powder deposition to create a solid metal part with high accuracy and dimensional integrity. The technique has numerous applications in industries such as aerospace, automotive, medical, and tooling.

### 2.2. Material Extrusion (ME)

This is a type of 3D printing technology (named in this study according to the ISO/ASTM 52900:2015(E) standard) that uses a thermoplastic filament as the printing material. The process involves feeding a spool of the filament through a heated extruder, which melts the material and extrudes it through a small nozzle onto a build platform. The nozzle moves back and forth in the X and Y directions, while the build platform moves down in the Z direction, building up the object layer by layer.

ME is popular because it is a relatively low-cost and accessible 3D printing technology that can be used for a wide range of applications, including prototyping, tooling, and production parts. There is also a large variety of filaments available, including standard plastics such as PLA and ABS, as well as more advanced materials such as carbon fiber, nylon, and even metal-filled filaments.

However, ME parts can sometimes exhibit certain limitations, such as poor surface finish, low strength, and dimensional inaccuracies. Post-treatments can be applied to ME parts to address these limitations and enhance the overall performance and quality of the printed parts. Synthesized from relevant literature, various factors can drive the motivation for the application of post-treatments on ME such as:

- Stress relief, dimensional instability [25,26,27];
- Need for controllable crystallinity and deterioration phenomenon of polymer materials [28];
- Enhancing tensile properties [29,30,31,32,33,33,34,35];
- Improving composite flexural properties [36];
- Poor interfacial adhesion between the printed layers [37,38];
- Enhancing interlayer strength [39,40,41];
- Improving thermal conductivity [42,43];
- Increasing crystallinity [44,45,46,47,48,49,50];
- Increasing fracture toughness [51];
- Poor bead-to-bead interfacial bonding and relatively high void content [52,53];
- Reducing anisotropy [54];
- Behavior study of annealed defect parts [55];
- Eliminating the warpage of semi-crystalline polymer [56];
- Increasing the dynamic flexural properties [57];
- Poor interfacial bond formation [58].

**Figure 6:**
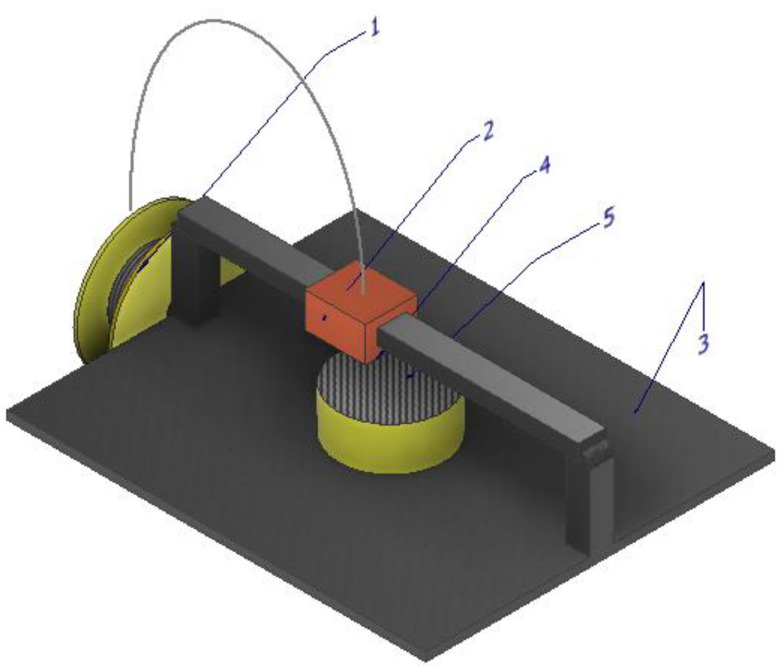
Principal graphical scheme of ME.

Components can be manufactured via the utilization of threads of solid state thermoplastic material. The process (Figure 6) entails winding the wire onto a filament roll (1) and feeding it into an extruder head (2), the initial layer of the part adheres to the build platform (3), and for parts containing console components, support structures made of either the same material or specially formulated materials that dissolve in water are required. The filament is subsequently propelled through a heated nozzle (4), where it is liquefied. The 3D printer facilitates the movement of the nozzle, which deposits the molten material in precise locations forming the part (5), following the predetermined path prescribed by the G-code.

### 2.3. Laser Engineered Net Shaping (LENS)

Laser powder forming, known as laser engineered net shaping, is a state-of-the-art additive manufacturing technology utilized to produce metal parts directly from a computer-aided design (CAD) solid model. This innovative technique involves injecting metal powder into a molten pool formed by a concentrated, high-powered laser beam. Several proprietary techniques, such as direct metal deposition (DMD) and laser consolidation (LC), are similar to this approach. Unlike powder bed-based techniques such as laser beam bed fusion (L-PBF), laser powder forming enables the fabrication of significant-sized objects, sometimes surpassing several feet in length. Utilizing the trademarked Laser Engineered Net Shaping (LENS™) technique developed by Optomec Inc. in Albuquerque, NM, cylindrical equiatomic NiTi alloy samples were fabricated with diameters of 12 mm and lengths of 40 mm. The process involved using a Ytterbium-doped fiber laser with a 0.5 mm beam diameter to melt and deposit pre-alloyed NiTi powder with a nominal composition of 55.2 wt.% Ni and balance Ti, featuring trace amounts of O, N, and C with an average particle size between 50 and 150 mm. The powder feed rate was regulated at 4.36 g/min. Two sets of samples were produced using different laser power inputs (200 W and 400 W) and laser scan rates (10 mm/s and 20 mm/s), with each set’s laser energy density (E) calculated using the formula E = Pvd, where P is the laser power, v is the scan rate, and d is the laser beam diameter (0.5 mm). The deposited samples were then sliced into 10 mm-thick discs, annealed in a furnace with argon flowing at 500 °C and 1000 °C for 30 min, and cooled to room temperature in the furnace. The laser parameters and heat treatment schedules employed in this study are succinctly outlined [59].

**Figure 7:**
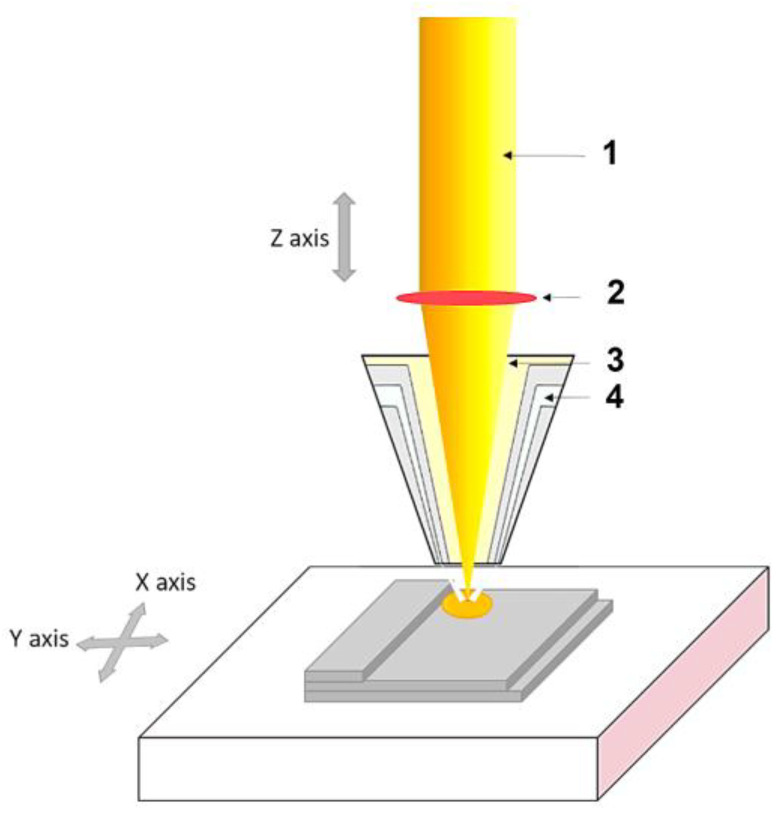
Principal graphical scheme of LENS.

Analyzing Figure 7, it can been seen that the process of laser welding with powder or wire involves generating a laser beam (1) using a laser generator, which is then focused through a lens (2) onto the work piece. To prevent contamination of the welding process, an inert gas (3) is provided to shield both the laser and the melt. At the point of focus, metal powder (4) or wire is introduced, causing the metal powder and workpiece to melt and subsequently build on the surface [60].

This incremental process allows for the step-by-step creation of a cross section of the detail, ultimately leading to the complete detail being produced. Additionally, this method can also be employed for repairing damaged or worn surfaces, a variation commonly referred to as laser welding with powder.

### 2.4. Directed Energy Deposition (DED)

Within the realm of additive manufacturing (AM), Directed Energy Deposition (DED) presents itself as an exceptionally promising technique in terms of build-up rate, capacity to create large components, and ability to repair pre-existing parts. DED processing is frequently employed in the fabrication of the Ti-6Al-4V alloy, which has become a well-established material in the overall AM market. In its as-built state, the tensile properties of Ti-6Al-4V alloy are generally superior to those of conventionally processed materials. However, this comes at the cost of reduced ductility, which remains a significant constraint regarding the alloy’s suitability for various applications [61].

**Figure 8:**
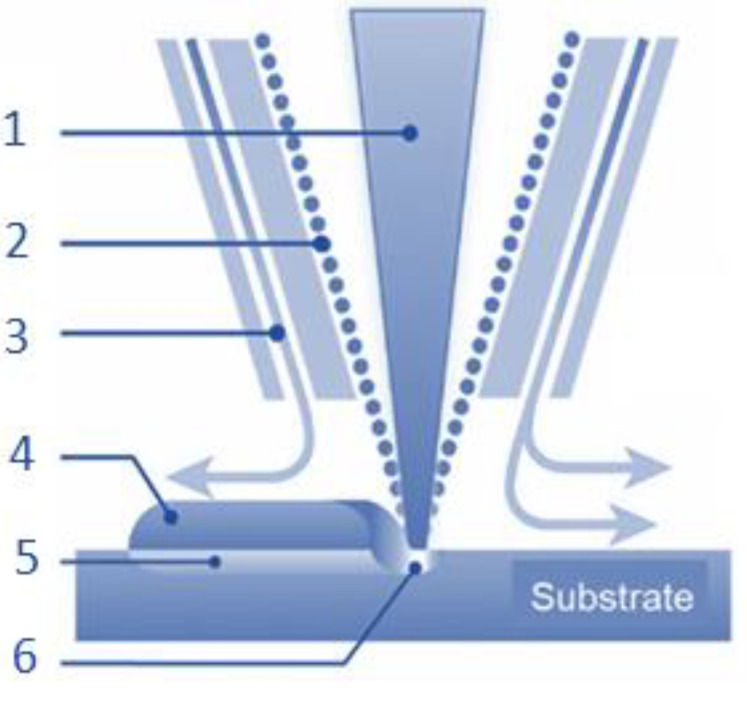
Principal graphical scheme of DED.

This procedural approach of DED is succinctly conveyed through a schematic illustration in Figure 8. The DED process uses three common heat sources: electron beam, electric arc or plasma, and laser (1). In this process, the heat source is typically focused on the point of metal deposition within the feedstock. To guide the deposition process, G-Code is used to control the nozzle of the gun or tool, dictating the specific tool path to be taken around the object [62].

The feed material (2) and heat source are carefully controlled to direct the melt pool along the tool path. The resulting weld pool is subsequently deposited in a precise manner to achieve the desired shape and structure of the object. The DED process is thus highly adaptable and allows for the production of complex geometries with greater control over material properties and structure. In a controlled environment, typically a vacuum or inert gas (3), the feedstock (4), which can be in the form of a metal wire or powder, is melted and deposited onto the object’s surface (5), as illustrated in Figure 8. As with other additive manufacturing methods, the Directed Energy Deposition (DED) process employs a feeder to channel feedstock through a heat source, thus creating a weld pool (6).

A standard Directed Energy Deposition (DED) apparatus comprises a nozzle that is affixed to a multi-axis arm. This arm controls the nozzle’s movements as it deposits molten material onto a targeted surface, where it subsequently solidifies. While this process shares similarities with material extrusion, the nozzle is capable of moving in multiple directions and is not restricted to a specific axis. The deposited material, whether in powder or wire form, can be melted via a laser or electron beam and can be applied from any angle by utilizing four and five axis machines. While DED technology can be used with materials such as ceramics or polymers, it is typically employed with metals.

In order to optimize the process parameters, two distinct laser powers (500 W and 1000 W) were modified in reference [63]. The orientation of the hatches was shifted by 90 degrees for each consecutive layer, and upon depositing a single layer, the laser head would move upwards from the substrate by a distance equivalent to the thickness of the layer. This mechanism would then repeat automatically until the intended build was fully realized.

### 2.5. Direct Metal Laser Sintering (DMLS)

Direct metal laser sintering (DMLS), an additive manufacturing (AM) process, has emerged as a popular technique for the fabrication of functional metallic objects directly from powders. However, a significant challenge facing the industrial application of DMLS is the limited range of materials that can be processed, while still maintaining surface roughness and mechanical properties that are at least comparable to those of wrought materials. To address this issue, the authors of [64] aimed to improve the physical and mechanical properties of components made of 17-4 stainless steel produced by DMLS through grain refinement induced by a shot-peening (SP) process. This process involves subjecting the outer surface layers to severe plastic deformation [64].

The DMLS process utilizes lasers to selectively melt and solidify powdered materials, primarily metals, into desired shapes. However, there are significant differences between DL-PBF and L-PBF techniques:

○ Laser Scanning: In L-PBF, a laser scans the entire surface of the powder bed, melting and fusing the particles together to create a solid part. In contrast, DL-PBF uses a multi-fiber laser head with multiple beams to selectively melt and fuse the powder particles. This allows for greater control over the heat input and leads to a more uniform temperature distribution;
○ Precision: Due to the use of multiple lasers in DL-PBF, the process is generally more precise than L-PBF. This is especially true for parts with fine features or high aspect ratios;
○ Build Time: DL-PBF typically has a faster build time than L-PBF, as the multiple lasers can cover more surface area at once;
○ Materials: L-PBF is generally more versatile in terms of the range of materials that can be processed, including some ceramics and plastics. DL-PBF, on the other hand, is primarily used for metal alloys;
○ Cost: DL-PBF systems tend to be more expensive than L-PBF systems due to the use of multiple lasers and the associated equipment required to control them.

**Figure 9:**
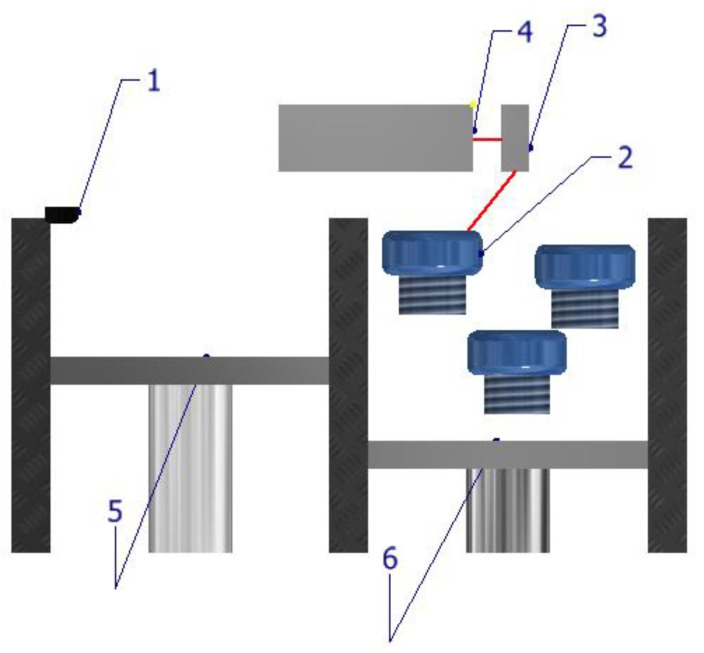
Principal graphical scheme of DMLS.

As can be seen in Figure 9, direct metal laser sintering (DMLS) is a type of additive manufacturing process that involves the sintering of powders and is limited to the fabrication of alloys, particularly titanium-based alloys [64]. Due to the high residual stress and potential distortion, DMLS often necessitates additional support structures during the production process. This technique has found numerous applications in various fields such as the jewelry and dental industries, as well as for the manufacturing of spare parts and prototypes. The material used in powder form is leveled with the coater arm (1). The raw material powder is scanned into printed parts (2). A cross section of the piece is sintered by laser, solidifying. Through a targeting mirror (3), the multi-fiber laser is focused by a laser generator system (4) and passes. The powder reservoir (5) feeds the system with a small amount of powder and then, the construction platform (6) lowers with a layer thickness and a new layer of powder is applied. The process is repeated until a solid part is printed.

### 2.6. Metal Binder Jetting (MJB)

In the domain of additive manufacturing, metal binder jetting (MBJ) has emerged as a viable alternative to laser powder bed fusion (L-PBF) techniques, allowing for the production of intricate geometries at a faster rate and with lower manufacturing costs. MBJ is a two-step process that involves the printing of a green body followed by sintering. In the printing stage, MBJ utilizes a combination of powder bed process and jetting technique. The jetting nozzles distribute an agent, composed of binder and solvent, onto the build platform at the precise location of the part’s contour. This process is repeated layer by layer until the entire 3D contour is formed. The binder serves to bond the powder particles together, resulting in the formation of a green body. The green strength of the part is established through the curing treatment. The next step is to extract the green part from the loose powder in a process commonly referred to as “de-powdering” or “de-caking” [65]. The green parts undergo a debinding step to eliminate the binder, followed by a sintering step to compact the printed parts at high temperatures, during which they are heated in a high-temperature furnace, as part of the ultimate process sequence. 

Currently, stainless steels X2CrNiMo17-12-2 (AISI 316L) and X5CrNiCuNb16-4 (AISI 630), also known as 17-4 PH, are predominantly utilized in the additive manufacturing of steel parts in the MBJ industry. 17-4 PH, a martensitic precipitation-hardening steel, is well-known for its exceptional properties including good corrosion resistance, machinability, and high strength, toughness, and hardness in heat-treated conditions. These properties make it ideal for use in various fields including automotive, aeronautical, maritime, and medical engineering. In fact, 17-4 PH is already a well-established steel for metal injection molding (MIM) processes. Upon precipitation heat treatment, the martensitic structure of this steel is formed with Cu-rich precipitates, as well as Cr-rich phase, Mn-, Ni-, and Si-rich phases [64].

However, the plastic material known as polyamide (PA-12) has found extensive use in various industries such as defense, automotive, and aerospace due to its exceptional properties such as impact strength, toughness, flexibility without breaking, and tensile strength. In addition to being popular for creating prototypes via 3D printing and injection molding, PA-12 has become a popular material for fabricating end-use parts and prototypes through AM fabrication. The utilization of PA-12 in the fabrication of samples has highlighted its mechanical properties, including the ability to print thin and flexible joints [66].

**Figure 10:**
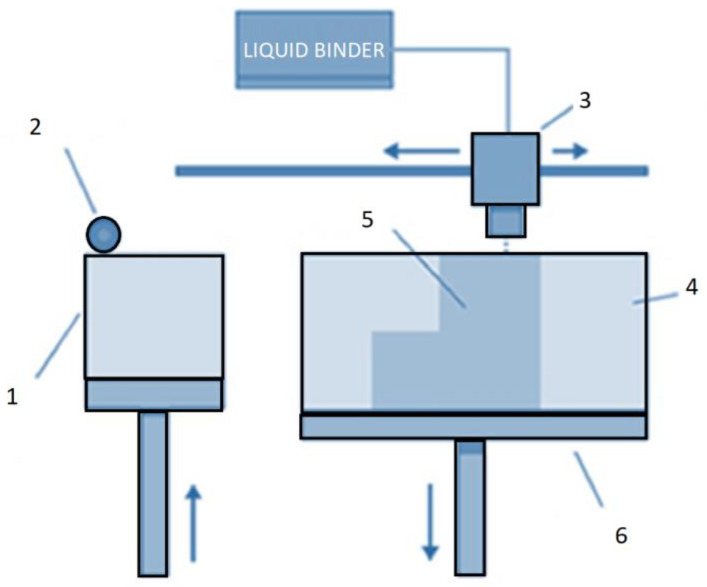
Principal graphical scheme of MJB.

The process of metal binder jetting, graphically described in Figure 10, is a multi-step procedure that involves several intricate steps.

Initially, a layer of powder material is evenly dispersed from the new powder stock (1) utilizing a recoater (2). Following this, the print head (3) is employed to dispense the liquid binder adhesive selectively onto specific areas of the powder layer as required.

After the application of the binder, the build platform (6) is lowered by the thickness of the model’s layer, providing the foundation for the subsequent layer of the powder material. A fresh layer of powder is then uniformly spread over the previous layer, and the cycle of depositing binder, lowering the build platform, and adding a new layer of powder is repeated.

Throughout this iterative process, the powder is precisely deposited and bound in specific areas, forming the desired object (5) where the powder is bound to the liquid binder. Meanwhile, the unbound powder (4) is left intact, encircling the object in position. This methodology is then repeated until the entire object is fully formed, layer by layer, with the final result representing a three-dimensional solid object, precisely constructed with additive manufacturing technology.

### 2.7. Wire-Arc Additive Manufacturing (WAAM)

Wire-based additive manufacturing processes utilize heat sources, such as arc, electron beam, or laser beam, to melt feedstock wires and fabricate freeform 3D components in a layer-by-layer manner at high deposition rates. These processes offer the flexibility to produce large area parts using a wide range of metals or alloys, without the need for molds or dies that may introduce metal contamination and cost. Compared to conventionally cast parts of similar size, wire-based additive manufactured components exhibit refined microstructures with less micro segregation and smaller cell or dendrite spacing, owing to the small size of the liquid melt pool used in the process. In addition, wire-based additive manufacturing methods can be utilized for direct build-up, cladding, and hard facing of existing components, as well as for the repair and maintenance of castings and other metal parts. Moreover, wire-based additive manufacturing has the potential to generate semi-finished products, such as custom plates that can be formed into shape by conventional methods or custom forging blanks with unique shapes that enhance forging properties. However, the thermomechanical post-processing of wire-based additive manufactured parts remains to be thoroughly explored [67]. The gradual buildup of metal parts through the melting of metal wire using an electric arc as the heat source is the essence of WAAM. This method is highly advantageous for producing complex components on a large scale [68]. In WAAM, the heat input is carefully controlled, leading to faster solidification of the melted metal and consequent refinement of the grains. However, upon heat treatment of the alloy, the grains tend to coarsen, and in some cases, the presence of very large grains can be observed.

WAAM is an ideal method for fabricating medium to large-sized components due to its high deposition rates (typically ranging from 2 to 4 Kg/h), exceptional material utilization (>90%), impressive energy efficiency (ranging from 85% to 90%), and expandable working environment. According to research, utilizing the WAAM process can reduce production costs by 7% to 69% in contrast to conventional subtractive techniques such as machining [69].

**Figure 11:**
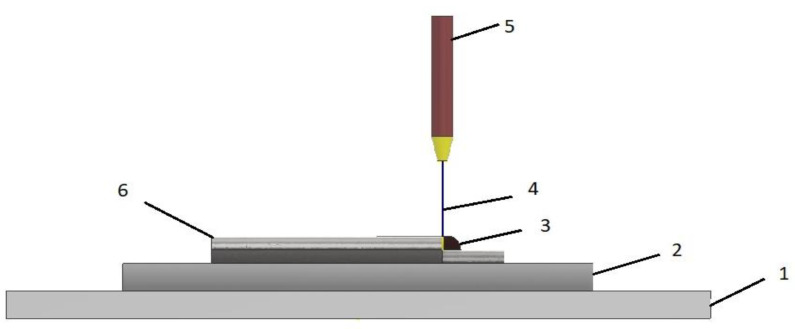
Principal graphical scheme of WAAM.

In Figure 11, the principal scheme of the WAAM is presented. On a work flat (1), a substrate is positioned (2) to be the support to a 3D-printed component, an electric arc to melt and deposit in a melt pool (3) and the metal wire (4). The basic principle of WAAM involves melting the wire through the heat source, the torch (5), and using an electric arc and then depositing it layer by layer to create the desired object (6). This process is commonly used in the aerospace and automotive industries to create large, complex parts quickly and cost-effectively. The WAAM process can be used with a variety of materials, including aluminum, titanium, and steel, and is a promising technology for producing large-scale metal structures with high precision and quality. However, the porosity generated in the WAAM process was detrimental to the mechanical properties of the metal alloy, as noticed in the studied literature [70].

Multidirectional forging and aging treatments are often utilized to adjust the microstructure and mechanical properties of an Al-Zn-Mg-Li alloy produced by wire arc additive manufacturing. This approach was adopted to improve the tensile strength and Young’s modulus of WAAM-produced parts tailored to the need of industries such as the military and aerospace [70].

## 3. Used 3D Printing Materials

### 3.1. Materials Studied and Their Specific Mechanical Behavior with Emphasis on Areas to Improve by Applying Post-Processing Treatments

The materials used for additive technology can be classified into several categories depending on the applications in which they are used, or the printing method used, see Table 1.

Polymers: This category includes a wide range of materials, such as thermoplastics, thermosets, and elastomers. These materials are popular for their ease of use, low cost, and wide availability. Polymers used in 3D printing can be classified into several categories based on their chemical composition and properties. Here is a general classification of polymers used in 3D printing [25,26,46,58,66]:

Thermoplastics: These polymers become soft and moldable when heated and harden when cooled. They can be melted and solidified multiple times without significant degradation of their properties. Some examples of thermoplastics used in 3D printing include:

PLA (polylactic acid)
ABS (acrylonitrile-butadiene-styrene)
PETG (polyethylene terephthalate glycol)
Nylon (polyamide)
PEEK (polyether ether ketone)

Elastomers: These polymers are highly elastic and can be stretched and deformed without permanent deformation. Some examples of elastomers used in 3D printing include:

TPE (thermoplastic elastomer)
TPU (thermoplastic polyurethane)

Other polymers: There are several other types of polymers used in 3D printing that do not fit neatly into the above categories, such as:

PVA (polyvinyl alcohol) support material
PC (polycarbonate)
PEI (polyetherimide)
PPS (polyphenylsulfone)

Metals: Metals are used in 3D printing to create high-strength, durable parts. Common metals used for 3D printing include stainless steel, titanium, aluminum, and copper.

Ceramics: Ceramic materials are used for applications that require high temperature resistance, chemical resistance, and wear resistance. Examples of ceramic 3D printing materials include zirconia, alumina, and silicon carbide.

Composites: Composites are made by combining two or more materials to create a new material with enhanced properties. Common composite materials used for 3D printing include carbon fiber, glass fiber, and Kevlar. These are polymers mixed with other materials, such as metals or ceramics, to achieve enhanced mechanical or thermal properties. Some examples of composite materials used in 3D printing include carbon fiber-reinforced polymer (CFRP), metal-filled polymers, ceramic-filled polymers.

Biomaterials: Biomaterials are used in 3D printing to create tissue and organ replacements. Examples of biomaterials used in 3D printing include collagen, chitosan, and alginate.

The mechanical properties of the materials used in additive technology vary greatly, the main characteristics being presented in Table 2.

Thermal treatments can be used to modify the properties of 3D-printed materials. Here are some examples of thermal treatments that can be used for different types of 3D printing materials:

Polymers: Many thermoplastic polymers used in 3D printing can be annealed, which involves heating the part to just below its melting temperature for a period of time and then slowly cooling it down. This can help to relieve internal stresses and improve the part’s dimensional stability and mechanical properties. Some polymers can also be post-cured, which involves heating the part to a higher temperature to complete the curing process and improve its strength and durability [38,42,43,52,71].

Metals: Metal 3D-printed parts can be heat-treated to improve their mechanical properties. For example, they can be annealed to relieve internal stresses and improve ductility, or they can be quenched and tempered to increase their hardness and strength.

Ceramics: Ceramic 3D-printed parts can be sintered, which involves heating the part to a high temperature to fuse the ceramic particles together and densify the part. This can significantly improve the part’s mechanical properties, such as strength and toughness.

Composites: Composite 3D-printed parts can be post-cured to improve their mechanical properties. The exact type of post-cure treatment will depend on the specific composite material used.

Biomaterials: Biomaterials used in 3D printing can be crosslinked, which involves heating the part to a specific temperature to form covalent bonds between the polymer chains. This can improve the part’s mechanical properties and biocompatibility.

In addition to these specific treatments, some 3D printing materials may also require pre-treatments, such as surface preparation or coating, to ensure optimal adhesion between layers and improve the final part’s properties.

### 3.2. General Characteristics of Polymers Materials Used in Additive Technologies

#### 3.2.1. Polylactic Acid (PLA)

PLA is the most widespread material used in additive technologies and due to its ecological aspect, but it has poor mechanical characteristics and there is research to increase the tenacity of these types of materials. The main properties are briefly presented in Table 3 [27,29,32,39,52].

PLA is a biodegradable and compostable material made from renewable resources such as cornstarch or sugarcane. It is often considered a more eco-friendly alternative to other thermoplastics used in 3D printing.

PLA is a relatively easy material to print with, as it has a low melting temperature (around 180–220 °C) and does not require a heated bed for most prints. It also has a low tendency to warp, which can make it easier to achieve successful prints.

PLA has a relatively low tensile strength compared to other 3D printing materials, typically ranging from 25 to 70 MPa. However, it can be stiffened by increasing its density through annealing.

PLA can be printed with high detail and resolution, making it a popular choice for creating intricate models or prototypes. It is also available in a wide range of colors and finishes.

PLA is generally not recommended for high-temperature applications or load-bearing parts, as it can soften and deform at relatively low temperatures compared to other thermoplastics. It is also not recommended for parts that will be exposed to moisture, as it can degrade over time.

PLA is a popular choice for 3D printing hobbyists and educational settings due to its ease of use, low cost, and eco-friendly properties.

The main applications in which PLA 3D printing material can be used:

Prototyping and product development: PLA is often used for creating prototypes and models due to its ease of use and ability to produce high-detail prints;

Educational settings: PLA is a popular material for use in classrooms and educational settings due to its low cost, ease of use, and eco-friendly properties;

Decorative objects: PLA is available in a wide range of colors and finishes, making it a popular choice for creating decorative objects such as figurines, vases, and jewelry;

Toys and games: PLA is commonly used for creating toys and games due to its safety, durability, and ability to produce intricate designs;

Household items: PLA can be used to create household items such as phone cases, storage containers, and kitchen gadgets, as well as decorative objects such as picture frames and planters.

#### 3.2.2. Acrylonitrile-Butadiene-Styrene (ABS)

Acrylonitrile-butadiene-styrene (ABS) is a thermoplastic commonly used in 3D printing. It is a popular material due to its combination of strength, rigidity, and impact resistance. ABS is also known for its ability to be easily processed, making it a good choice for manufacturing a variety of objects. In 3D printing, ABS is typically used for making functional and durable parts that require strength, such as automotive parts, toys, and electronic enclosures. Its good strength and heat resistance also make it a popular choice for parts that will be exposed to higher temperatures, such as parts for household appliances or electronics, the main properties are briefly presented in Table 4 [43,46,47,66,71].

PEEK is a high-performance thermoplastic polymer that is commonly used in various industries due to its unique combination of properties. It is known for its excellent mechanical, thermal, and chemical resistance properties, which make it a popular choice for applications that require high strength, durability, and resistance to harsh environments. Due to its unique combination of properties, PEEK is often used in demanding applications such as aerospace components, automotive parts, medical implants, and oil and gas industry components. In recent years, PEEK has also become increasingly popular in 3D printing due to its high strength, durability, and resistance to high temperatures, as well as its ability to be processed using a variety of 3D printing technologies, such as ME, SLS, and SLA. The mechanical properties of PEEK are presented in Table 5 [25,38,42,58].

#### 3.2.3. Nylon (Polyamide)

Nylon, also known as polyamide, is a synthetic polymer that is widely used in a variety of industrial applications, including 3D printing. It is a strong and durable material with good mechanical properties and excellent chemical resistance. Nylon is also known for its low friction coefficient and high melting point, which make it suitable for use in applications where high temperatures and wear resistance are important. In 3D printing, nylon is often used to produce functional parts and prototypes that require strength, durability, and flexibility. It is available in a variety of formulations, including nylon 6, nylon 66, and nylon 12, each with slightly different mechanical properties and characteristics presented in Table 6 [31,52].

#### 3.2.4. Polyethylene terephthalate glycol (PETG)

Polyethylene terephthalate glycol (PETG) is a thermoplastic polyester that is commonly used in 3D printing. It is a variation of polyethylene terephthalate (PET) that contains a glycol modifier, which improves its toughness and durability. PETG has become a popular 3D printing material due to its ease of use, low shrinkage, and high impact resistance. It also has a high level of transparency, making it ideal for printing clear or translucent parts. PETG is a food-safe material and is often used for creating food containers and packaging. It is also commonly used in medical and dental applications due to its biocompatibility and resistance to chemicals. The mechanical properties are presented in Table 7 [26,28,36,37].

#### 3.2.5. Thermoplastic Elastomer (TPE)

Thermoplastic elastomers (TPEs) are a class of polymers that exhibit both thermoplastic and elastomeric properties. They are often used as a flexible or rubber-like material in 3D printing. TPEs are composed of a hard plastic phase and a soft rubber phase, which are chemically bonded together to create a material that can stretch and compress in the same way as rubber, but also melt and flow in the same way as plastic when heated. TPEs have several advantages as 3D printing materials, including: flexibility: TPEs are highly flexible and can be used to create parts that need to bend, twist, or stretch, such as gaskets, seals, and phone cases; softness: TPEs have a softer, more rubbery feel than traditional plastics, which can be useful for creating parts that need to be comfortable or non-slip, such as grips or handles; durability: TPEs are resistant to abrasion and tearing, and can withstand repeated flexing and stretching without breaking or degrading; ease of printing: TPEs are relatively easy to print compared to other flexible materials, and can be printed on many standard 3D printers with a direct drive extruder or a flexible filament feeder.

The mechanical characteristics of TPE can vary depending on the specific formulation and processing conditions, but some general properties include:

TPEs typically have a lower tensile strength than traditional plastics, but still have good strength and elasticity. The tensile strength can range from 5 MPa to 30 MPa, depending on the material composition and printing parameters.

TPEs have a high elongation at break, meaning they can stretch significantly before breaking. This property can be useful for creating parts that need to flex or bend repeatedly without breaking. The elongation at break can range from 100% to 1000% or more, depending on the material formulation.

TPEs have a range of hardness levels, typically measured on the Shore A or Shore D scales. The hardness can vary from very soft and flexible (Shore A 10–20) to more rigid and tough (Shore D 50–70). The choice of hardness will depend on the specific application and desired properties of the printed part.

TPEs have good tear resistance, meaning they can resist tearing or ripping under stress. This property can be useful for creating parts that need to withstand repeated bending or twisting, such as hinges or flexible joints.

TPEs can experience some permanent deformation or “set” when subjected to prolonged compression or stress. The amount of compression set can vary depending on the material’s formulation and processing conditions.

#### 3.2.6. Thermoplastic Polyurethane (TPU)

Thermoplastic polyurethane (TPU) is a type of flexible, rubber-like plastic that is commonly used in 3D printing. It is a thermoplastic elastomer, meaning it can be melted and reformed multiple times without degrading its physical properties. TPU has a number of desirable mechanical characteristics for 3D printing, including:

TPU can stretch up to several times its original length before breaking, making it useful for creating parts that need to bend or flex repeatedly without breaking.

TPU is resistant to tearing or ripping, making it a good choice for parts that will be subjected to repeated stresses or impacts.

TPU can recover its original shape after being compressed or stretched, making it ideal for parts that will be subjected to repeated compression or tension.

TPU is resistant to a wide range of chemicals and oils, making it suitable for parts that will be used in harsh or demanding environments.

TPU has a soft, rubber-like texture that can provide a high level of grip and friction, making it useful for creating parts such as phone cases, handles, or grips.

#### 3.2.7. Polyvinyl Alcohol (PVA) Support Material

Polyvinyl alcohol (PVA) is a water-soluble support material commonly used in 3D printing. It is often used in conjunction with other 3D printing materials, such as PLA, to provide support during the printing process. PVA is ideal for creating complex prints with overhangs, bridges, or intricate details that would be difficult to print without support. PVA has a number of desirable properties for use as a support material, including:

PVA dissolves in water, which makes it easy to remove from the finished print without damaging the surface of the main material.

PVA has low adhesion to most materials, which means it can be easily removed from the finished print without leaving any residue or damaging the surface.

PVA is stable and does not degrade over time, which means it can be stored for long periods of time without losing its effectiveness.

PVA can bond well to a variety of materials, making it a versatile support material for a wide range of 3D printing applications.

PVA is non-toxic and biodegradable, making it a safe and eco-friendly alternative to some other support materials.

#### 3.2.8. Polycarbonate (PC)

Polycarbonate (PC) is a strong, durable, and versatile thermoplastic material used in a wide range of industries and applications, including 3D printing. Some of the main characteristics and properties of PC for 3D printing include:

PC is one of the strongest thermoplastics, with high tensile and impact strength, making it suitable for demanding applications that require durability and toughness.

PC has a high glass transition temperature (Tg) and can withstand high temperatures without deforming or melting, making it ideal for applications that require heat resistance.

PC is a transparent material that allows light to pass through, making it suitable for applications such as lenses, windows, and protective covers.

PC is resistant to many chemicals, including acids, bases, and oils, making it suitable for use in harsh environments.

PC has low thermal expansion and contraction, making it suitable for applications that require tight tolerances and precise dimensions.

PC can be easily machined, molded, and 3D printed, making it a versatile material for a wide range of manufacturing processes.

#### 3.2.9. Polyetherimide (PEI)

Polyetherimide (PEI) is a high-performance engineering thermoplastic used in a variety of industries, including the aerospace, automotive, electronics, and medical industries. Some of the main characteristics and properties of PEI for 3D printing include:

PEI has excellent mechanical properties, with high tensile and flexural strength, stiffness, and impact resistance.

PEI has a high glass transition temperature (Tg) of around 217 °C, which makes it suitable for high-temperature applications that require thermal stability and resistance to creep and deformation.

PEI is resistant to a wide range of chemicals, including acids, bases, and hydrocarbons.

#### 3.2.10. Polyphenylsulfone (PPS)

Polyphenylsulfone (PPS) is a high-performance thermoplastic that is known for its excellent thermal and chemical resistance. It is commonly used in the aerospace, automotive, and medical industries, as well as in electronic applications. Some of the main characteristics and properties of PPS for 3D printing include:

PPS has a glass transition temperature (Tg) of around 220 °C, which makes it suitable for high-temperature applications that require thermal stability and resistance to creep and deformation.

PPS is resistant to a wide range of chemicals, including acids, bases, and solvents.

PPS is an excellent electrical insulator, with a high dielectric strength and low dielectric constant and dissipation factor.

PPS has low thermal expansion and contraction, which results in excellent dimensional stability and tight tolerances.

According to [71], annealing heat treatments can be applied to materials such as poly(lactic acid) (PLA), with a density of 1.24 g/cm^3^, melt flow index (MFI) = 6.0 g/10 min (210 °C/2.16 kg), produced by Nature Works, and copolymer ethylene vinyl acetate (EVA), trade name EVA HM728, with a density of 0.950 g/cm^3^, MFI = 6.0 g/10 min (190 °C/2.16 kg), and 28% vinyl acetate content (VA), supplied by Braskem. The tensile modulus, elastic modulus, flexural strength, impact strength of PLA, PLA/EVA blends, and annealed compounds are improved by applying a heat treatment.

Using a temperature for the thermal treatment of annealing [25] of between 75 °C and 125 °C, for materials such as PLA and ABS, demonstrates the fact that no significant increase is obtained in terms of tensile strength, so special importance must be given to the deformations that occur during the heating and cooling the samples subjected to heat treatment. According to [25], an increase in the hardness of the samples was observed mainly due to thermal treatment annealing which leads to the reduction in the level of residual stresses and to a better quality of the joint areas between the deposited layers.

**Figure 12:**
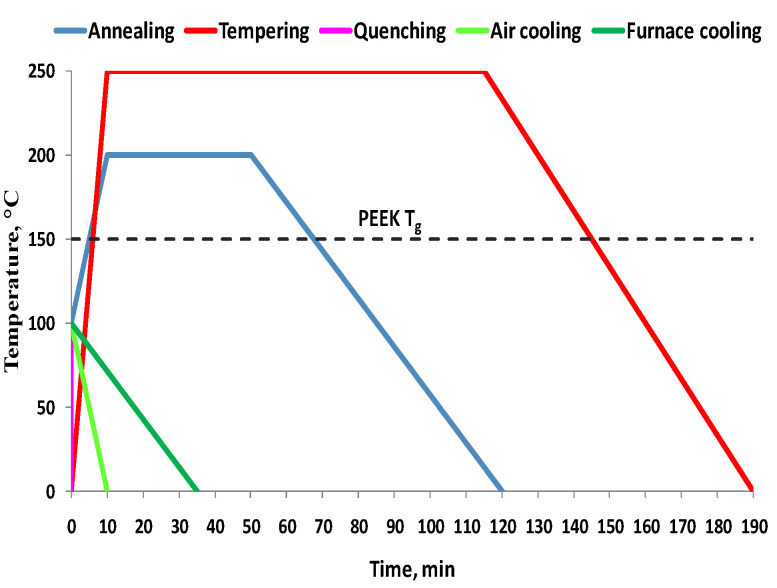
Heat treatments applied on 3D-printed specimens [28].

**Figure 13:**
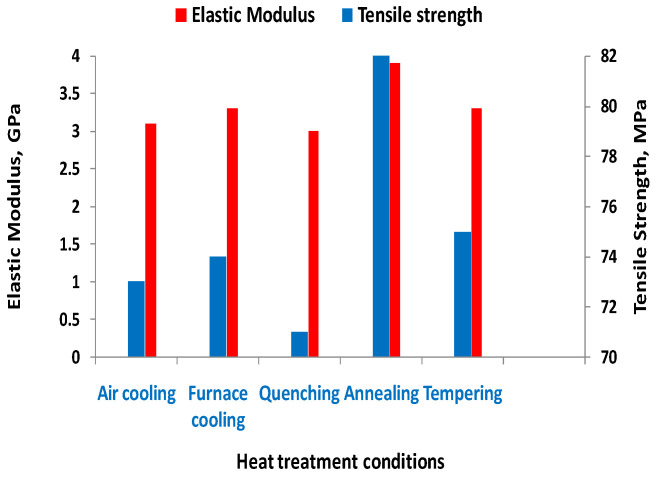
The influence of the type of heat treatment [28].

In [28], the printing parameters were considered as the main factors for the thermal treatment (ambient temperatures, nozzle temperatures, and cooling temperature). In Figure 12, the main thermal treatment schemes applied in the research are represented schematically. The authors of [28] analyzed the influence of several types of thermal treatments on the mechanical properties of PEEK, as shown in Figure 13.

A PLA-type material, printed in the following conditions value layer height 0.15 mm, infill 100%, print speed 75 mm/s, print-bed temperature 30 °C, production time 35 min, was subjected to an annealing heat treatment at 75 °C for 2 h [29]. The effects of applying this type of thermal treatment led to the improvement in the bonding between layers and the creation of a more homogeneous and consolidated material, leading to changes in mechanical properties.

Materials such as PLA/PLA-g-CNFs (Grade: Ingeo Biopolymer 4032D) were purchased from NatureWorks Inc. (Minnetonka, MN, USA). (3S)-cis-3,6-dimethyl-1,4-dioxane-2,5-dione (L-Lactide), tin (II) 2-ethylhexanoate (Sn(OCt)2), and organic solvents (i.e., toluene, acetone, methanol, and chloroform) were purchased from Sigma-Aldrich Inc. (St. Louis, MO, USA). CNFs were purchased from Daicel FineChem Co. (Tokyo, Japan) and were analyzed in [36]. In these pieces of research, the increase in creep resistance before and after annealing was analyzed, observing a slight increase.

Poly(ethylene terephthalate)-glycol (PETG) was used in [37] for evaluating the influence of thermal treatments on the mechanical characteristics. The printing parameters for the heat treatment are presented in Table 8.

The researchers in [37] aimed to increase the mechanical characteristics, but with emphasis on the dimensional changes suffered by the samples during the thermal treatments.

The use of materials such as polyethylene terephthalate glycol (PETG) and carbon fiber-reinforced polyethylene terephthalate glycol (CFPETG) composites, especially in the automobile industry, has become a reality nowadays. The effect of infill density such as 25%, 50%, 75%, and 100% and of thermal treatments was studied in [38] with the results showing changes in hardness (124HRC for 100% infill) and tensile strength as shown in Table 9.

**Figure 14:**
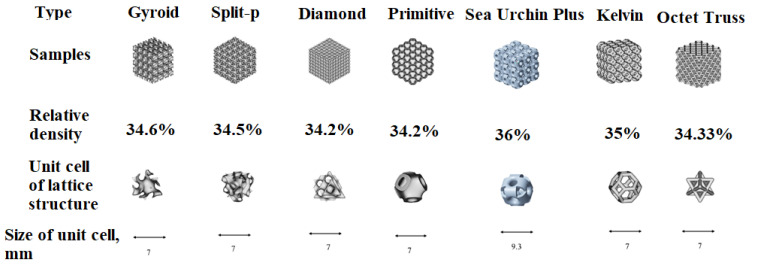
Lattice structures for 3D printing.

The evaluation of PA-12 material—density (g/cm^3^) 1.01; Young’s modulus (MPa) 1437; Poisson’s ratio 0.33; tensile strength (MPa) 27; ultimate tensile strength (MPa) 44—from the point of view of compression behavior was studied according to seven types of lattice structures (presented in Figure 14) which were fabricated by the additive manufacturing method [66]; the mechanical properties were evaluated and compared with annealed (at 110 °C and 130 °C).

Materials such as MAX-G PETG filament from 3DXTECH; CARBONX CFR-PETG filament from 3DXTECH with 20% by weight fiber reinforcement; ECOMAX PLA filament from 3DXTECH; CARBONX CFR-PLA filament from 3DXTECH with 20% by weight fiber reinforcement were used to evaluate the mechanical characteristics [31]. The printing parameters used to make the samples are presented in Table 10.

The improvement of the mechanical properties can be obtained by combining the following parameters’ infill density, annealing, and layer thickness [31]. The printing parameters used in the analysis are presented in Table 11. The main conclusion is that an increase in tensile strength can be obtained but with a decrease in yield strength.

Setting appropriate printing parameters (see Table 12) is very important for materials such as poly(lactic acid) (PLA, Ingeo™ 3D850), pellets containing 0.5% D-isomer, and poly (3-hydroxybutyrate) (PHB) pellets [42].

To identify the temperature of the heat treatment, a cold crystallization peak was used, so that temperatures of 80 °C and 100 °C were used, and holding times of 0.5 h, 1 h, and 2 h were chosen. Making a mixture of PLA and PHB [42] leads to an increase in mechanical characteristics, with possible subsequent heat treatments not leading to positive effects.

Research on materials such as unreinforced Fortron grade PPS, and three short CF-reinforced grades (filler content: 40 wt.%, 50 wt.%, and 60 wt.%) showed that increases in the modulus of elasticity are obtained at temperatures above the glass transition temperature [46]. The main settings taken into account when evaluating these materials are presented in Table 13.

Considering the iso-static compaction (pressure of 0.55 MPa) of samples, studies regarding the influence of the heat treatment temperature (see Table 14) were carried out on an XSTRAND GF30-PA6 type material with a filament diameter of 1.75 mm (the filament is made of nylon matrix reinforced with 30% of glass fibers by weight) and a melting temperature of 206 °C [52]. The printing parameters were as follows: 30 mm/s speed, extruder temperature 250 °C and 90 °C for bed, flow rate 130%, 100% infill, layer thickness 0.2 mm, and nozzle diameter 0.4 mm [52]. Samples compacted at 0.55 MPa at 150 °C have increased in mechanical characteristics (elastic modulus and strength), samples compacted at 170 °C showed a decrease in strength and modulus.

A widely used polymer due to high performance engineering thermoplastics is polyaryletherketone polymer (PAEK). In [47], two materials were studied: Victrex PEEK 151 and Victrex AM 200 whose properties are shown in Table 15. The printing parameters and the temperatures used in the heat treatment for two materials are presented in Table 16.

**Figure 15:**
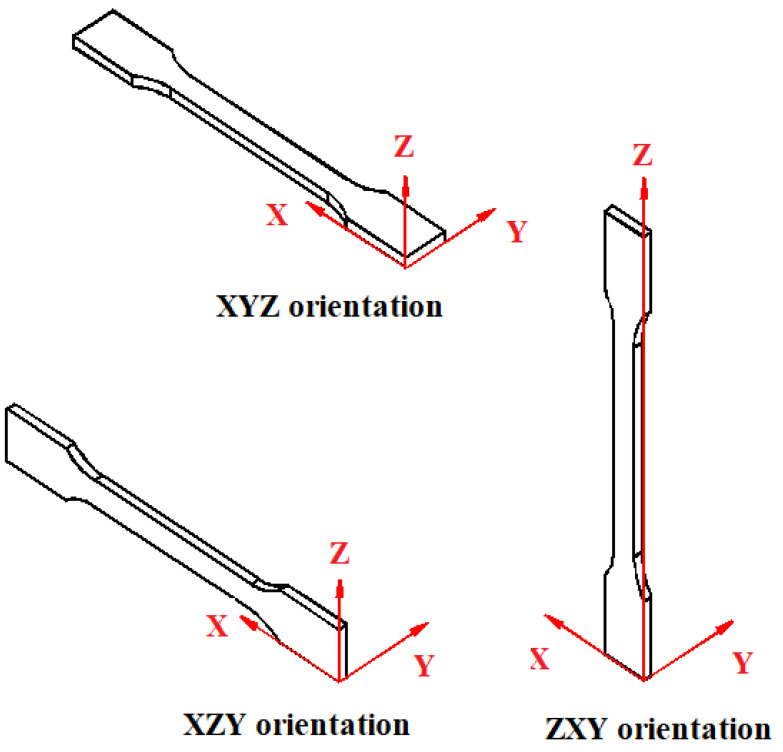
Specimens’ printing orientations.

Effects of thermal annealing on materials such as Spectrum Premium PLA and Prusament PETG Orange were studied from the point of view of the printing orientation of the samples (see Figure 15) [32].

The tensile strength of specimens printed in XZY is almost egal to XYZ, the tensile modulus drops quite a lot (18%). Specimens printed in ZXY have an over 30% increase in tensile strength than the XYZ orientation and around 27% smaller than XZY. The tensile modulus of the specimens printed in ZXY is 15% smaller than specimens printed in XYZ but almost equal to the tensile modulus of specimens printed in XZY [32].

In order to evaluate the ability of 3D-printed high modules carbon fiber-reinforced composite specimens to increase the mechanical characteristics by applying different thermal treatments, the following materials were used [26], as shown in Table 17.

A novelty is the analysis of a type of sandwich material consisting of ABSCF-PLACF-ABSCF and PLACF-ABSCF-PLACF. In order to distribute the temperature evenly and avoid possible deformations, a mixture of sodium chloride powder (table salt), potassium iodate 50 mg/kg, and E536 anti-caking agent is used in the oven chamber [26]. The glass transition temperature and the melting point of the sandwich specimen were considered as an average value of the two materials. Following the application of thermal treatments, significant increases were obtained at temperatures of 150 °C more for the simple material compared to the sandwich type material.

For the comparative analysis of two types of PA12 materials (FX256 and CF15), the second material is a short fiber reinforcement version of the first material and the analysis was carried out for the evaluation of mechanical performance subjected to thermal treatment [43]. The mechanical properties of the analyzed materials (FX256 and CF15) are briefly presented in Table 18, and the printing parameters are shown in Table 19.

Commercial materials of the type 2.85 mm White ABS (melting temperature range is 225–245 °C, Vicat softening temperature is 97 °C) and Pearl White PLA 3D (melting temperature range is 145–160 °C, transition temperature is 60 °C) are used for the study of post-process thermal effects [27]. The conditions for applying the heat treatment are: introduction into a mixture of dry alumina powder with an average grain size of 150 µm and covered by another powder layer of the same thickness; applying a pressure of 12 g/cm^2^ to avoid creep deformations [27]. The samples were used to analyze the influence of the heat treatment on the geometric dimensions and mechanical characteristics, the main conclusion being the fact that geometric variations are directly proportional to the annealing temperature, with a mention of the use of a ceramic powder mold which reduces these dimensional changes.

A superior material in terms of mechanical properties and with chemical and thermal resistance is considered to be polyetherimide (PEI/ULTEM 1010). The properties of the material and the parameters used to configure the printer are presented in Table 20.

A heat treatment temperature value of 225 °C is considered optimal from the point of view of the increase obtained in the case of mechanical test results (tensile test, static bending test, hardness determination) [58].

### 3.3. General Characteristics of Metal Materials Used in Additive Technologies

The mechanical properties of metal materials used in additive manufacturing vary depending on the specific alloy and the printing process used (see Table 21). Some common mechanical properties that are important for metal materials used in additive manufacturing include tensile strength, yield strength, elongation, and hardness:

Stainless Steel—A corrosion-resistant metal that has excellent strength and durability, making it ideal for applications in the medical and aerospace industries.

316 L Stainless Steel: This is the most commonly used stainless steel for 3D printing. It has good corrosion resistance, high strength, and is biocompatible, making it ideal for medical and dental applications.

17-4 PH Stainless Steel: This stainless steel has high strength and is corrosion-resistant, making it ideal for applications in aerospace, defense, and the oil and gas industries.

15-5 PH Stainless Steel: This stainless steel is similar to 17-4 PH, but with better corrosion resistance and improved toughness.

304 L Stainless Steel: This stainless steel is often used for applications requiring high corrosion resistance, such as in the food and beverage industry.

420 Stainless Steel: This stainless steel has high strength and is often used for applications requiring wear resistance, such as in the manufacture of surgical instruments.

Titanium—A lightweight, strong, and corrosion-resistant metal that is commonly used in the aerospace and medical industries for implants and other components.

Ti6Al4V (Grade 5): This is the most common titanium alloy used in 3D printing. It is a combination of titanium, aluminum, and vanadium and is known for its high strength, corrosion resistance, and biocompatibility.

Ti6Al4V ELI (Grade 23): This is a variant of Ti6Al4V that has lower oxygen and iron content, making it more biocompatible. It is often used in medical and dental implants.

TiAl6V4: This is a high-strength titanium alloy that is commonly used in aerospace applications. It has a higher strength-to-weight ratio than Ti6Al4V and is also resistant to fatigue and corrosion.

Ti6Al7Nb: This titanium alloy is used in medical implants and has excellent biocompatibility. It is also resistant to corrosion and wear.

Ti13Nb13Zr: This is a titanium alloy that is often used in dental implants. It has good biocompatibility and is resistant to corrosion and wear.

Inconel—A nickel-chromium alloy that has excellent high-temperature resistance and is often used in aerospace and industrial applications.

Inconel 625: This is the most commonly used Inconel alloy for 3D printing. It has high strength, excellent corrosion resistance, and is often used in aerospace, defense, and marine applications.

Inconel 718: This Inconel alloy has high strength, good corrosion resistance, and is often used in aerospace and gas turbine applications.

Inconel 939: This Inconel alloy has excellent high-temperature strength and is often used in gas turbine engine components.

Inconel 713C: This Inconel alloy has excellent high-temperature strength and is often used in gas turbine engine components.

Inconel 625LCF: This is a low-carbon version of Inconel 625 and has improved fatigue resistance.

Aluminum—A lightweight, strong, and corrosion-resistant metal that is used in the aerospace and automotive industries for producing lightweight parts.

AlSi10Mg: This aluminum–silicon–magnesium alloy is lightweight, strong, and has good corrosion resistance. It is often used in aerospace and automotive applications.

AlSi12: Another aluminum–silicon alloy, AlSi12 is known for its excellent fluidity and castability, making it a good choice for complex geometries.

Al6061: This alloy contains magnesium and silicon, and is known for its strength, corrosion resistance, and weldability. It is often used in structural applications.

Al7075: This aluminum–zinc–magnesium–copper alloy is known for its high strength-to-weight ratio and good fatigue resistance. It is commonly used in aerospace and automotive applications.

Al2024: This alloy contains copper and magnesium, and is known for its good machinability and high strength-to-weight ratio. It is often used in aerospace and structural applications.

Copper—A highly conductive and corrosion-resistant metal that is used for producing electrical components and heat exchangers.

CuNi10: This copper–nickel alloy has good corrosion resistance and is often used in marine applications.

CuCrZr: This copper–chromium–zirconium alloy has good thermal conductivity and is often used in the aerospace industry.

CuSn10: This copper–tin alloy has good wear resistance and is often used in bearing applications.

CuZn39Pb3: This brass alloy contains lead and is often used in low-friction applications, such as valve components.

CuAl10Ni5Fe4: This copper–aluminum–nickel–iron alloy has good corrosion resistance and is often used in marine and aerospace applications.

***Cobalt Chrome***—A biocompatible metal that is used in the medical industry for producing dental implants and other medical devices.

CoCrMo: This cobalt–chrome–molybdenum alloy is commonly used in orthopedic and dental implants due to its excellent biocompatibility and mechanical properties.

CoCrW: This cobalt–chrome–tungsten alloy is often used in aerospace and industrial applications due to its high strength and resistance to wear and corrosion.

CoCrNi: This cobalt–chrome–nickel alloy has good corrosion resistance and is often used in medical implants and dental applications.

CoCrFeNiMn: This cobalt–chrome–iron–nickel–manganese alloy has excellent mechanical properties and is often used in aerospace and automotive applications.

CoCrMoTi: This cobalt–chrome–molybdenum–titanium alloy has good mechanical properties and is often used in dental and medical implants.

The printing parameters for metal 3D printing vary depending on the specific printing technology and metal material used. However, some common parameters that can affect the quality and properties of the printed part include:

Laser power: Typically ranges from 100 to 500 watts, depending on the material and the specific process.

Scanning speed: Can range from 100 to 3000 mm/s, depending on the layer thickness, laser power, and material.

Layer thickness: Typically ranges from 20 to 100 microns, depending on the material and the desired surface finish.

Powder flow rate: Can range from 0.5 to 10 g/s, depending on the material and the specific process.

Bed temperature: Typically ranges from 100 to 400 °C, depending on the material and the specific process.

Laser spot size: Can range from 20 to 100 microns, depending on the desired resolution and the specific process.

Gas flow rate: Typically ranges from 10 to 50 L/min, depending on the specific process and the material being used.

Build chamber atmosphere: Can be controlled with argon, nitrogen, or a combination of gases, depending on the material and the specific process.

Preheat temperature: Typically ranges from 200 to 600 °C, depending on the material and the specific process.

Cooling rate: Can be controlled with the use of a cooling system or by adjusting the laser power and scanning speed, depending on the material and the specific process.

The analysis of the influence of the annealing temperature on some samples made by additive technologies (parameters: layer thickness of 40 μm, laser power of 200 W, hatch spacing of 40 μm, and scanning speed of 740 mm/s) using FeCoCrNi HEA powder (see Table 22 for chemical composition) carried out by the authors of [2] highlighted the importance of temperature, especially due to the phenomenon of grain refinement. The optimal temperature for this type of material, from the point of view of the size of the granulation index, was determined around 700 °C.

The use of high entropy alloys for additive technologies is more and more important nowadays. The alloying elements that have been selected for additive technologies with HEA are Ti, Cr, Mn, Fe, Co, Ni, and Cu (Table 23).

A material that has properties of good thermal strength, weldability, and corrosion resistance is the high aluminum equivalent of near-α titanium alloy, Ti-6Al-2Zr-1Mo-1V [1], used in applications such as load-bearing components of aircrafts, missiles, and launch vehicles with a long-term service temperature of 500 °C. The chemical compositions of the Ti-6Al-2Zr-1Mo-1V alloy wire used are presented in Table 24. The analysis of the mechanical characteristics from the point of view of the annealing temperature has highlighted the fact that significant increases in these values can be obtained if the thermal treatment is performed around 950 °C for 2 h.

A material that has superior mechanical properties by complex microstructural heterogeneity in the as-built conditions and that responds very well to heat treatment is considered to be spherical-shaped (CoCrFeMnNi)99C1 (at%) powders with a particle size in the range of 10–55 µm produced by gas atomization [72].

NiTi is used as shape memory alloys where the shape recovery is initiated by temperature and/or stress. By mixing gas-atomized elemental Ni powder (size range 20–63 μm) and Ti powder (size range 45–105 μm), a material used in additive manufacturing processes can be obtained [63].

The parameters used in 3D printing were: laser scanning speed of 1000 mm/min, a layer thickness of 1 mm, a hatch spacing of 1.5 mm, and a powder flow rate of 5 g/min, and the orientation of hatches was changed by 90° for successive layers. After printing, the samples were subjected to a solution heat treatment in a furnace at 1000 °C for 6 h quenched in water. The NiTi mixture ratio is not well defined; however, an example according to [73] is a blend of Ni and Ti powders with an overall composition of Ni58.0Ti42.0 wt.% (Ni53.0Ti47.0 at.%), the blended NiTi was used for the study of the influence of solutioning and aging heat treatments on the superelastic behavior.

### 3.4. General Characteristics of Composite Materials Used in Additive Technologies

Poly(ethylene terephthalate)-glycol (PETG) is a widely used material in 3D printing technology (Table 25), due to its various advantageous properties such as chemical alkali resistance, transparency, gloss, low haze, and good printability. Its popularity stems from its versatility and suitability for a range of applications.

Moreover, by incorporating carbon fiber into PETG, its potential applications expand even further. The resulting composite exhibits enhanced strength, resilience, and a reduced risk of warping. This makes it an excellent choice for automotive and other industrial applications where durability and robustness are crucial.

Additionally, when reinforced with aramid fibers, PETG can be utilized in sectors that demand high resistance to friction and impact. This further broadens its potential uses across various industries [37].

The main conclusion of the heat treatment temperature study for composite materials is that hardness increases with increasing temperature and exposure time (Table 26).

In terms of flexural strength, it is possible to observe different effects of the annealing treatment on this mechanical property. For PETG, for example, increasing the temperature increases the bending strength, but for all temperatures, when they remain constant and the exposure time increases, the bending strength decreases [37].

Poly(ethylene terephthalate)-glycol (PETG) and carbon fiber-reinforced polyethylene terephthalate glycol (CFPETG) composites have become the excellent material choice for automotive and other industrial applications in the desktop-based material extrusion (ME) technique. PETG and CFPETG were the filaments used for printing the specimens. PETG was found to be strong and cost-effective when compared with acrylic and polycarbonate. Its unique characteristics make it preferable for impact-resistant glazing applications. In order to improve its strength, 20 wt.% of carbon fibers were blended and taken as a CFPETG filament. Since the presence of chopped carbon in PETG increases melt viscosity, changes coefficients of thermal expansion, and increases the ability to withstand heat [38]. Table 27 shows the mechanical characteristics of the materials used [38].

**Figure 16:**
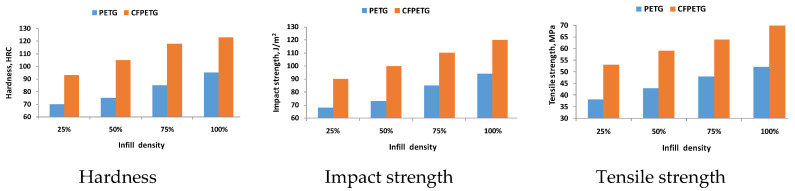
Printed specimen of annealed PETG and CFPETG mechanical characteristics [38].

The values of the mechanical characteristics for PETG and CFPETG specimens annealed with different percentages of filler 25%, 50%, 75%, and 100% are shown in Figure 16.

On comparing the mechanical properties of both annealed PETG and CFPETG specimens, it was found that annealed CFPETG specimens had higher properties because of carbon fiber content [38].

Carbon fiber-reinforced polymer composites (CFRP) have become integral to high-performance markets such as aerospace, energy, and automotive where mass savings are critical. The additively manufactured continuous carbon fiber-reinforced PEEK composites were manufactured using a 5-axis robotic ME printer, who provided details of the composite filament and print conditions. The AS4C carbon fiber volume fraction of the composite filaments was ~42% [53].

## 4. Common Post-Processing Technologies Used in Additive Technology

Heat treatments are technological processes consisting of successive heating and cooling cycles with the aim of modifying the properties of materials. Heat treatments can have various effects on the microstructure and properties of materials, including changes in mechanical properties, in particular hardness, strength, ductility, toughness and corrosion resistance. Some of the most common types of heat treatments applied to materials are listed below.

Annealing is a heat treatment in which a material is heated to a specified temperature and then slowly cooled. Annealing is used to reduce the hardness and increase the ductility of materials, making them easier to work.

Hot isostatic pressing (HIP) is a manufacturing process that is used to consolidate and densify materials such as metals, ceramics, and composite materials. It involves subjecting the material to high temperatures and pressure in a gastight container, typically an inert gas such as argon, helium, or nitrogen.

HIP involves placing material in a metal canister or vacuum chamber, which is filled with gas to a specified pressure and temperature. The material undergoes high pressure isotropic compression, which helps eliminate voids or defects in the material. The temperature used during the process is typically close to the melting point of the material, which allows the material to achieve a maximum level of density and uniformity. HIP is often used for the production of parts with complex geometries that are difficult to produce by other methods such as casting or forging, and is used for high performance materials. It is also used in the production of high performance materials that have high density and uniformity requirements, such as aerospace components and medical implants. HIPs are widely used in aerospace, medical, and automotive applications to produce high quality, dense parts with excellent mechanical properties.

Aging heat treatment is the process of heat treating a metallic material to improve its mechanical and physical properties by increasing its structural stability and eliminating internal stresses. The process involves heating the material to a high temperature for a specified period of time, followed by cooling at a controlled rate. Generally, this process is used to improve the material’s resistance to corrosion and bending, as well as to increase hardness and wear resistance. There are two main types of aging heat treatment: natural aging and artificial aging. Natural aging involves allowing the material to rest at room temperature for a period of time to reach its final aged state. Natural aging is a slower process and can take anywhere from a few days to a few weeks, depending on the material. Artificial aging involves heating the material to a high temperature for a period of time, followed by rapid cooling. Artificial aging is faster than natural aging and can be completed in a few hours. Thermal aging is used in the aerospace, automotive, and other industries that use high performance metallic materials.

Quenching involves heating a material to a certain temperature and then rapidly cooling it. Cooling is achieved by immersing the material in a coolant, usually water or oil. The quenching process is usually used to increase the hardness of the surface layers while maintaining the strength of the layers, but it can also make them more brittle.

Normalizing is a heat treatment in which a material is heated to a temperature above its critical carbon point and then cooled in still air. To increase strength and ductility, the normalizing process is used to refine the grain structure of materials.

Stress relief involves heating a material to a specified temperature and then cooling it slowly. The technique reduces residual stresses, increasing dimensional stability, crack resistance, and distortion resistance.

Tempering (stress relieving) is a process of controlled heating and cooling of a material after it has been subjected to mechanical stress or another heat treatment, such as quenching. The purpose of this process is to reduce internal stresses and improve the mechanical properties of the material, such as ductility and toughness. Tempering may be used after hardening to reduce the brittleness of the material, in which case this combination of two heat treatments is called tempering.

In general, heat treatment processes are an essential part of materials engineering and are used to improve the performance and reliability of a wide range of materials.

There are many types of heat treatments that can be accompanied by other generally mechanical technological processes, such as plastic deformation, which are applied for the specific purpose of modifying a range of desired mechanical properties in certain engineering applications. The following is a review of these treatments and their characteristic parameters using the studies and articles that formed the basis of the selective bibliography.

For the purpose of this review, 100 representative papers in the field of post-processing treatments applied to parts produced by means of additive technologies have been analyzed. The considerations relating to the post-processing treatments used for the parts produced by the additive technologies have been summarized in Table 28.

As highlighted in the summary in the table above, parts produced by additive manufacturing can have residual stresses and microstructural defects that can affect their mechanical properties. Heat treatment, particularly annealing, is a common method of improving the mechanical properties of additive manufactured parts. The following sections provide an analysis of why annealing is necessary for additive manufactured plastic and metal parts.

In additive manufacturing, parts are built up layer by layer. This process can create residual stresses in the material due to uneven heating and cooling of the layers. Residual stresses can cause deformation, cracking, and failure of the part in use. Annealing can reduce the residual stresses in the material, making it more stable and less prone to deformation and failure.

Additive manufacturing can also introduce other types of microstructural defects into the material, such as porosity, uneven grain structure, and inclusions. These defects can reduce the mechanical properties of the material, such as material strength and fatigue strength. Annealing can help reduce these defects by increasing grain size, eliminating inclusions, and reducing porosity. This can improve the mechanical properties of the material, making it stronger and more durable.

Plastics are widely used in additive manufacturing because of their low cost, ease of processing, and ability to be molded into complex shapes. However, plastic parts produced by additive manufacturing can contain internal stresses and microstructural defects that can affect their mechanical properties. Annealing can help reduce these defects and improve the mechanical properties of the plastic part. Annealing parameters such as temperature and holding time depend on the type of plastic used and the desired mechanical properties.

Metal parts produced using additive manufacturing technology can also contain residual stresses and microstructural defects due to cooling and solidification in different areas of the metal. Annealing can help reduce these defects and improve the mechanical properties of the metal part. The annealing temperature and time will depend on the type of metal used and the desired mechanical properties.

The literature reviewed shows that annealing can have a significant effect on the mechanical properties of additive manufactured parts. It can increase the ductility, toughness, and fatigue strength of the material, making it more durable and resistant to failure. The effect of annealing on mechanical properties depends on the annealing temperature, time, and cooling rate or medium.

Annealing is often used in combination with other finishing techniques such as machining, polishing, and surface treatment. This can further enhance the mechanical properties of the part and improve the surface finish.

Annealing heat treatment is a necessary process for parts produced by additive technology in plastics and metals. It can help reduce residual stresses and microstructural defects, improve the mechanical properties of the material, and increase its durability and resistance to failure. The annealing temperature and time depend on the type of material used and the desired mechanical properties.

Hot Isostatic Pressing (HIP) is a post-processing technology that can be used to improve the quality and performance of parts produced by various types of additive technologies. The reasons why HIP is beneficial for both plastic and metal parts produced by different additive technologies are outlined below.

Parts produced by various additive technologies are prone to porosity due to the layer-by-layer deposition process. This can lead to defects such as voids, cracks, and inclusions that can weaken the part and reduce its mechanical properties. HIP can be used to reduce porosity and improve hardness by applying a high temperature and pressure to the part. The high pressure forces the gas to diffuse out of the part, while the high temperature promotes plastic deformation and defect healing, resulting in a denser and more uniform microstructure.

Additively manufactured parts often exhibit lower mechanical properties compared to conventionally manufactured parts due to their microstructural anomalies. HIP treatment can improve the mechanical properties of additive manufacturing parts by eliminating or reducing defects such as porosity, inclusions, and microcracks. This leads to an increase in strength, ductility and hardness, making the parts more suitable for demanding applications.

In addition, HIP treatment can be used to improve the surface finish and aesthetics of additive manufacturing parts by applying high temperature and pressure to the part, resulting in plastic deformation of surface irregularities and creating a more uniform surface. This can make the part more visually appealing and suitable for applications requiring a smooth surface finish.

Parts produced using additive technologies often contain residual stresses due to the thermal cycling involved in the printing process. These stresses can affect the mechanical properties of the part and lead to deformation or failure under stress. HIP treatment can be used to remove residual stresses by applying a high temperature and pressure to the part, which promotes plastic deformation and stress reduction.

HIP treatment can be used to achieve certain material properties of parts produced by additive technologies by strictly controlling the parameters applied during the process, namely pressure and temperature. This allows the desired microstructure and part properties to be achieved for a specific application.

To improve the quality and performance of parts produced by additive technologies, HIP treatment is a valuable post-processing technique. It is capable of reducing porosity, enhancing mechanical properties, improving surface finish and appearance, removing residual stresses, and adjusting matrix properties. Due to their tendency to exhibit porosity and microstructural anomalies, HIP treatment is particularly beneficial for additive plastic and metal parts. As the industry continues to embrace additive technologies, HIP will become increasingly important to help ensure the quality and reliability of these complex parts.

Aging treatment is a process that can be applied to parts that have been manufactured using additive technology in order to improve their properties and performance.

Additive manufacturing is the production of parts with anisotropic properties, i.e., the properties are different in different directions. The reason for that lies in the fact that the part will be produced in layers, and each layer’s properties may differ. Aging can improve the mechanical properties of parts, such as strength, toughness, and ductility, by homogenizing the microstructure of the part. The treatment can also reduce residual stresses in the part, which can be the cause of deformation or cracking.

Due to the layer-by-layer manufacturing process, parts produced by additive technology can have a rough surface finish. By removing roughness and smoothing the surface, aging can improve the surface finish. This can be particularly important for parts that have high surface finish requirements, such as aerospace or medical applications.

The corrosion resistance of parts produced using different additive technologies may be lower than that of parts produced using more traditional manufacturing methods. This is because the layering process may lead to different structures and compounds in different areas of the part, increasing the susceptibility to corrosion. By homogenizing the microstructure and composition of the part, aging can improve the corrosion resistance of the part.

To improve the properties of metal parts, heat treatment is a commonly used process. However, parts produced by additive technology may be non-uniform in structure and this may reduce the efficiency of heat treatment. Aging treatment can be used to homogenize the microstructure of the part and make the heat treatment more effective.

However, ensuring the consistency of the properties of complex geometries and customized parts can be a challenge. Age hardening can be used to achieve consistency of part properties by homogenizing the microstructure and eliminating any defects which may exist.

Aging treatment is necessary for parts made by additive plastic and metal technology to improve their properties and structure as well as their performance. The treatment can improve mechanical properties, improve surface finish, increase corrosion resistance, improve heat treatment, and achieve consistency of part properties. By applying aging treatment, manufacturers can ensure that parts made by additive technology meet the required specifications and perform as expected.

Complex heat treatments are sequences of heat treatments that can improve the properties of additive manufactured parts, especially those made of plastics and metals. The benefits of complex heat treatments are summarized below.

Complex heat treatments can improve the strength and durability of additive manufactured parts. The process involves passing the material through different cycles of heat treatment. These can change the microstructure of the material, resulting in improved mechanical properties such as increased strength, toughness and wear resistance.

Complex heat treatments can also improve the surface properties of additive manufactured parts. This is particularly important for metal parts that are subject to corrosion and wear. Processes such as hardening, nitriding, and carburizing can modify the surface of the part to improve its resistance to corrosion, wear, and fatigue.

Due to the rapid cooling of the deposited material, additive technologies can create residual stresses within the material. These residual stresses can have an effect on the mechanical properties of the part and cause deformation or cracking. Complex heat treatments can help reduce these residual stresses by annealing the material, which involves heating it to a certain temperature, then slowly cooling it and reheating. This can result in a more uniform microstructure and reduce the likelihood of deforming or cracking.

In parts produced by additive technology, complex heat treatments can be used to achieve specific properties. For example, age hardening may increase the strength of certain metal alloys, and quenching may increase the hardness of steel. Specific properties to meet the requirements of particular applications can be achieved by carefully controlling the sequence of heat treatment processes.

The dimensional stability of parts produced by additive technology can also be improved by other complex heat treatment sequences. For parts requiring tight tolerances or subject to thermal expansion, this is particularly important. Further heat treating sequences followed by annealing the material can reduce residual stresses that can cause distortion, resulting in a more stable and accurate part.

## 5. The Influence of Post-Processing Treatments on the Mechanical Properties of 3D Printed Parts

Post-processing heat treatment is a common method used to modify the tensile properties of 3D-printed parts. Heat treatment involves subjecting the printed parts to controlled temperature conditions for a specific duration, resulting in changes in the material’s microstructure, crystallinity, and mechanical behavior. The effects of post-processing heat treatment on the mechanical properties of 3D-printed parts can vary depending on several factors, such as the material type, printing process, heat treatment parameters, and part geometry.

One of the primary effects of post-processing heat treatment on mechanical properties is the improvement in strength and toughness. Heat treatment can reduce residual stresses within the printed parts, leading to enhanced tensile strength and elongation at break. Additionally, heat treatment can promote the recrystallization or annealing of the material, resulting in a refined grain structure and increased toughness, which can improve the ability of the printed parts to withstand applied loads without failure.

The temperature and duration of the heat treatment process are critical parameters that can influence the tensile properties of 3D-printed parts. Different materials have different thermal properties, and the optimal heat treatment parameters may vary accordingly. Overheating or prolonged exposure to high temperatures can result in material degradation or even melting, leading to a decrease in tensile properties. On the other hand, inadequate heat treatment may not induce the desired changes in the material’s microstructure, resulting in minimal improvement in tensile properties.

The effects of post-processing heat treatment on mechanical properties can also be influenced by the specific printing process used, such as material extrusion (ME), stereolithography (SLA), or selective laser sintering (SLS). For instance, ME-printed parts made from thermoplastic materials can be heat treated to improve their tensile properties due to the melting and crystallization behavior of the thermoplastics. In contrast, SLA-printed parts made from photopolymer resins may require different heat treatment conditions due to their unique chemistry and curing process.

In the scientific literature, many studies have investigated the influence of different heat treatments on 3D-printed parts of different type of materials such as PLA [26,31,32,41,48,49,50,55,57,71], ABS [26,27,33,42,51,54], nylon [43], PPS [56], PEEK [28,47], PETG [32,37,38,39], metals (steel [5,6,7,9,10,11,34,64,65,67,75,76,76,77,90,93,99], aluminum [23,24,70,82], Inconel [23,24,95], titanium [8,13,16,17,18,19,21,61,84,97,101]), and composites [26,41,49,52,53].

The results from [71] demonstrate that the tensile strength of the investigated compounds decreases when EVA is added, with a more pronounced decline observed at a content of 30%. For the annealed samples, the results of untreated and treated blends are similar, except for PLA, which shows an increase in tensile strength from 60 to 68.5 MPa, indicating the improved crystallinity and strength of PLAT. The addition of 30% EVA results in a significant increase in elongation at break, from 10.8% to 14.8% (37% increase) compared to PLA. However, the addition of 20% EVA is not sufficient to promote a significant increase in elongation at break. For the annealed compounds, the elongation at break decreases due to the development of a crystalline structure, which allows for less deformation.

The effect of annealing on the mechanical properties of PLA-shape memory polymer is investigated in [4]. The results revealed consistent behavior in the annealed samples, with improved ultimate tensile strength (UTS) and elastic modulus compared to the as-printed samples. Specifically, the average UTS for the as-printed samples was 33.83 MPa, while the annealed samples showed an average UTS of 44.06 MPa, representing a notable increase of 30.25%. Additionally, annealing caused a 19% increase in the average elastic modulus, from 1.35 GPa for the as-printed samples to 1.67 GPa for the annealed samples.

The PLA objects printed with 90% infill density and a layer thickness of 0.2 mm exhibit the highest ultimate tensile strength, namely 48.812 MPa [31]. Furthermore, annealing the specimen at 120 °C results in a further increase in strength of 4.7%. In contrast, the objects printed with 60% infill density and a layer thickness of 0.2 mm presented the highest yield strain. However, upon annealing, a decrease in yield strain was observed. Regarding the total energy absorption, the objects printed with a 90% infill density and a layer thickness of 0.3 mm exhibit the highest value of 5.817 J/s, but, annealing the specimen leads to a decrease with 30% in total energy absorption.

Annealing PLA ME specimens printed in ZXY orientation at a 60 °C temperature for 1 h results in a 23.8% increase in tensile strength and a 6.84% decrease in tensile modulus. For 80 °C annealing for one hour, tensile strength is not changing and the tensile modulus decreases by 9.68% [32].

In [26], various combinations of materials were used, namely PLA-reinforced with 9% carbon fiber (PLACF), and ABS reinforced with 9% carbon fiber (ABSCF). It was observed that as the heat treatment temperature increased, the porosity in the samples decreased. Additionally, a decrease in infill density of up to 70% led to an increase in porosity. The stiffness and strength of the heat-treated samples were significantly higher than those of the non-treated samples. The highest tensile strength of 257.4 MPa was observed in the heat-treated PLACF sample with 100% infill density at 150 °C, while the lowest value of 15.02 MPa was found in the heat-treated ABSCF sample with 100% infill density at 50 °C. At 150 °C, there was a remarkable improvement in the tensile strength, with 155.24% increase for the ABSCF-PLACF-ABSCF sample with 100% rectilinear infill. The heat-treated samples also displayed an increase in Young’s modulus of elasticity (*E*), with a 12.5% increase observed in the PLACF-ABSCF-PLACF sample with 100% Archimedean chords infill at 150 °C. The results indicate a significant correlation between the heat treatment temperature and tensile properties, as the improvement in the tensile strength and modulus of elasticity was limited for samples treated at low temperatures compared to those treated at high temperatures. The maximum tensile strength of untreated samples was 151.7 MPa in the PLACF sample with 100% infill density, while the minimum value of 54.39 MPa was observed in the ABSCF-PLACF-ABSCF sample with 70% infill density.

The tensile properties of the PLA and PLA/CF filaments [48] showed that the addition of carbon fibers to the PLA filament led to a significant increase in elastic modulus of the samples. The annealing has little to no effect on the elastic modulus for specimens of the same material (for PLA, the elastic modulus increased with 7.71% at annealing temperature of 85 °C and with 9.19% at 145 °C; for the PLA/CF specimens, the elastic modulus increased with 10.38% at 85 °C, 18.48% at 115 °C, and 12.32% at 145 °C), while an increase in elastic modulus was observed between the PLA and PLA/CF specimens. The UTS was relatively constant across all specimens, regardless of material or annealing treatment (for PLA, the UTS increased with 8.43% at annealing temperature of 85 °C and 2.02% at 145 °C and decreased with 3.2% at 115 °C; for the PLA/CF specimens, the UTS decreased with 2.7% at 85 °C, increased with 9.78% at 115 °C, and with 4.52% at 145 °C). The ultimate strain of PLA specimens was consistently higher than for the PLA + CF specimens, but remained unaffected by annealing for specimens of the same material.

The outcomes of the experiments including thermal aging [55] indicated that the strongest specimens were the 0° layer specimens without defects and no thermal aging. On the other hand, the weakest specimens were the ones with a defective 90° layer and thermal aging, indicating that defects perpendicular to the loading direction had a more significant impact on the tensile strength in comparison to defects along the loading direction.

The objective of the authors of [57] was to investigate how thermal annealing impacts the performance of ME-printed PLA material under static and dynamic bending loads. -A tendency for *E* to increase was observed as the annealing time and temperature increase, while the flexural strength and failure strain had a decreasing trend. The average increase in *E* was up to 21.24% and a reduction of 56.59% and 65.18% in flexural strength and failure strain, respectively, was experienced.

**Figure 17:**
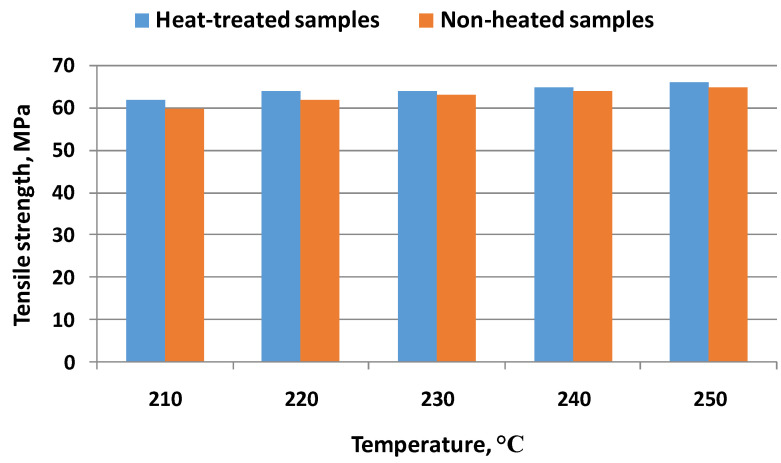
The influence of heat-treatment on tensile strength.

Akhoundi et al. [50] examined how variations in nozzle temperature and heat treatment influenced the tensile strength and modulus of high-temperature polylactic acid (HTPLA) parts. For printing temperatures of 210 °C, 220 °C, 230 °C, and 240 °C, the increases in tensile modulus were 15%, 17%, 32%, and 26%, respectively, compared to the non-heat treatment mode. At a printing temperature of 210 °C, the maximum strength enhancement of 7% was obtained (Figure 17).

The elastic modulus and ultimate strengths increased by 37% and 4%, respectively, when annealing the CF/PLA 3D composite [41].

The conclusions from [33] indicated that temperature has a significant impact on the enhancement of UTS for ABS ME-printed parts, with an average increase of 89%, observed at an annealing temperature of 160 °C.

To assess the efficacy of the warm isostatic pressure (WIP) process, tensile tests were conducted in [54] on standard specimens that were subjected both to annealing and WIP process. These specimens were printed with varying build orientations and infill patterns, including different raster angles, in order to evaluate the potential reduction in anisotropic properties through the utilization of the WIP process. For the specimens fabricated in the flat direction, the annealing results in decreasing the UTS by 1.8%… 10%, depending on the raster angle. Only for the sample with 90° raster angle was a UTS obtained greater by 30.8% compared with the as-built sample. For the specimens fabricated in the upright direction, greater values (with 9%… 35%), of UTS were obtained, for different values of raster angle. The WIP procedure determines a notable increase in the mechanical properties of the samples. In comparison to the as-built specimens, the samples fabricated in the flat direction presented an increase in UTS of 9.15%… 58.33%, while the UTS of the samples fabricated in the upright direction was greater by 29.9%… 78.16%.

**Figure 18:**
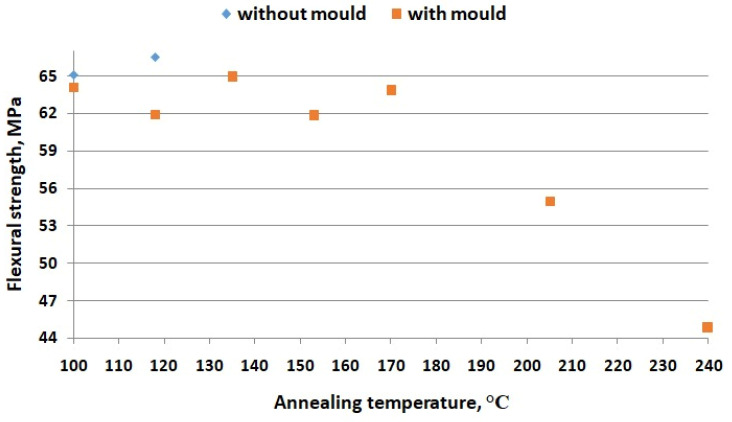
ABS flexural strength.

Comparing the treated specimens to the untreated ones (as shown in Figure 18), it can be observed that the improvement is lost from 170 °C and deteriorates significantly, with a premature failure of the specimens occurring from 205 °C onwards [27].

Yi et al. [47] conducted a complex study regarding the influence of low annealing temperature (LAT) and high annealing temperature (HAT) on the mechanical properties of PEEK51 and AM200 materials, considering a different printing orientation, as presented in Table 29 and Table 30.

It can be observed that mechanical properties of PEEK51 and AM200, including elastic modulus, strength, and elongation at break, are strongly influenced by the orientation and post-processing conditions.

For 3D-printed nylon glass fiber composites subjected to annealing and isostatic compaction, the results of the authors of scientific paper [52] indicate that, by selecting an appropriate isostatic compaction temperature, both strength and modulus in principal directions can be significantly improved. Strength was improved by over 50% and 100% at 0° and 90° printing orientation, respectively, and a 200% increase in the modulus in 90° printing direction was found for samples compacted at 0.55 MPa and 200 °C.

The hot isostatic pressing of additively manufactured continuous carbon fiber-reinforced PEEK composite resulted in a maximum increase for inter-laminar shear strength (30%) and flexural strength (46%) for a treatment temperature of 250 °C. The compressive strength and modulus showed an increase of 18% and 8%, respectively, compared to the reference samples. Similarly, an increase was obtained of 15% and 7% for the tensile strength and the tensile modulus, respectively [53].

The objective of study [53] was to investigate the impact of thermal annealing on the flexural properties of PETG and PETG reinforced with carbon (CFPETG) and aramid fibers (KFPETG). The results revealed that higher temperatures and longer exposure times during thermal annealing led to a substantial improvement in flexural strength and modulus for both CFPETG and KFPETG composites. Specifically, the flexural strength increased by approximately 31.8% and 11.1%, while the flexural modulus increased by approximately 61.1% and 46.7% for CFPETG and KFPETG composites, respectively, compared to untreated specimens.

**Figure 19:**
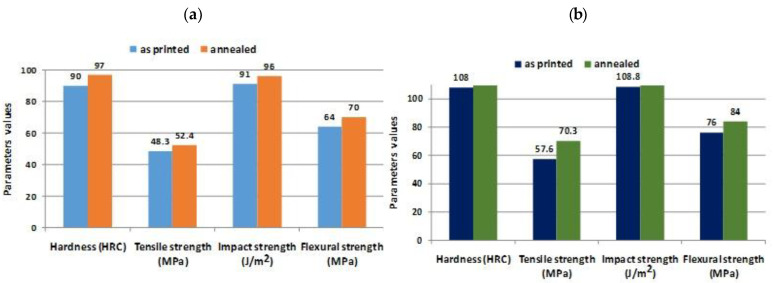
Mechanical properties for as-printed and annealed specimens made of PETG (**a**) and CFPETG (**b**).

The comparison from Figure 19 of mechanical properties between annealed PETG and CFPETG specimens revealed that the annealed CFPETG specimens exhibited superior properties due to the presence of carbon fibers. Specifically, the as-printed and annealed CFPETG specimens with 100% infill density showed significant increases of 21% in hardness, 25% in tensile strength, 23% in impact strength, and 18% in flexural strength compared to the annealed PETG specimen with 100% infill density [38].

A similar investigation was conducted by the authors of [39] in order to improve the interlayer tensile strength of 3D-printed composites made from short carbon fiber-reinforced PETG and PLA materials, through the process of annealing and the effects are summarized in Table 31.

The findings of the study [43] revealed that CF15 nylon exhibited, due to annealing treatment, an increase of approximately 11% in its tensile modulus, strength, flexural modulus, and flexural strength. In contrast, FX256 showed similar values for tensile properties but demonstrated a significant improvement in flexural results, with doubled values compared to CF15.

**Figure 20:**
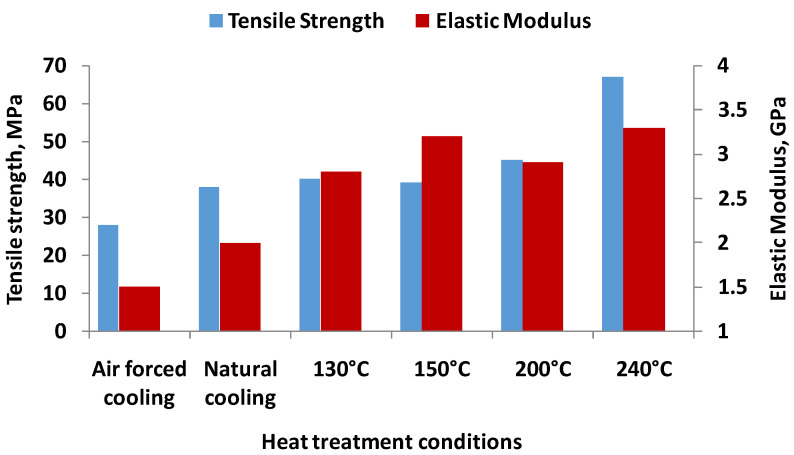
Tensile properties of 3D-printed PPS samples.

The PPS sample’s tensile strength was found to be up to 108% higher when treated at 240 °C, and the elastic modulus was greater by 80% [56]. The strengths and elastic modulus of the 3D-printed samples increased proportionally with the increase in the heat treatment temperature, as shown in Figure 20.

For 316L steel synthesized by L-PBF [6], it was observed that the strength of the specimens decreased with higher annealing temperatures, and this fact can be attributed to microstructural coarsening, suggesting that the optimal combination of strength and ductility for 316L material is achieved during L-PBF processing, and additional heat treatments do not improve the material’s performance. Similarly, in [94], it was shown that the heat treatment of L-PBF-fabricated 316L stainless steel resulted in a decrease in the hardness values. In [15], the percentage increase in hardness for the sample made of 300 grade maraging steel, aged at 510 °C for 2 h, was approximately 54.86%.

Elangeswaran et al. [93] investigated the impact of post-treatments on the fatigue performance of 316L stainless steel produced through L-PBF and it was found that the fatigue performance of machined samples, both with and without stress relief heat treatment, surpassed that of conventionally manufactured 316L stainless steel.

For 316L stainless steel, the results from [7] showed that the UTS values are relatively constant for different conditions of hot isostatic pressing (HIP) and solubilization heat treatment, with minor variations within 0–3.5% range. The highest *YS* was achieved in the as-built specimens, while samples subjected to HIP had approximately 45% lower values. On the other hand, the samples subjected only to solubilization heat treatment showed an intermediate behavior, with a yield strength of approximately 370 MPa. The ductility of the as-built samples was noticeably lower, as evidenced by the much lower percentage elongation compared to post-processed specimens. Samples treated with solubilization heat treatment exhibited intermediate behavior in terms of ductility. Additionally, there were significant differences in percentage elongation between samples subjected to HIP post-processing at 50 bar compared to higher pressures. At low pressure (50 bar), the average elongation was 52.7%, while values up to 70% were obtained with high HIP pressure. The hardness of samples subjected to HIP post-processing was 30% lower compared to as-built samples.

The study [90] examined and compared the mechanical properties of 316L steel (fabricated using L-PBF and post-processed through HIP), with wrought 316L steel. The highest hardness values were observed in the as-built samples and those treated at the lowest HIP temperature of 700 °C. The hardness values ranged from 215 to 234 HV, indicating an increase of approximately 29% compared to wrought samples. The elongation increased with temperature, with a maximum increase of 25% compared to the as-built samples. The ultimate UTS shows a gradual decrease with temperature, with the maximum decrease of 9% at 1200 °C. Similarly, the YS decreased with temperature, ranging from 9% at 700 °C to a significant decrease of 50% at 1125 °C.

The effects of different thermochemical post-processing such as high temperature solution nitriding (HTSN) and low temperature nitrocarburizing (LTNC) on the microstructure and properties of additively manufactured 316L austenitic stainless steel were investigated in [22]. For transversally built specimens, the HTSN and also the HTSN + LTNC treatments resulted in decreasing the YS by 45%, the UTS by 14%, and the elasticity modulus by 5%… 6%. Regarding the elongation to failure, an increase was observed of 15% for HTSN and a decrease of 3.84% for the HTSN + LTNC treatment. In the case of vertically built samples, the *YS* decreased by 40%… 44% for HTSN and HTSN + LTNC, respectively. The UTS was smaller only by 3… 5% compared with the as-built samples. The elongation to failure increased by 11% when HTSN treatment was applied but decreased by 7.4 % due to the HTSN + LTNC treatment.

In [99], the test specimens of 316L steel were heated at a temperature of 1040 °C, during 2 h. Both for horizontally and vertically built specimens, due to the heat treatment applied, the *YS* decreased by 18%… 19% and the UTS decreased by 5%. The heat treatment increased (by 7%) the ductility of the horizontally built specimens when compared to the as-built condition. For the vertically built conditions, the heat treatment decreases the ductility by 5%.

After annealing at 1050 °C, following rolling to 25% or 40% strain, the elastic modulus increased by 97% for 25% strain and by 195% for 40% strain, the *YS* decreased by 30% at 0% strain, increased by 9% at 25% strain, and significantly increased by 95% at 40% strain, the UTS decreased by 16% at 0% strain, increased by 13% at 25% strain, and increased by a notable 114% at 40% strain, the elongation to failure increased by 38% at 0% strain, 60% at 25% strain, and a substantial 152% at 40% strain. Overall, these results indicate that annealing at 1050 °C after rolling can greatly enhance the mechanical properties of the samples, making them comparable to conventionally wrought 304 L stainless steel parts [67].

Compared with the as-fabricated L-PBF iron parts, the ultimate tensile strength increased by 12%, the yield strength increased by 37%, and the elastic modulus of the annealed specimens decreased by 9% [3].

The mechanical properties were also determined in [96], for different processing and post-processing factors of 13Cr10Ni1-7Mo2Al0-4Mn0-4Si steel processed by L-PBF. The UTS increased by 50% for the heat-treated machined horizontal specimens compared with the as-built specimens, and by 74% for machined vertical specimens, similar to raw vertical samples. The *YS* of the heat-treated horizontal machined specimens was greater by 43.5% than for the original specimens, and by 50% for vertical machined and raw vertical samples. The heat treatment application resulted in decreasing the elongation to failure by 32% in case of machined horizontal specimens, by 43% for machined vertical samples, and by 39% for raw vertical samples.

After subjecting the 17- 4PH stainless steel to annealing at 550 °C for 4 h, an increase in yield strength was obtained (by 11.8%), ultimate tensile strength (by 4.4%), hardness (by 54.5%), and modulus of elasticity (by 9.37%), but a decrease in elongation to failure (by 15.25%) [34]. However, for specimens annealed at 1040 °C for 1 h and 550 °C for 4 h, the *YS* decreased by 16.33% compared to untreated samples, and the UTS and *E* decreased by 9.73% and 18.12%, respectively. Nevertheless, the elongation to failure and hardness increased by 61% and 59%, respectively.

The effects of aging temperature and aging time on the mechanical behavior of L-PBF maraging 18Ni-300 steel were explored in [20]. For a 500 °C aging temperature, the maximum increase in hardness (57%) was obtained, while the hardness of the material exhibited a significant increase after 1 h of aging (57%), followed by a slight and gradual increase after 3 h of aging (up to 62%). Compared with the as-built model, lower UTS was observed in the under-aging condition at 390 °C (+45%), as well as in the over-aging condition at 590 °C (+27%). The UTS initially increased (+54%), reached a peak value after 3 h of aging (+72%), and subsequently decreased with aging temperature and time.

The effect of different heat treatments on the mechanical behavior of AlSi11Cu alloy obtained by L-PBF was investigated in [82]. As the annealing temperature increased, the strength of the alloy decreased (UTS decreased by 9 h% at 200 °C until 54% at 550 °C, *YS* decreased by 17% at 200 °C until 72% at 550 °C) while its ductility increased (the fracture strain decreased by 50% at 200 °C but after increased rapidly by 40% at 300 °C until 200% at 200 °C). When the alloy was heated at 550 °C and then cooled in water, its properties fell between those obtained from annealing at 300 °C and 400 °C.

Based on the results obtained in [98] for the annealed AlSi3.5Mg2.5 alloy, it can be concluded that the direct-aged specimens (HTS) exhibit significantly higher yield and tensile strengths compared to the annealed specimens (HTD). The percentage difference in yield strength between HTS and HTD was 394.9%, while the percentage difference in tensile strength was 227.2%. This suggests that the aging treatment conducted at 170 °C for HTS resulted in a significant improvement in mechanical properties compared to the annealing treatment at 380 °C for HTD.

The heat treatments post-processing effects on the mechanical properties of Al-7Si-0.6 Mg alloy with added rare earth erbium (Er) were investigated in [102]. The results suggest that the DA treatment (direct aging at 160 °C for 8 h) had a minor negative impact on UTS (−2.5%) and a slight positive impact on *YS* (+4%), but significantly reduced the elongation by 30%. On the other hand, the SR treatment (stress relief annealing at 300 °C for 2 h) had a significant negative impact on UTS and *YS* (40% decrease), but greatly improved the elongation (137% increase). The T6 treatment (solution heat treatment at 540 °C for 1 h followed by quenching in cold water at room temperature, then artificial aging at 160 °C for 8 h) showed a moderate reduction of 10% in UTS and a slight improvement of 6% in *YS*, with a moderate improvement in the elongation (around 40%).

For L-PBF NiTi parts with different porosity levels, solution annealed and aged for 15 min at 350 °C, the elastic modulus dropped from 47 GPa (0% porosity) to 9 GPa (58% porosity)—decreased by 80.85%. The critical stress decreased by 75.49%, from 1224 MPa (0% porosity) to 300 MPa (58% porosity) [16].

The critical stress for plastic deformation was found to increase after aging the L-PBF NiTi samples [17]. After only 30 min of aging at 350 °C, the critical stress was approximately 23.8% higher than for the as-fabricated sample. When the aging time was 1 h, the critical stress further increased, resulting in a percentage difference of approximately 36.3%. Similarly, aging at 600 °C also increased the critical stress for plastic deformation only by 1.1% and after aging for 1.5 h at 600 °C, the critical stress further increased by approximately 8.7% compared to the as-fabricated samples.

**Figure 21:**
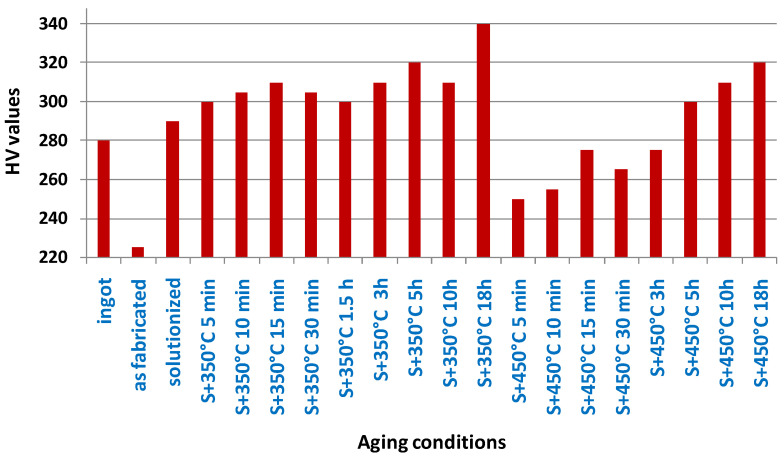
Vickers hardness for different aging conditions.

**Figure 22:**
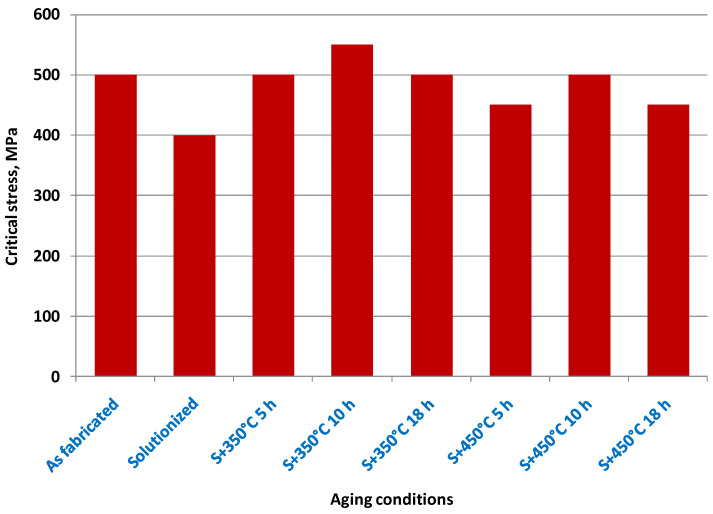
Critical stress for as-fabricated, solution annealed and aged samples.

According to [18], the Vickers hardness value of the as-fabricated NiTi showed a significant decrease compared to the ingot. However, solutioning the as-fabricated sample increased its hardness to a level comparable to the ingot’s hardness. The hardness of the aged samples increased with aging time, and the samples aged at 350 °C exhibited higher hardness compared to those aged at 450 °C, as shown in Figure 21. During compression testing, it was observed that the testing temperature and aging conditions strongly influenced the mechanical behavior of the samples. As-fabricated and solutionized samples did not show superelastic recovery, while partial recovery was observed for the 350 °C aged samples. The recoverable strain increased with aging time, as precipitation hardening increased the critical stress for plastic deformation. In comparison to as-fabricated and solutionized samples, the plastic deformation of aged samples was relatively small, the samples aged at 450 °C presenting higher irreversible strains than those aged at 350 °C. Interestingly, with an increasing aging time, the critical stress for plastic deformation did not show significant changes for aging temperatures (Figure 22).

Based on the experimental results from [8], it can be concluded that the tensile strength increased by 6%, 12.7%, and 17.34%, as the heat treatment temperature increased (from 550 °C, 650 °C, to 750 °C) compared to the as-built titanium samples.

In the experimental work [61], for DED-produced Ti-6Al-4V alloy, annealing treatments at 1050 °C followed by different cooling rates were conducted and the highest hardness was measured in the air cooled and aged specimens (11.5% greater than for the untreated samples).

Compared to the original samples (L-PBF-processed Ti-6Al-2Sn-4Zr-6Mo alloy), the heat-treated specimens showed a gradual increase in hardness, ranging from 2.9% to 7.3%. As the heat treatment temperature increased from 600 °C to 950 °C, the *YS* increased by 90% to 180%, depending on the specific temperature. The UTS was similar to the original sample, except for the case of heat treatment at 600 °C, when it was 25% greater. However, the elongation to failure was reduced in the heat-treated samples, ranging from 20% to 72% for the different temperatures tested [84].

**Figure 23:**
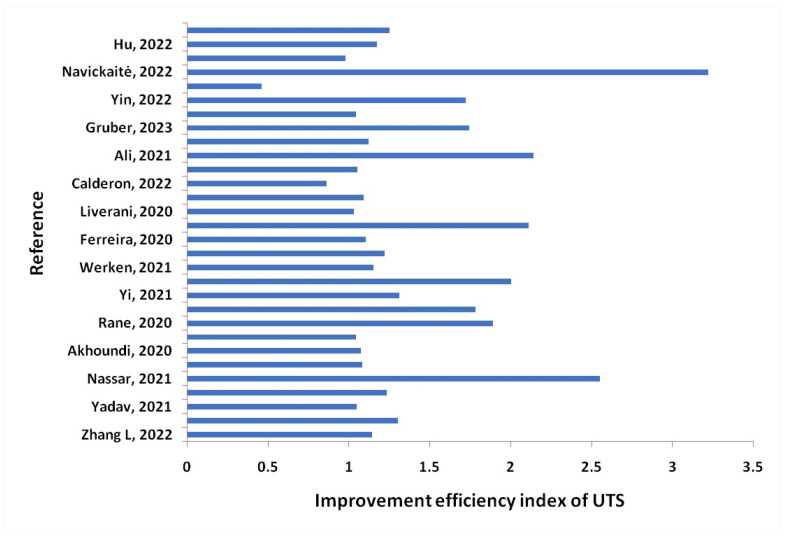
The distribution of improvement efficiency index in different reference papers [3,4,7,8,20,22,26,31,32,33,34,38,41,43,47,48,50,52,53,54,56,67,71,82,84,90,96,98,99,102].

**Figure 24:**
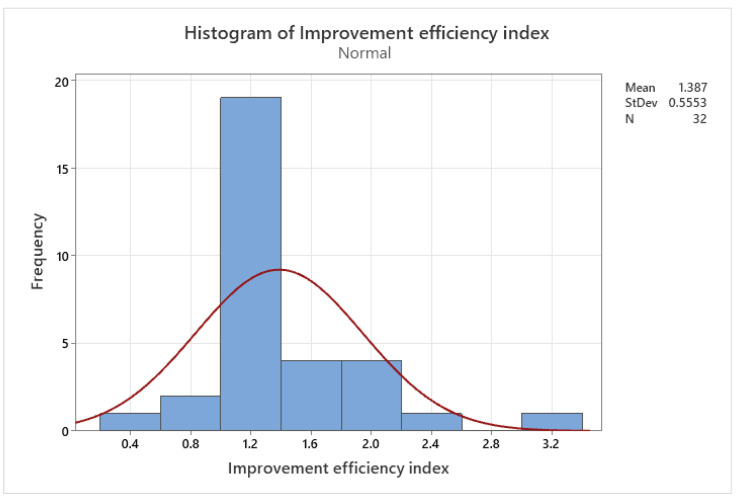
Histogram of improvement efficiency index.

Hence, the majority of the references have assessed tensile properties, so the authors have chosen UTS as the input for normalization analysis. For this analysis, the improvement efficiency index (defined as the ratio of the mechanical property of the printed parts obtained after different post-processing treatments and the value corresponding to the as-build samples) was used, as seen in Figure 23 and Figure 24.

It can be observed that the most frequent improvement efficiency index has a value of 1.2, meaning that the majority of references studied showed an mean increase in UTS of 20% after applying post-processing treatments. The non-uniform Gaussian distribution can be explained by the variety of different factors such as: materials used, printing technology, printing parameters (building orientation, infill percentage, layer height), post-processing treatments (temperature of treatment, maintaining time, cooling conditions, etc.).

The post-processing heat treatments can significantly affect the mechanical properties of 3D-printed parts, including tensile strength, elongation at break, yield strain, and total energy absorption. The results may vary depending on the material, annealing temperature, and time duration. In general, annealing tends to improve the tensile strength and elastic modulus of 3D-printed parts, leading to increased structural integrity and stiffness. However, the effect on elongation at break and yield strain may vary, with some materials showing increased ductility while others may experience decreased deformability due to the development of a crystalline structure.

The infill density and layer thickness of 3D-printed parts can also influence their mechanical properties. Higher infill density and smaller layer thickness tend to result in higher tensile strength, while lower infill density may lead to increased porosity and reduced strength.

The addition of certain materials, such as EVA or carbon fibers, can also affect the mechanical properties of 3D-printed parts. EVA may decrease tensile strength but increase elongation at break, while carbon fibers can significantly increase the elastic modulus of the parts.

The relationship between post-processing treatments, such as annealing, and mechanical properties of 3D-printed parts may not always be linear, and optimal conditions for improving mechanical properties may vary depending on the specific material and printing parameters used.

## 6. Conclusions

This paper presents an extensive review of research studies that focus on enhancing the mechanical properties of parts manufactured with different 3D printing technologies, described in detail. The 3D printing materials with their mechanical characteristics are also presented. Complex heat treatments are critical processes used to improve the properties of additive manufactured plastic and metal parts. Various process improvement methods such as annealing, tempering, and precipitation hardening, or complex combinations of these heat treatments are investigated and analyzed, the main post-processing parameters are summarized and described, in the function of materials and 3D printing technology used, based on 100 representative papers in the field. These processes can improve strength, durability, surface finish, dimensional stability, and achieve specific properties required for different applications. By carefully controlling the heat treatment process parameters, the properties of additive manufactured parts can be optimized to meet the requirements of the applications for which they are intended. It is important to carefully consider the post-processing treatments and their effects on mechanical properties when designing 3D-printed parts for specific applications. Post-processing treatments can be used strategically to modify the mechanical properties of 3D-printed parts in order to meet desired performance requirements. However, thorough testing and evaluation of the mechanical properties under different conditions are necessary to ensure the reliability and performance of the final printed parts. Further research and experimentation are needed to better understand the effects of post-processing heat treatment on tensile properties and to develop optimized heat treatment strategies for specific 3D printing materials and applications. By comparing the efficiency of improvement and the achieved mechanical properties of 3D-printed parts using different technologies and post-processing treatments, this paper provides guidance for evaluating the impact of these methods in fabricating high-performance parts for various applications.

## Figures and Tables

**Table 1:** The materials used for additive technology.

Category	Examples
Polymers	Acrylonitrile Butadiene Styrene (ABS), Polylactic Acid (PLA), Polyethylene Terephthalate Glycol (PETG), Nylon
Metals	Stainless Steel, Titanium, Aluminum, Copper
Ceramics	Zirconia, Alumina, Silicon Carbide
Composites	Carbon Fiber, Glass Fiber, Kevlar
Biomaterials	Collagen, Chitosan, Alginate

**Table 2:** Some mechanical characteristics of 3D materials used for additive technology.

Category	Tensile Strength (MPa)	Elastic Modulus (GPa)	Density (g/cm³)	Typical Uses	Ref.
Polymers Polymers are the most commonly used 3D printing materials due to their low cost and ease of use. However, their mechanical properties can vary greatly depending on the specific material and printing parameters used. Generally, polymers have lower tensile strength and stiffness compared to metals and ceramics, but they can still be used for a wide range of applications, including prototyping, consumer products, and packaging.	25–70	1–5	0.9–1.4	Consumer products, toys, prototyping, packaging	[38,42,43,52,71]
Metals Metal 3D printing is a rapidly growing field due to the high strength and durability of metal parts. However, metal 3D printing is generally more expensive and complex than polymer printing. Metal parts can be printed with a range of tensile strengths, from around 400 MPa for aluminum to over 1200 MPa for titanium. Metal parts also tend to have higher elastic moduli and densities compared to polymers.	400–1200	100–200	6–19	Aerospace, medical implants, automotive parts	[2,4,5,6,7,8,9,10,11,12,13,14]
Ceramics Ceramic 3D printing is used for applications that require high temperature resistance, chemical resistance, and wear resistance. Ceramic parts can have tensile strengths ranging from 100 to 400 MPa, and elastic moduli ranging from 100 to 400 GPa. Ceramic parts also tend to have low densities, which makes them useful for aerospace and other weight-sensitive applications.	100–400	100–400	2.5–7.5	Dental implants, aerospace, high-temperature parts	[16,27]
Composites Composite materials are made by combining two or more materials to create a new material with enhanced properties. Composites can have tensile strengths ranging from 500 to 2000 MPa, and elastic moduli ranging from 50 to 100 GPa. Composites are used in a wide range of applications, including aerospace, automotive, and sports equipment.	500–2000	50–100	1.5–2.5	Aerospace, automotive, sports equipment	[26,41,49,52,53]
Biomaterials Biomaterials are used in 3D printing to create tissue and organ replacements, as well as drug delivery systems. Biomaterials tend to have lower mechanical properties compared to other 3D printing materials, with tensile strengths typically in the range of 1–5 MPa, and elastic moduli in the range of 0.01–0.1 GPa. However, biomaterials have unique properties that make them useful for medical applications, such as biocompatibility and bioresorbability.	1–5	0.01–0.1	1–1.5	Tissue engineering, drug delivery	[1]

**Table 3:** Some mechanical characteristics of PLA.

Property	Value
Tensile Strength PLA’s tensile strength typically ranges from 25 to 70 MPa. This is lower than some other 3D printing materials such as ABS and nylon, which can have tensile strengths exceeding 100 MPa. However, PLA can be stiffened by increasing its density through annealing or other post-processing techniques.	25–70 MPa
Young’s Modulus PLA’s Young’s modulus, a measure of its stiffness, typically ranges from 2.7 to 4 GPa. This is higher than some other 3D printing materials such as TPU, but lower than materials such as carbon fiber-reinforced polymers.	2.7–4 GPa
Elongation at Break PLA’s elongation at break, a measure of its ability to stretch before breaking, is typically between 2 and 10%. This is lower than some other materials such as TPU, which can have elongations at break exceeding 500%.	2–10%
Flexural Strength PLA’s flexural strength, a measure of its ability to resist bending, typically ranges from 50 to 100 MPa. This is lower than some other materials such as polycarbonate, which can have flexural strengths exceeding 150 MPa.	50–100 MPa
Density PLA has a density of 1.24–1.27 g/cm^3^, which is similar to or slightly lower than other common 3D printing materials such as ABS and PETG.	1.24–1.27 g/cm^3^
Glass Transition Temp PLA’s glass transition temperature, the temperature at which it transitions from a hard, glassy state to a soft, rubbery state, is typically around 55–60 °C. This can make it unsuitable for use in high-temperature applications.	55–60 °C
Melting Temperature PLA’s melting temperature can vary depending on the specific grade and manufacturer, but is typically between 160 and 220 °C. This relatively low melting temperature, combined with PLA’s low tendency to warp and its ability to produce high-detail prints, makes it a popular material for use in desktop 3D printers.	160–220 °C (depending on grade)

**Table 4:** Some mechanical characteristics of ABS.

Property	Value
Tensile Strength ABS typically has a tensile strength of 40–50 MPa, making it stronger than PLA but weaker than materials such as nylon and polycarbonate.	40–50 MPa
Young’s Modulus ABS has a Young’s modulus of around 2 GPa, making it stiffer than materials such as TPU but less stiff than materials such as polycarbonate.	2 GPa
Elongation at Break ABS typically has an elongation at break of 10–50%, making it more flexible than PLA but less flexible than materials such as TPU.	10–50%
Flexural Strength ABS typically has a flexural strength of 60–100 MPa, making it stronger than PLA but weaker than materials such as polycarbonate.	60–100 MPa
Density ABS has a density of around 1.04–1.05 g/cm^3^, making it slightly less dense than PLA.	1.04–1.05 g/cm^3^
Glass Transition Temperature ABS has a glass transition temperature of around 100 °C, making it more heat-resistant than PLA.	100 °C
Melting Temperature ABS has a melting temperature of around 210–250 °C, making it similar to PLA in terms of print temperature.	210–250 °C

**Table 5:** Some mechanical characteristics of PEEK.

Property	Value
Tensile Strength	90–100 MPa (13,000–15,000 psi)
Young’s Modulus	3.6–4.1 GPa (522,000–594,000 psi)
Elongation at Break	20–50%
Flexural Strength	140–160 MPa (20,000–23,000 psi)
Compressive Strength	120–150 MPa (17,000–22,000 psi)
Hardness	88–90 Shore D
High thermal stability: PEEK can withstand continuous exposure to temperatures up to 250 °C (482 °F), with short-term exposure up to 310 °C (590 °F). This makes it suitable for use in high-temperature applications where other materials would break down.	
Chemical resistance: PEEK is highly resistant to a wide range of chemicals, including acids, bases, and organic solvents. This makes it ideal for use in harsh environments where exposure to chemicals is a concern.	
Biocompatibility: PEEK is biocompatible and has been used in medical implants, such as spinal implants and dental implants. Its biocompatibility makes it an attractive material for medical applications.	
Electrical properties: PEEK has excellent electrical insulation properties, making it suitable for use in electrical and electronic applications.	

**Table 6:** Some mechanical characteristics PLA.

Mechanical Property	Nylon 6	Nylon 12
Tensile strength	50–80 MPa	40–50 MPa
Nylon has a high tensile strength and high Young’s modulus, which means it can withstand a lot of stress without breaking or deforming. However, it can be prone to warping and shrinking during the printing process, especially if the printer is not well-calibrated or the part design is not optimized for 3D printing.	7250–11,600 psi	5800–7250 psi
Young’s modulus	2.5–3.5 GPa	1.2–2.8 GPa
	360,000–507,500 psi	174,000–406,000 psi
Elongation at break Nylon is also known for its high elongation at break, which means it can stretch quite a bit before breaking. This property can be useful for creating parts that need to flex or bend, such as hinges or springs.	100–200%	200–300%
Flexural strength	80–120 MPa	60–70 MPa
In addition to its high tensile strength, nylon also has good flexural and compressive strength. This makes it a good choice for parts that need to support weight or resist deformation under load.	11,600–17,400 psi	8700–10,150 psi
Compressive strength	60–80 MPa	50–70 MPa
	8700–11,600 psi	7250–10,150 psi
Hardness Nylon is a relatively hard material, with a high Shore D hardness rating. This means it can resist scratching and other surface damage but may also make it more difficult to print fine details or intricate shapes.	80–110 Shore D	70–80 Shore D

**Table 7:** Some mechanical characteristics of PETG.

Mechanical Property	Value
Tensile strength PETG has a relatively high tensile strength for a 3D printing material, making it strong and durable.	50–70 MPa (7250–10,150 psi)
Young’s modulus PETG has a relatively low Young’s modulus compared to some other 3D printing materials, such as PLA or ABS. This means that PETG is more flexible and less brittle, making it less likely to break under stress.	2.0–3.0 GPa (290,000–435,000 psi)
Elongation at break PETG has a relatively high elongation at break, indicating that it can stretch and bend without breaking.	70–130%
Flexural strength PETG has a relatively high flexural strength, making it resistant to bending or flexing.	80–90 MPa (11,600–13,050 psi)
Compressive strength PETG has a relatively high compressive strength, making it able to withstand heavy loads.	60–80 MPa (8700–11,600 psi)
Hardness PETG has a moderate hardness, making it durable and resistant to wear and tear.	68–72 Shore D

**Table 8:** Printing parameters for the heat treatment [37].

Material	Extrusion Temperature (°C)	Speed (mm/s)	Layer Height (mm)	Infill (%)	
PETG	265	20	0.4	100	
PETG CF	195	60	0.52	100	
PETG KV	265	20	0.35	100	
**Samples Group**	**Temperature (°C)**	**Time (min)**	**Samples Group**	**Temperature (°C)**	**Time (min)**
1	90	30	2	90	240
3	90	480	4	110	30
5	110	240	6	110	480
7	130	30	8	130	240
9	130	480			

**Table 9:** Mechanical properties of PETG and CFPETG sample [38].

Material/Post Process	Infill Density	Hardness (HRC)	Tensile Strength (MPa)	Impact Strength (J/m^2^)	Flexural Strength (MPa)
Annealed PETG	25%	69	38	68	51
	50%	77	41	74	59
	75%	90	47	88	65
	100%	97	52.4	96	70
Annealed CFPETG	25%	93	53	89	67
	50%	106	59	101	71
	75%	119	64	113	78
	100%	124	70.3	122	84

**Table 10:** The 3D printing parameters for MAX-G PETG, CARBONX CFR-PETG, ECOMAX PLA, CARBONX CFR-PLA [39].

Parameter	Value	Parameter	Value
Nozzle Diameter	0.8 mm	Extruder Temperature	250 °C (PETG and PETG-CF)
			210 °C (PLA and PLA-CF)
Retraction distance	1.5 mm	Bed Temperature	90 °C (PETG and PETG-CF) 55 °C (PLA and PLA-CF)
Retraction speed	60 mm/s	Printing speed	30 mm/s
Layer height	0.36 mm	Printing speed for first layer	15 mm/s
Outline perimeters/shells	2	Movement speed	60 mm/s
Top/ bottom solid layers	0	Layer cooling fan	Off
Infill	None		

**Table 11:** 3D printing parameters [31].

Parameter	Value	Parameter	Value	
Speed	60 mm/s	Layer thickness	0.2 mm	
Bed temperature	60 °C		0.3 mm	
Number of shells	3		0.4 mm	
Air gap	0			
Infill pattern	Rectilinear	Annealing Temperature	80 °C	120 °C
Nozzle temperature	210 °C	Infill density	60%	90%

**Table 12:** The 3D printing parameters for PLA Ingeo and PHB [42].

Parameter	Value	Parameter	Value
Nozzle diameter (mm)	0.35	Layer thickness (mm)	0.15
Printing bed material	Kapton	Shell thickness (mm)	1.05
Printing bed temperature, Tbed (°C)	55	Infill rate (%)	100
Nozzle temperature, Nozzle (°C)	190—PLA with PHB, 210—PLA	Strand orientation (◦)	45
Printing speed (mm s^−1^)	50		

**Table 13:** Additive printing setting [46].

Materials	Drying Protocol	Annealing Conditions (For Pellets and Part)
Neat PPS	120 °C, 3 h (Vacuum)	250 °C, 18 h, Air 250 °C
PPS 40CF	130 °C, 4 h (Vacuum)	18 h, Air
PPS 50CF	130 °C, 4 h (Vacuum)	250 °C, 18 h, Air
PPS 60CF	130 °C, 4 h (Vacuum)	250 °C, 18 h, Air

**Table 14:** Annealing treatment parameters [52].

Post fabrication Treatment	Parameters
Annealing (AN)	150 °C for 4 h 170 °C for 4 h 200 °C for 30 min
Annealing with Compaction (AN-C)	150 °C for 4 h at 0.55 MPa 170 °C for 4 h at 0.55 MPa Step 1: 200 °C for 30 min at 0.55 MPa Step 2: 150 °C for 2 h (without pressure)

**Table 15:** Victrex PEEK 151 and Victrex AM 200 mechanical properties [47].

Material	Tm (°C)	Tg (°C)	Shear Viscosity (Pa.s)	Filament Diameter (mm)
Victrex PEEK 151	346	140	130	1.75
Victrex AM 200	304	156	250	1.75

**Table 16:** The printing parameters and the annealing temperatures [47].

Material	Printing Bed °C	Printing Nozzle °C	Printing Chamber °C	Build Orientation	Annealing Temperature		Time h
					LAT *	HAT **	
Victrex PEEK151	100	410	60	Flat, Side, Vertical	156	200	2, 4
Victrex AM 200	100	380	60	Flat, Side, Vertical	170	200	2, 4

* LAT—low annealing temperature; ** HAT—high annealing temperature.

**Table 17:** Mechanical properties for acrylonitrile butadiene styrene 9% concentration of carbon fibers (ABSCF)/polylactide with 9% concentration of carbon fibers (PLACF) [26].

Filament Type	Density g/cc	Tensile Strength (MPa)	Tensile Modulus (MPa)	Tensile Elongation (%)	Flexural Strength (MPa)	Extrusion Temp	Glass Transition Temperature
PLACF	1.29	48	4950	2	89	215 °C	60 °C
ABSCF	1.11	46	5210	2	76	230 °C	105 °C

**Table 18:** FX256 and CF15 mechanical properties [43].

Property	CF15	FX256
Material density (g/cm^3^)	1.08	1.01
Melt flow index (g/10 min)	9.92	95
Tensile strength (MPa)	54.50	45.00
Tensile modulus (MPa)	500	1400
Melting temperature (8 °C)	160	178
Print temperature (8 °C)	235–260	235–260

**Table 19:** Printing parameters used [43].

Nozzle Diameter	0.4 mm
Nozzle material	brass/A2 steel
Layer height	0.3 mm
Number of perimeters per layer	2
Infill percentage	100%
Infill pattern	Rectilinear 645
Skirt outlines	7
Extrusion temperature	260 °C
Bed temperature	90 °C
Bed surface	Tempered glass
Printing speeds	40 mm/s
Extrusion multiplier	1.04

**Table 20:** Mechanical properties and printing parameters used [58].

Parameter	Value
Density	1.27 g/cm^3^
Molecular formula	C37H24O6N2
Glass transition temperature (Tg)	215 °C
Extrusion tip temperature	400 °C
Max. build platform temperature	190 °C
Layer thickness	330 µm
Infill density	100%
Raster angle	0/90

**Table 21:** A short summary of different post-processing of the heat treatment impact on additive manufacturing: L-PBF (laser powder bed fusion); L-PBF (laser beam bed fusion); DMD (direct melting deposition); EBM (electronic beam melting); ME (fused deposition melting); DMLS (direct melting laser sintering); LMD (laser melting deposition); PBF (powder bed fusion), WAAM (Wire + arc additive manufacturing); and ME (material extrusion) [66].

Materials	Additive Manufacturing Technology	Post- Processing	Investigated Properties
Nickel-based superalloy	L-PBF	Stress-relief heat treatment	Microstructural evolution Elemental segregation Ubiquitous to AM process
Ti–6Al–4V TiB/Ti–6Al–4V composite	L-PBF	Annealing heat treatment	Microstructures’ evolution Mechanical properties
Inconel 718	L-PBF	Annealing heat treatment	Dislocation structures Chemical segregation and Precipitates/Laves phase
Ti6Al4V	L-PBF	Hot isostatic pressing	Building orientation Mechanical properties Microstructures
AlSi10Mg	L-PBF	Solution heat-treated Annealing process	Microstructures Mechanical properties
Ti2AlNb	L-PBF	Solution-treated Water quenching Aging-treated	Microstructure Mechanical Properties
IN738LC superalloy	L-PBF	Solid-solution artificial aging treatment	Microstructure evolution High temperature oxidation
Titanium Alloy	L-PBF	Annealing heat treatment	Microstructural Mechanical Properties
AlSi10Mg	L-PBF	Annealing heat treatment	Thermal conductivity Porosity measurement Microstructure
AlSi10Mg	L-PBF	Annealing heat treatment	Mechanical properties
Low-carbon steel Austenitic-stainless steel	WAAM	Annealing heat treatment Water quenching	Microstructure Mechanical behavior
Ti6Al4V	L-PBF	Hot isostatic pressing Annealing heat treatment	Microstructure Mechanical properties
Polymer	ME	Temperature change hot chamber	Thermal performance
Ti6Al4V	EBM	Super β transus treatment	Compressive strength
Ti-6A1–4V	EBM	Vacuum heat treatment	Mechanical properties Microstructure
Ti-6Al-4V	LMD	Vacuum solution and aging heat treatment	Fatigue crack growth

**Table 22:** Chemical composition for FeCoCrNi HEA powder (σ_0.2_ = 600 MPa, σ_u_ = 745 MPa) [2].

Element%	Fe	Co	Ni	Cr	Si	C
FeCoCrNi HEA powder	24.47	24.66	24.47	26.24	0.12	0.06

**Table 23:** HEA system [1].

HEAs Systems	Additive Technology	Phase Compositions (Detailed HEA Compositions)	
AlCoCrCuFeNi	L-PBF	FCC + BCC (AlCoCrCuFeNi)	
	LMD	BCC + B2 (Al1.5CoCrCuFeNi)	
		FCC + BCC (AlCoCrCuFeNi)	
AlCoCrCuFeNiTi	LMD	BCC + L21 (AlCo0.5CrCu0.5FeNi1.5Ti0.4)	
AlCoCrCuFeNi + WC	L-PBF	FCC + WC + W2C + g-carbide (20% AlCoCrCuFeNi + 80% WC)	
AlCoCrFeMnNi	L-PBF	FCC (AlCo0.9CrFeMn0.9Ni), FCC + BCC + B2 + Oxides (AlCoCrFeMnNi)	
	LMD	FCC (Al0.1CoCrFeMnNi), FCC + BCC (Al0.26CoCrFeMnNi, Al0.43CoCrFeMnNi)	
AlCoCrFeMoNiW	L-PBF	FCC + B2 (Al18Co30Cr10Mo1Ni30W1)	
AlCoCrFeNi	L-PBF	FCC (Al0.1CoCrFeNi)	
		FCC (Al0.3CoCrFeNi)	
		FCC (Al0.5CoCrFeNi)	
		FCC + BCC (Al0.5CoCrFeNi)	
		BCC (AlCoCrFeNi)	
		BCC + B2 (AlCoCrFeNi)	
		A2 + B2 (AlCoCrFeNi)	
		BCC + Oxides (AlCoCr1.3FeNi1.3)	
	LMD	FCC (Al0.3CoCrFeNi), FCC + BCC (Al0.6CoCrFeNi), BCC (Al0.85CoCrFeNi)	
		FCC (Al0.3CoCrFeNi), FCC + BCC + B2 (Al0.6CoCrFeNi), BCC + B2 (Al0.85CoCrFeNi)	
		FCC (Al0.2CoCrFeNi), FCC + BCC (Al0.45CoCrFeNi, Al0.7CoCrFeNi), BCC + B2 (AlCoCrFeNi)	
		FCC (Al0.3CoCrFeNi1.7), FCC + B2 (Al0.7CoCrFeNi1.3), A2 + B2 + L12 (AlCoCrFeNi), A2 + B2 (Al1.7CoCrFeNi0.3)	
		FCC (Al0.3CoCrFeNi), FCC + BCC (Al0.7CoCrFeNi)	
		FCC (Al0.3CoCrFeNi)	
		FCC + B2 (Al0.3CoCrFeNi)	
		FCC + B2 (AlxCoCrFeNi2.1, 0.6 x 1.1) BCC (AlCoCrFeNi)	
		BCC + B2 (AlCoCrFeNi)	
		BCC + B2 (AlCoxCr1-xFeNi, 0 < x < 1)	
		FCC + B2 (AlCoCrFeNi1.2)	
		FCC + BCC (AlCoCrFeNi2.1)	
		FCC + BCC (AlCo2.2CrFeNi, AlCo2.8CrFeNi)	
		FCC + B2 (Al18Co30Cr10Fe10Ni32)	
		FCC + L12 (Al7.1Co14.2Cr14.2Fe14.2Ni50), FCC + L12 + BCC (Al12.5Co12.5Cr12.5Fe12.5Ni50,	
		Al16.7Co1.1Cr11.1Fe11.1Ni50)	
	EBM	FCC + BCC + B2 (AlCoCrFeNi)	
	WAAM	FCC + BCC (Al2Co1.8Cr0.3Fe2.7Ni3.2)	
		A2 + B2 (Al2.1Co0.3Cr0.5FeNi2.1)	
		Al3Ni+(Ni, Co)3Al4 (Al2.1Co0.3Cr0.5FeNi2.1)	
		AlNi + CrFe + Al2FeCo (Al36.5Co4.9Cr8.6Fe16.4Ni33.7)	
AlCoCrFeNi/CoCrFeNi laminated structure	LMD	FCC + BCC (AlCoCrFeNi + CoCrFeNi)	
AlCoCrFeNiTi	L-PBF	FCC (AlCoCrFeNiTi), FCC + BCC + B2 (AlCo0.8CrFeNiTi)	
	LMD	FCC + Oxides (Al0.2Co1.5CrFeNi1.5Ti0.3)	
		A2 + B2 (AlCoCrFeNiTi0.5)	
		FCC + BCC + AlNi3 intermetallic (AlCoCrFeNiTi)	
		FCC (Al4(CoCrFeNi)94Ti2)	
		(AlCoCrFeNiTi)	
AlCoCrFeNiTi/CoCrFeMnNi laminated structure	LMD	FCC + BCC (AlCoCrFeNiTi0.5 + CoCrFeMnNi)	
AlCoCrFeNiV	L-PBF	FCC (Al0.5CoCr0.8FeNi2.5V0.2)	
AlCoCuFeNi	L-PBF	BCC (AlCoCuFeNi)	
		BCC + B2 (AlCoCuFeNi)	
	LMD	FCC (Al0.25CoCu0.75FeNi, Al0.5CoCu0.5FeNi), FCC + BCC (Al0.75CoCu0.25FeNi)	
AlCoFeMnNi	L-PBF	FCC (Al0.26CoFeMnNi)	
AlCoFeMnNi + C	L-PBF	(Al0.26C0.12CoFeMnNi)	
AlCoFeNi AlCoFeNiSmTiV	LMD L-PBF	BCC + B2 (AlCoxCr1-xFeNi, 0 x 1) FCC (AlCoFeNiSm0.1TiV0.9)	
AlCoFeNiSmTiVZr	L-PBF	Several intermetallic such as Al-Sm, Al3V, Al3Zr, (Fe, Al)2Zr (AlCoFeNiSm0.05TiV0.95Zr)	
AlCoFeNiSmV	L-PBF	FCC (AlCoFeNiSm0.1V0.9)	
AlCoFeNiTi	L-PBF	Al7(CoFeNi)86Ti7	
AlCoFeNiTiVZr	L-PBF	FCC (AlCoFeNiTiVZr)	
AlCrCuFeNbNi	LMD	FCC + BCC (AlCrCuFeNbxNi, x = 0.05, 0.16, 0.26)	
AlCrCuFeNi	L-PBF	BCC + B2 (AlCrCuFeNi)	
		FCC (Al0.5CrCuFeNi2), FCC + BCC + B2 (Al0.75CrCuFeNi2, Al1.0CrCuFeNi2)	
		FCC + BCC + B2 (AlCrCuFeNix, x = 2.0, 2.5, 2.75, 3.0, 3.5)	
	LMD	FCC + L12 + BCC + B2 (Al0.8CrCuFeNi2, Al1.0CrCuFeNi2), FCC + BCC + B2 (Al1.3CrCuFeNi2, Al1.5CrCuFeNi2)	
		FCC + BCC (AlCrCuFeNi)	
AlCrCuFeNiW	LMD	FCC + BCC (AlCrCuFeNiWx, x = 1% and 3%)	
AlCrFeMoV	LMD	BCC (AlCrFeMoVx, 0 < x < 1)	
AlCrFeNi	L-PBF	FCC + BCC + B2 (AlCrFe2Ni2)	
	LMD	BCC + B2 (AlCrFeNi)	
		FCC + BCC + B2 (AlCrFe2Ni2)	
lCrFeNiV	L-PBF	FCC + L12 (Al0.5Cr0.9FeNi2.5V0.2)	
		(Al0.5CrFeNi2.5V0.2)	
AlCrMoNbTa	EBM	BCC (Al0.5CrMoNbTa0.5)	
		BCC + Cr2Nb intermetallic phases (Al0.5CrMoNbTa0.5)	
CoCrCuFeNi	LMD	(CoCrCu0.5FeNi)	
CoCrCuFeMnSi	L-PBF	FCC + HCP (Co20Cr15Cu1.5Fe38.5Mn20Si5)	
CoCrFeMn	L-PBF	FCC + HCP (Co10Cr10Fe50Mn30)	
	LMD	FCC + HCP (Co10Cr10Fe50Mn30)	
CoCrFeMn + C	L-PBF	FCC (Co10Cr10Fe49.5Mn30C0.5)	
	LMD	FCC (Co10Cr10Fe49.5Mn30C0.5)	
CoCrFeMnNbNi	LMD	((CoCrFeMnNi)1-xNbx, 0 < x < 0.3)	
CoCrFeMnNi	L-PBF	FCC (CoCrFeMnNi)	
		FCC + r (CoCrFeMnNi)	
		FCC + HCP (CoCrFeMnNi)	
		FCC + Oxides (CoCrFeMnNi)	
		FCC + x (CoCr1.3FeMnNi0.7)	
		(CoCrFeMnNi)	
	LMD	FCC (CoCrFeMnNi)	
		FCC + BCC (CoCrFeMnNi)	
		FCC ((CoCrFeMnNi)1-xFex, x = 0.1, 0.2, 0.3, 0.4, 0.5), FCC + BCC ((CoCrFeMnNi)0.4Fe0.6)	
		(CoCrFeMnNi)	
	EBM	FCC (CoCrFeMnNi)	
CoCrFeMnNi + C	L-PBF	FCC (CoCrFeMnNi + 1% C)	
		FCC (CoCrFeMnNi + 0.2% C)	
		FCC ((CoCrFeMnNi)100-xCx, x = 0.5, 1.0, 1.5)	
CoCrFeMnNi + CeO2	LMD	FCC + Oxides (CoCrFeMnNi + 1% CeO2)	
CoCrFeMnNi + Fe-based metallic glass	L-PBF	FCC + amorphous phase (CoCrFeMnNi + x% (Fe43.7Co7.3Cr14.7Mo12.6C15.5B4.3Y1.9), x = 5, 10, 20, 30)	
CoCrFeMnNi + N	L-PBF	FCC (CoCrFeMnNi + 50% N2 mixed with Ar)	
CoCrFeMnNi-(N, Si)	L-PBF	FCC + amorphous phase (Co20.26Cr19.43Fe21.69Mn16.83Ni20.45N0.92Si0.42)	
CoCrFeMnNiTa	LMD	((CoCrFeMnNi)1-xTax, 0 < x < 0.9)	
CoCrFeMnNiTi	EBM	FCC + CrFe + Cr2Ti + Ni3Ti (CoCrFeMn0.18NiTi), BCC + CrFe + Cr2Ti + Ni3Ti (CoCrFeMn0.5NiTi, CoCrFeMn2NiTi)	
CoCrFeMnNi + TiAlV	LMD	((CoCrFeMnNi)1-x(TiAl6V4)x, 0 < x < 1)	
CoCrFeMnNi + TiB2	LMD	FCC + TiB2 (CoCrFeMnNi + 5% TiB2)	
CoCrFeMnNi + TiC	L-PBF	FCC + TiC (CoCrFeMnNi + 1% TiC)	
	LMD	FCC + TiC (CoCrFeMnNi + x% TiC, x = 2.5, 5)	
CoCrFeMnNi + TiN	L-PBF	FCC + TiN (CoCrFeMnNi + 5% TiN)	
		FCC + TiN (CoCrFeMnNi + 12% TiN)	
CoCrFeMnNi + WC	LMD	FCC + M23C6 (CoCrFeMnNi + x% WC, x = 5, 10)	
CoCrFeMnSi	L-PBF	FCC + HCP (Co20Cr15Fe40Mn20Si5)	
		FCC + HCP ((Co10Cr10Fe50Mn30)100-xSix, x = 1, 3, 5)	
CoCrFeMoNi	LMD	FCC (CoCrFeMo0.2Ni)	
CoCrFeMoNiTi	L-PBF	FCC + HCP (Co1.5CrFeMo0.1Ni1.5Ti0.5)	
		FCC (Co27Cr16Fe18Mo28Ni8Ti3)	
	EBM	FCC + Ni3Ti (Co1.5CrFeMo0.1Ni1.5Ti0.5)	
CoCrFeNbNi	LMD	FCC + Laves (CoCrFeNbxNi, x = 0.1, 0.15, 0.2)	
		FCC + Laves + Cubic Nb-rich phase (CoCrFe Nb0.2Ni2.1)	
CoCrFeNi	L-PBF	FCC (CoCrFeNi)	
		FCC (CoCr0.5FeNi)	
		FCC + BCC + d (Co23Cr21Fe21Ni35)	
		FCC (CoCrFeNi3)	
		FCC (Co15Cr10Fe60Ni15)	
		(CoCrFeNi)	
	LMD	FCC (CoCrFeNi)	
		FCC (Cox1Crx2Fex3Nix4, x1 = 7~44, x2 = 5~32, x3 = 5~45, x4 = 19~58)	
CoCrFeNi + Al alloy	LMD	(12 % CoCrFeNi + AA5083 composite)	
CoCrFeNi + C	L-PBF	FCC (CoCrFeNiC0.05)	
		FCC + M23C6 (CoCrFeNiC0.05)	
CoCrFeNi + N	L-PBF	FCC (CoCrFeNi + 1.8% N)	
CoCrFeNi + Diamond	L-PBF	FCC + Diamond + Cr7C3 (CoCrFeNi / diamond composite)	
CoCrFeNi + Ti-coated diamond	L-PBF	FCC + Diamond (CoCrFeNi / Ti-coated diamond composite)	
CoCrFeNiSi	L-PBF	FCC (CoCrFeNiSi0.05)	
CoCrFeNiTiW	L-PBF	BCC (CoCr2.5FeNi2TiW0.5)	
		BCC + TiN (CoCr2.5FeNi2TiW0.5, under N2 protective gas)	
CoCrFeNiW	L-PBF	FCC + W (CoCrFeNiW0.2)	
CoFeNiTi	LMD	FCC ((CoFeNi)100-xTix, x = 0, 1, 2, 3, 4)	
CrCuFeNi	L-PBF	FCC (CrCuFeNi2)	
CrCuFeTiV	LMD	FCC + BCC (CrCuFeTiV)	
CrFeMnNi	L-PBF	FCC (CrFeMnNi)	
CrFeMoNiTi	LMD	FCC + r + Ni3Ti + Ti nitride ((CrFeNi)90Mo5Ti5), r + C14 Laves + Chi phase + Ni3Ti + TiN ((CrFeNi)80Mo10Ti10),	
CrFeNiTiVZr	LMD	FCC + r + C14 Laves + Ni3Ti + TiN ((CrFeNi)80Mo15Ti5)	
		C14 Laves + aTi-rich phase (CrFeNiTiVZr)	
CrFeNiTiW	L-PBF	FCC (Cr4Fe9Ni5TiW)	
		FCC + Unknown phase (Cr4Fe9Ni6TiW)	
CuNbNiTiZrFeLaMnNiV	LMD	((CuNbTiZr)65Ni35)	
		r + La(Ni,Mn)5 (Fe0.2La0.03Mn0.4Ni0.17V0.2, Fe0.3 La0.03Mn0.2Ni0.17V0.3, Fe0.16La0.06Mn0.33Ni0.28V0.16, Fe0.2La0.07Mn0.2Ni0.33V0.2), FCC + La(Ni,Mn)5 (Fe0.1La0.07Mn0.4Ni0.33V0.1, Fe0.1La0.1Mn0.2Ni0.5V0.1)	
HfNbTaTiZr	LMD	BCC (HfNbTaTiZr)	
MoNbNiTa	L-PBF	BCC (MoNbNiTa)	
MoNbNiTaTi	L-PBF	BCC (MoNbNi0.5TaTi0.5)	
MoNbTaTi	L-PBF	BCC (MoNbTaTi)	
MoNbTaTiZr	L-PBF	BCC (Mo0.6Nb0.6Ta0.6Ti1.4Zr1.4)	
MoNbTaVW	L-PBF	BCC (MoNbTaVW)	
MoNbTaW	L-PBF	BCC (MoNbTaW)	
	LMD	BCC (MoNbTaW)	
		BCC (MoNbTaWx, x = 0, 0.16, 0.33, 0.53)	
		BCC (MoNbTaW, (MoNbTa)xW1-x, (MoTaW)xNb1-x, 0 < x < 0.9)	
		(MoNbTaW)	
MoNbTaWTi	WAAM	BCC (MoNbTaWTi)	
MoNbTiVZr	LMD	BCC + NbTi4-type phase + aZr-rich phase (MoNbTiVZr)	
NbTaTiZr	LMD	BCC (NbTaTiZr)	
		BCC (NbxTa25Ti25Zr50-x, 0 x 50)	

**Table 24:** Ti-6Al-2Zr-1Mo-1V—chemical compositions [1].

Al	Zr	Mo	V	Si	Fe	N	H	O
6.46	1.96	1.12	1.59	0.01	0.03	0.004	0.0012	0.11

**Table 25:** The main optimal parameters used for printing these types of materials [37].

Material	Extrusion Temperature (°C)	Speed (mm/s)	Layer Height (mm)	Infill (%)
PETG	265	20	0.4	100
PETG CF	195	60	0.52	100
PETG KV	265	20	0.35	100

**Table 26:** Heat treatment parameters [37].

Samples Group	Temperature (°C)	Time (min)
1	90	30
2	90	240
3	90	480
4	110	30
5	110	240
6	110	480
7	130	30
8	130	240
9	130	480

**Table 27:** The mechanical characteristics of the materials used in [38].

Material/ Postprocess	Infill Density	Hardness (HRC)	Tensile Strength (MPa)	Impact Strength (J/m^2^)	Flexural Strength (MPa)
Annealed PETG	0.25	69	38	68	51
	0.5	77	41	74	59
	0.75	90	47	88	65
	1	97	52.4	96	70
Annealed CFPETG	0.25	93	53	89	67
	0.5	106	59	101	71
	0.75	119	64	113	78
	1	124	70.3	122	84

**Table 28:** Post-processing treatments used for the parts produced by the additive technologies.

Post-Processing Treatments	Authors	Source	Technology	Material	Post-Processing Parameters	Scope
Annealing	Eduardo da Silva Barbosa Ferreira, Carlos Bruno Barreto Luna, Danilo Diniz Siqueira, Edcleide Maria Araújo, Danyelle Campos de França, Renate Maria Ramos Wellen	[71]	ME	PLA and EVA blends	90 °C, for 5 h in a vacuum oven	Investigation of the effects of EVA blends with PLA and annealing with the aforementioned primers on the increase in crystallinity, modulus of elasticity (tensile strength), resilience to impact bending test, impact strength, HDT, VST, Shore D hardness, and contact angle.
	Javaid Butt, Raghunath Bhaskar	[25]	ME	ABS, PLA, copper-enhanced PLA and aluminum-enhanced ASA (acrylonitrile styrene acrylate)	70, 80, 90 °C for 3D FilaPrint PLA 70, 80, 90 °C for FilaPrint metal copper PLA 105, 115, 125 °C for 3D FilaPrint ABS 70, 80, 90, 105, 115 °C for ASA extra fill aluminum	Effects of annealing at different temperatures on the mechanical properties of two commonly used polymeric materials (ABS and PLA) compared with those of two metal-infused thermoplastics (copper-enhanced PLA and aluminum-enhanced ASA).
	Vukašin Slavković, Nenad Grujović, Aleksandar Dišić, Andreja Radovanović	[29]	ME	Thermoplastics shape memory material	75 °C, 2 h, then cooled down to the ambient temperature at a rate not exceeding 6–30 °C/h.	Identification of the mechanical properties of PLA in terms of the influence of the direction of printing and of post-production annealing on the stiffness and on the tensile and compressive strength.
	Ju Donga, Changtong Meib, Jingquan Hanb, Sunyoung Leec, Qinglin Wua	[36]	ME	PLA and CNF mixture	120 °C for 12 h in a vacuum oven, followed by a 24 h period in which the samples are cooled in an oven without heating	Dynamic mechanical analysis, including temperature ramp, frequency sweep, and creep for annealed samples.
	Danyang Lin, Lianyong Xu, Hongyang Jing, Yongdian Han, Lei Zhao, Yankun Zhang, Huan Li	[2]	L-PBF process	FeCoCrNi high-entropy alloy	Compressed samples (50% reduction) are annealed at 500, 700, 900, or 1100 °C for 2 h	Influence of the annealing process after cold forming on the recrystallization and the mechanical properties of the alloy.
	C. Wanga, X.P. Tana, Z. Dub, S. Chandraa, Z. Suna, C.W.J. Lima, S.B. Tora, C.S. Limc, C.H. Wonga	[63]	DED, L-PBF, SEBM	NiTi Alloys	Solution Heat Treatment (annealing) in a furnace with flowing argon gas at 1000 °C for 6 h, and subsequently quenched in water.	Comparative investigation of the in situ alloy formation of Ni-Ti SMA by DED, L-PBF and SEBM.
	S. Valvez, A.P. Silva, P.N.B Reis, F. Berto	[37]	ME	PETG, CFPETG, and KFPETG	90 °C, 110 °C, and 130 °C and 30 min, 240 min, and 480 min annealing times	Evaluate the behavior of specimens in terms of geometric parameters, hardness, and elastic properties.
	Beth A. Bimber, Reginald F. Hamilton, Jayme Keist, Todd A. Palmer	[38]	ME	PETG and CFPETG	Heat above 5 °C above the glass transition temperature (95 °C) for 60 min, then cool to room temperature.	Study of the effect of ME packing density on the mechanical properties of PETG- and CFPETG-printed samples with different packing densities, such as 25%, 50%, 75%, and 100%, respectively, and with process parameters kept constant at the optimum value.
	Reginald F. Hamilton, Beth A. Bimber, Todd A. Palmer	[73]	LDED	NiTi—SMAs	A solution treatment (annealing) of 950 °C for 24 h followed by aging of the treated material as a solid solution	Deformation analysis at the microstructure scale to investigate the influence of solution and aging heat treatments on the mechanical properties and in particular the superelastic behavior and martensitic transformation morphologies of NiTi alloys produced by additive technology.
	Mubasher Ali, Resy Kumala Sari, Uzair Sajjad, Muhammad Sultan, Hafiz Muhammad Ali	[66]	MJF	Polyamide Plastic (PA-12)	110 °C and 130 °C for a constant time period 2 h.	Study of surface roughness and mechanical strength for seven different lattice polymer structures, namely Diamond, Gyroid, Kelvin, Split-P, SUP, Primitive, and Octet.
	Jeong Min Park, Eun Seong Kim, Hyeonseok Kwon, Praveen Sathiyamoorthi, Kyung Tae Kim, Ji-Hun Yu, Hyoung Seop Kim	[72]	L-PBF	1%C-CoCrFeMnNi High-Entropy Alloy (C-HEA)	800 °C and 900 °C for 10 min, followed by water quenching.	Study of the effect of heat treatment on the evolution of the microstructure and the mechanical properties of a C-HEA material processed by means of L-PBF.
	Jithin J. Marattukalam, Vamsi K. Balla, Mitun Dasc, Srikanth Bontha, Sreeram K. Kalpathy	[59]	LENS^TM^	NiTi Alloy	30 min at 500 °C and 1000 °C in flowing argon, followed by furnace-cooling to room temperature.	Analysis of the effect of heat treatment on the microstructure, phase transition formation, shape memory properties, and corrosion behavior of laser deposited equiatomic NiTi.
	Danyang Lina, Lianyong Xua, Hongyang Jing, Yongdian Han, Lei Zhao, Fumiyoshi Minami	[74]	L-PBF	FeCoCrNi—HEAs	500–1300 °C for 2 h	Evaluation of the impact of different annealing temperatures in the evolution of dislocation network under and the resulting effects on the mechanical properties.
	P. Arjun, V.K. Bidhun, U.K. Lenin, V.P. Amritha, Ribin Varghese Pazhamannil, P. Govindan	[30]	ME	20% carbon-infused PLA	65 °C, 95 °C, 125 °C, and 155 °C considering the glass-transition temperature and melting point for a duration of 30, 60, 120, and 240 min	Study on the impact of process variables and thermal annealing on the tensile strength of carbon fiber and polylactic acid composite thermoplastics.
	Bastian Blinn, Marcus Klein, Christopher Gläßner, Marek Smaga, Jan C. Aurich, Tilmann Beck	[4]	L-PBF, LDW, Continuous Casted	AISI 316L Stainless Steel	1070 °C for 2 h and afterwards cooling in H_2_O	Analysis of the complex relationships between additive manufacturing processes, the resulting microstructure, and the mechanical properties of materials and components.
	O.O. Salman, C. Gammerb, A.K. Chaubey, J. Eckertb, S. Scudino	[6]	L-PBF	AISI 316L Stainless Steel	300 °C, 600 °C, 1000 °C, 1100 °C and 1400 °C for 6 h	Investigation of the influence of annealing at different temperatures on the phase stability, composition, and microstructure of 316L stainless steel produced by L-PBF, in order to understand the corresponding changes in the mechanical properties of specimens under tensile loading.
	Meng Zhang, Chen-Nan Sun, Xiang Zhang, Phoi Chin Goh, Jun Wei, Hua Li, David Hardacre	[75]	L-PBF	Stainless Steel 316L.	Heat treatment in accordance with AMS2750 at two different holding temperatures, i.e., 982 °C and 1093 °C, for 25 min followed by gas quenching to prevent carbide precipitation.	Examination of the monotonic and fatigue properties of L-PBF stainless steels in the as-rolled and heat-treated condition 316L.
	Zhiguang Zhu, Weilin Li, Quy Bau Nguyen, Xianghai An, Wenjun Lu, Zhiming Li, Fern Lan Ng, Sharon Mui Ling Nai, Jun Wei	[10]	L-PBF	304L Stainless Steel	-	Systematic study of the microstructure, mechanical behavior and deformation mechanisms of L-PBF-processed 304L stainless steel.
	Sunil Bhandari, Roberto A. Lopez-Anido, Douglas J. Gardner	[39]	ME	PETG and a semi-crystalline PLA	PETG—120 °C—0, 30, 240, 480 min. PETG-CF—120 °C—0, 30, 240, 480 min PLA—120 °C—0, 30, 240, 480 min PLA-CF—120 °C—0, 30, 240, 480 min PLA—90 °C—30, 240, 480 min PLA-CF—90 °C—30, 240, 480 min	Study to improve the interlayer tensile strength of 3D-printed extrusion-based composites using post-process annealing heat treatment.
	Pushpendra Yadav, Dheeraj Kumar Angajala, Ishant Singhal, Ankit Sahai, Rahul Swarup Sharma	[31]	ME	PLA	80 °C and 120 °C and left in the oven for cooling down	Investigation of the effect of infill density and annealing temperature on mechanical properties of polylactic acid (PLA)-based 3D-printed parts.
	P.D. Nezhadfar, Rakish Shrestha, Nam Phan, Nima Shamsaei	[76]	L-PBF	17-4 PH stainless steel	Procedures based on ASTM A693: H900—482 °C, 1 h, Air cooled H1025 + 552 °C, 4 h, Air cooled CA-H900—1050 °C, 0.5 h, Air cooled CA-H900—482 °C, 1 h, Air cooled CA-H1025—1050 °C, 0.5 h, Air cooled CA-H1025—552 °C, 4 h, Air cooled CA-H1150—1050 °C, 0.5 h, Air cooled CA-H1150—621 °C, 4 h, Air cooled	Synergistic effects, under monotonic tensile and fatigue loading, of annealing heat treatment and surface roughness on the microstructure and mechanical properties of L-PBF precipitation hardening (PH) 17-4 stainless steel (SS).
	P.D. Nezhadfar, Emma Burford, Kathryn Anderson-Wedge, Bin Zhang, Shuai Shao, S.R. Daniewicz, Nima Shamsaei	[77]	L-PBF	17-4 PH stainless steel	CA-H900 includes two steps, first step Condition A (CA), solution heat treatment at 1050 °C for 0.5 h followed by air cooling to room temperature and second step (H900), specimens were held at 482 °C for 1 h followed by air cooling. H1025 condition, only heat treated at 552 °C for 4 h followed by air cooling to room temperature without performing CA step.	Study of the fatigue crack growth (FCG) behavior of precipitation hardening (PH) 17-4 stainless steel (SS) fabricated using the laser powder bed fusion (L-PBF) process and compare it with that of the forged counterpart.
	Sudha Cheruvathur, Eric A. Lass, Carelyn E. Campbell	[78]	L-PBF	17-4 PH stainless steel	The temperatures and times of heat treatment were selected as 650 °C and 1050 °C for 1 h and 1150 °C for 2 h, respectively, to comply with the EOS technical specification, wrought solution heat treatment, condition A, and AMS 5355 homogenizing heat treatment.	Study of the effects of annealing heat treatment and surface roughness on the microstructure and mechanical properties of L-PBF precipitation hardened (PH) 17-4 stainless steel (SS).
	Agnieszka Chmielewska, Bartłomiej Wysocki, Piotr Kwasniak, Mirosław Jakub Kruszewski, Bartosz Michalski, Aleksandra Zielinska, Bogusława Adamczyk-Cieslak, Agnieszka Krawczynska, Joseph Buhagiar, Swįeszkowski	[79]	L-PBF	Ni_55.7_Ti_44.3_ SMAs	Heat treatment 1—1100 °C, 10 h Heat treatment 2—900 °C, 24 h Heat treatment 3—900 °C, 24 h + 1150 °C, 24 h	Analysis of the influence on chemical and phase composition heterogeneity of annealing treatments with different parameters.
	Hardikkumar Prajapati, Divya Chalise, Darshan Ravoori, Robert M. Taylor, Ankur Jain	[42]	ME	ABS	135 °C for 96 h.	Study of the effect of annealing temperature and time on the increase in thermal conductivity, investigated experimentally.
	Heather Simmons, Praphulla Tiwary, James E. Colwell, Marianna Kontopoulou	[80]	Melting and Shaping	ABS	Temperatures between 80 °C and 120 °C.	Investigation of strategies to improve the crystallinity and mechanical properties of PLA without compromising its hydrolytic degradation behavior.
	Sisi Wang, Lode Daelemans, Rudinei Fiorio, Maling Gou, Dagmar R. D’hooge, Karen De Clerckm Ludwig Cardon	[44]	ME	PLA/PHB blend	80 °C and 100 °C for 0.5 h, 1 h, and 2 h.	Analysis of gap-bridging strategies to apply conventional processing optimizations to the 3D printing domain and to specifically enhance the mechanical performance of additive extrusion-based manufacturing of PLA filaments by annealing and/or blending with PHB.
	Kevin R. Hart, Ryan M. Dunn, Jennifer M. Sietins, Clara M. Hofmeister Mock, Michael E. Mackay, Eric D. Wetzel	[51]	ME	ABS	75 °C for 18 h, 125 °C for 2 h, 125 °C for 18 h, 135 °C for 2 h, 135 °C for 18 h, 135 °C for 41 h, 135 °C for 72 h, 135 °C for 168 h, 175 °C for 2 h, 175 °C for 18 h	Investigation of the fracture toughness behavior of polymer samples using additive technologies followed by isothermal annealing heat treatments.
	Radoslaw A. Wach, Piotr Wolszczak, Agnieszka Adamus-Wlodarczyk	[45]	ME	PLA	Temperatures between 65 °C and 95 °C	Study of the possibility of improving the mechanical properties of samples made from PLA using RM technology by increasing the degree of crystallinity of PLA using a post-process thermal annealing treatment.
	Agata Baran, Marek Polanski	[81]	LENS	Ni-Ti SMA	600 °C in a resistance furnace inside a glovebox (MBRAUN LABmaster) with an argon atmosphere (<1 ppm O_2_ and H_2_O)	Investigation of the influence of the deposition rate on the microstructure and phase composition of the Ni-Ti SMA.
	Therese Bormann, Bert Müller, Michael Schinhammer, Anja Kessler, Peter Thalmann, Michael de Wild	[12]	L-PBF	Ni-Ti SMA	800 °C, 0.5 h under argon atmosphere and subsequently water quenched.	Investigation of the effect of laser power, scan speed, and laser trajectory on the microstructure of NiTi SMA parts produced by L-PBF technology and treated by annealing after orientation.
	Vidya Kishore, Xun Chen, Ahmed Arabi Hassen, John Lindahl, Vlastimil Kunc, Chad Duty	[46]	ME	PPS composites	Isothermal 250 °C, 18 h	Study of the influence of post-process isothermal annealing treatment on the mechanical properties of different classes of carbon fiber-reinforced PPS components produced by Big Area Additive Manufacturing (BAAM) printing.
	Nan Yi, Richard Davies, Adam Chaplin, Paul McCutchion, Oana Ghita	[47]	ME	PAEK (PEEK 151, AM 200)	LAT—156 °C, 2 h for PEEK151 and 170 °C, 2 h for AM 200. HAT—200 °C, 4 h for both PAEK grades.	Comparison of crystallization kinetics, morphology, and mechanical properties for parts made from two different PAEK polymers used in material extrusion (ME): fast crystallizing PEEK151 grade, originally designed for injection molding, and slow crystallizing AM 200 grade, specifically designed for ME and post-treated.
	Sheng Li, Hany Hassanin, Moataz M. Attallah, Nicholas J.E. Adkins, Khamis Essa	[19]	L-PBF	Ti_56_Ni_44_ SMA	Heat Treatments 1—950 °C, 3 h and Heat Treatments 2—1000 °C, 2 h, both followed by water quench.	Evaluation of the influence of L-PBF process parameters (laser power, scanning speed, and track spacing) on microstructure development and structural integrity in a Ti-rich TiNi alloy, as well as the influence of a post-process homogenization treatment on microstructure and phase transformations.
	Bo Song, Shujuan Dong, Qi Liu, Hanlin Liao, Christian Coddet	[3]	L-PBF	pure iron powder (product specification MH 300)	Vacuum annealing at 640 °C with a heating rate of 20 °C /min and held for 2.5 h and then cooled within the furnace to the room temperature.	Study of the effects of vacuum annealing heat treatment on the phase structure, microstructure, residual stresses and tensile strength of parts produced by L-PBF technology from pure iron powder.
	Rhugdhrivya Rane, Akhilesh Kulkarni, Hardikkumar Prajapati, Robert Taylor, Ankur Jain, Victoria Chen	[33]	ME	ABS	Temperatures between 120 °C and 180 °C, 3 h	Experimental study of the effect of annealing with simultaneous application of an initial uniaxial load to characterize the increase in tensile strength.
	Amal Nassar, Mona Younis, Mohamed Elzareef, Eman Nassar	[26]	ME	PLA reinforced with 9% carbon fiber—PLACF, ABS reinforced with 9% carbon fiber—ABSCF	50 °C, 120 °C and 150 °C, 10 min	Investigation of the effects of annealing heat treatment on the tensile behavior of 3D-printed high modulus carbon fiber-reinforced materials.
	Marcus Ivey, Garrett W. Melenka, Jason. P. Carey, Cagri Ayranci	[48]	ME	PLACF	85 °C, 115 °C and 145 °C, 2 h	Investigation of the effects of annealing heat treatment on the tensile behavior of 3D-printed high modulus carbon fiber-reinforced materials.
	Jairo Alberto Munoz, Sergio Elizalde, Alexander Komissarov, José María Cabrera	[82]	L-PBF	AlSi11Cu	200 °C, 300 °C, 400 °C, 500 °C, and 550 °C, 1 h, cooling in water (550 °C water temperature).	Study of the effect of different heat treatments on the mechanical, anisotropic and microstructural behavior of a hypoeutectic, near eutectic AlSi11Cu alloy obtained by L-PBF technology.
	John W. Elmer, Karl Fisher, Gordon Gibbs, John Sengthay, Dave Urabe	[67]	WAAM	304L Stainless Steel	Recrystallization at 650 °C, 850 °C and 1050 °C, 0.5 h.	Investigation of how thermomechanical processes, in particular rolling followed by annealing, can improve the mechanical properties and reduce or eliminate anisotropy in the microstructure of 304L stainless steel parts produced by WAAM technology.
	Zhen Zhang, Liqing Wang, Ruize Zhang, Dezheng Yin, Zhanyong Zhao, Peikang Bai, Bin Liu, Fude Wang	[68]	WAAM	Mg_17_Al_12_—AZ91 Mg alloy	Solution annealing (415 °C for 8 h)	Investigation by microstructural analysis and electrochemical measurements of the effect of solution annealing on the microstructure and corrosion behavior of magnesium alloy AZ91 wire and WAAM in 0.1 M NaCl.
	V. Salarvand, H. Sohrabpoor, M.A. Mohammadi, M. Nazari, R. Raghavendra, A. Mostafaei, D. Brabazon	[83]	L-PBF	Austenitic stainless steel 316L	900 °C and 1000 °C	Investigation of the effect of heat treatments with different parameters on the electrochemical corrosion performance of 316L stainless steel parts produced by additive technology compared to untreated parts.
	Yabao Hu, Hanning Chen, Xiaohui Jia, Xiaodan Liang, Jianbo Lei	[8]	L-PBF	Pure Titanium TA1	550 °C, 650 °C and 750 °C, 2 h	Study of the influence of heat treatment applied to titanium components produced by laser beam bed fusion on structural and mechanical properties (tensile strength).
	Ariadna Chueca de Bruijn, Giovanni Gómez-Gras, Marco A. Pérez	[40]	ME	ULTEM^TM^ 9085	180 °C, 1 h, vacuum 190 °C, 2 h 200 °C, 3 h	Study of the influence of thermal annealing above the glass transition temperature as an efficient post-processing technique applied to specimens produced by ME additive technology from ULTEM material and surface finishing of printed parts on mechanical performance.
	Alessandro Carrozza, Alberta Aversa, Paolo Fino, Mariangela Lombardi	[84]	L-PBF	Ti-6Al-2Sn-4Zr-6Mo alloy	HT600—600 °C, 2 h, furnace cooling. HT750—750 °C, 2 h, furnace cooling. HT875—875 °C, 2 h, furnace cooling. HT950—950 °C, 2 h, furnace cooling.	Study of the phenomena occurring in the microstructure of Ti-6Al-2Sn-4Zr-6Mo alloy parts produced by L-PBF additive technology and subjected to heat treatments with different parameters, and how these transformation phenomena affect their mechanical properties.
	Jinghao Xu, Håkan Brodin, Ru Lin Peng, Vladimir Luzin, Johan Moverare	[85]	L-PBF	Nickel-base superalloy CM247LC	Temperatures between 550 °C and 1260 °C, 2 h.	Study of the structural behavior of CM247LC nickel-base superalloy parts produced by L-PBF additive technology and subjected to a wide range of post-processing heat treatments with different parameters.
	Easir Arafat Papon, Anwarul Haque, Scott K. Spear	[41]	ME	PLACF	80 °C, 2 h, slow cooling in vacuum.	Determination of the effects of acid-based fiber oxidation treatment on fiber–matrix interfacial bonding and post-fabrication vacuum annealing heat treatment on the microstructure, fiber bonding and mechanical properties of fiber-reinforced composite parts produced by 3D printing based on ME technology.
	Peng Geng, Ji Zhao, WenzhengWu, Yulei Wang, Bofan Wang, Shuobang Wang, Guiwei Li	[56]	ME	PPS	125 °C, 2 h, 150 °C, 2.25 h, 200 °C, 2.5 h, 225 °C, 2.5 h, dried at 120 °C, 12 h to remove moisture.	Analysis of the effects of varying thermal processing and heat treatment conditions on the accuracy and mechanical properties of 3D ME-printed PPS samples.
	Isaac Ferreira, Carolina Melo, Rui Neto, Margarida Machado, Jorge Lino Alves, Sacha Mould	[43]	ME	Polyamide 12 (PA 12) CF15 short carbon fibers reinforced FX256 unreinforced CF15.	135 °C, 150 °C, 165 °C during 3 h, 6 h, 12 h or 18 h	Evaluate and compare the mechanical performance of ME parts when further post-processed by heat treatment by conducting a study on the influence of annealing on mechanical properties.
	Niranjan Chikkanna, Shankar Krishnapillai, Velmurugan Ramachandran	[57]	ME	PLA	70 °C, 15 min, 37.5 min, 1 h; 90 °C, 15 min, 37.5 min, 1 h; 110 °C, 15 min, 37.5 min, 1 h.	Study of the effect of annealing heat treatment on ME-printed PLA parts under static and dynamic flexural loading conditions on flexural properties.
	Yongjie Zhang, Seung Ki Moon	[35]	ME	ULTEM^TM^ 9085	170 °C, 24 h, 170 °C, 96 h 180 °C, 24 h, 180 °C, 96 h	Analysis of the influence of a proposed new thermal annealing method to improve the mechanical properties of parts produced by ME.
	Ju Dong, Xingyan Huang, Pranjali Muley, Tongyao Wu, Mohamad Barekati-Goudarzi, Zhengjie Tang, Meichun Li, Sunyoung Lee, Dorin Boldor, Qinglin Wu	[49]	ME	PLA composite with CCNFs	25 °C to 250 °C, 0.5 min to 2 h, with heating rate of 5 °C /min.	Demonstrate the effectiveness of microwave heating to anneal 3D-printed PLA composites and increase scientific understanding of using CCNFs as dielectric heat sources to manipulate PLA thermoplastic crystallinity and mechanical properties under microwave irradiation.
	Behnam Akhoundi, Mojtaba Nabipour, Faramarz Hajami, Diana Shakoori	[50]	ME	HTPLA	110 °C, 1 h	Analysis of the effect of nozzle temperature and annealing heat treatment on the mechanical properties of high-temperature PLA parts produced by ME.
	Joaquín Lluch-Cerezo, María Desamparados Meseguer, Juan Antonio García-Manrique, Rut Benavente	[27]	ME	ABS and PLA	100 °C, 118 °C, 135 °C, 153 °C, 170 °C, 205 °C, 240 °C, cooling with the furnace to room temperature for ABS 63 °C, 75 °C, 86 °C, 98 °C, 109 °C, 132 °C, 155 °C, cooling with the furnace to room temperature for PLA	Study of the influence of a ceramic powder mold and post annealing process of two of the most widely used thermoplastic materials in this type of additive technique—ABS and PLA printed by ME on the mechanical properties of parts.
	Musa Yilmaz, Necip Fazil Yilmaz, Mahmut Furkan Kalkan	[58]	ME	PEI/ULTEM^TM^ 1010	220 °C, 225 °C, 230 °C, 235 °C for 3 h	Establishment of heat treatment thresholds to improve interlayer adhesion strength of 3D-printed PEI thermoplastics.
Hot Isostatic Pressing	J.-R. Poulin, A. Kreitcberg, V. Brailovski	[86]	L-PBF	Inconel 625	High temperatures to promoting recrystallization and isostatic compression applied by a pressurized gaseous atmosphere (often argon)	Evaluation of the effect of HIP treatment on the size and distribution of residual defects, microstructure and mechanical properties of IN625 material fabricated by L-PBF with intentionally induced defects.
	Dennise Tanoko Ardi, Lim Guoweia, Niroj Maharjana, Bisma Mutiargoa, Seng Hwee Lengb, Raghavan Srinivasana	[87]	L-PBF	Inconel 718	HIP at 980 °C, 100 MPa and 4 h and shot peening at 0.45 mmA intensity and 200 % coverage.	Comprehensive investigation of the fatigue performance of Inconel 718 parts produced by L-PBF with different post-processing parameters.
	Yutao Zhai, Bo Huang, Xiaodong Mao, Mingjie Zheng	[5]	L-PBF	CLAM	HIP (holding at 1150 °C, 150 MPa for 180 min) and SHT (980 °C for 30 min followed by water cooling, then holding at 740 °C for 90 min and subsequently cooled by air).	Investigation and evaluation of the microstructure and mechanical properties of the L-PBF fabricated CLAM at as-built, standard heat treatment (SHT) and HIP and SHT.
	A. du Plessis, E. Macdonald	[88]	L-PBF	Ti6Al4V	HIP + Annealing heat treatment at 900 °C, 3 h.	Study of the porosity reduction behavior of Ti6Al4V samples made with L-PBF by applying HIP followed by homogenization annealing.
	Hui Wang, Liu Chen, Bogdan Dovgyy, Wenyong Xu, Aixue Sha, Xingwu Li, Huiping Tang, Yong Liu, Hong Wu, Minh-Son Pham	[89]	L-PBF	Hastelloy-X	1176,85 °C for 4 h in vacuum, and then furnace-cooled while HIP was conducted at a hydrostatic pressure of 150 MPa, a temperature of 1176,85 °C for 4 h, then slowly cooled in the furnace at a cooling rate of about 12 °C × min^−1^.	Analysis of literature data to identify optimized printing parameters and evaluation of the strengthening, microstructure and mechanical properties of Hastelloy-X samples produced by laser powder bed fusion and study of the effects of post-processing annealing and HIP on microstructure and mechanical properties.
	P. Ajith Kumar Jain, S. Sattar, D. Mulqueen, D. Pedrazzoli, S.G. Kravchenko, O. G. Kravchenko	[52]	ME	PA 6	0.55 MPa (80 psi) and 200 °C Annealing carried out in a closed oven with ambient air at 150 °C, 170 °C and 200 °C, respectively.	Analysis of the effect of isostatic pressure and annealing heat treatment on improving the mechanical properties of 3D-printed short fiber polymer composites.
	Erica Liverani, Adrian H. A. Lutey, Alessandro Ascari, Alessandro Fortunato	[7]	L-PBF	Austenitic stainless steel 316L	HIP_50_—1150 °C, 50 bar, 3 h HIP_1050_—1150 °C, 1050 bar, 3 h HIP_1500_—1150 °C, 1500 bar, 3 h HIP_2000_—1150 °C, 2000 bar, 3 h.	Investigation of hot isostatic pressing (HIP) in a nitrogen protective environment and solubilizing heat treatment as methods for improving the quality of 316L stainless steel components produced by L-PBF.
	J.M. Alegre, A. Díaz, R. García, L.B. Peral, I.I. Cuesta	[21]	L-PBF	Ti-6Al-4V	Argon environment, pressure of 200 MPa and 850 °C, 2 h and fast-cooling.	Study of the effect of an unconventional HIP cycle on the fatigue behavior of parts made from a Ti-6Al-4V alloy using the L-PBF technique.
	I.S. Grech, J.H. Sullivan, R.J. Lancaster, J. Plummer, N.P. Lavery	[90]	L-PBF	Austenitic stainless steel 316L	1—HIP (T_700_ P_100_)—700 °C, 100 MPa 2—HIP (T_1125_ P_100_)—1125 °C, 100 MPa 3—HIP (T_1200_ P_100_)—1200 °C, 100 MPa 4—HIP (T_700_ P_137_)—700 °C, 137 MPa 5—HIP (T_1125_ P_137_)—1125 °C, 137 MPa 6—HIP (T_1200_ P_137_)—1200 °C, 137 MPa 7—HIP (T_700_ P_200_)—700 °C, 200 MPa 8—HIP (T_1125_ P_200_)—1125 °C, 200 MPa 9—HIP (T_1200_ P_200_)—1200 °C, 200 MPa 10—As-built Not Applicable Not Applicable 11—HIP (T_1125_ P_200_—long hold)—1125 °C, 137 MPa	Comparative analysis of the mechanical and corrosion properties of 316L steel produced by L-PBF and subsequently treated by HIP with various parameters.
	G.E. Bean, T.D. McLouth, D.B. Witkin, S.D. Sitzman, P.M. Adams, R.J. Zaldivar	[24]	L-PBF	Inconel 718	HIP at 1160 °C, 1020 atm, solution treatment at 955 °C, 1 h, water quenching, then precipitation treatment at 720 °C, 8 h followed by furnace cool at 55 °C/h to 620 °C and held for 18 h	Investigation of the variation in tensile properties of specimens made by additive L-PBF technology at different angles to the direction of construction from Inconel 718 material, heat treated and tensile tested at room temperature.
	Nekoda van de Werken, Pratik Koirala, Jafar Ghorbani, Derek Doyle, Mehran Tehrani	[53]	ME	CF-reinforced PEEK composite	200 °C, 250 °C, 300 °C, at 200 psi	Investigation of interlaminar shear behavior, flexural, tensile and compressive mechanical properties when using HIP for post-processing of AM continuous carbon fiber-reinforced PEEK composites.
Ageing	Sabrina Bodziak, Kassim S. Al-Rubaie, Luiz Dalla Valentina, Fernando Humel Lafratta, Edson Costa Santos, André Marcon Zanatta, Yimeng Chen	[15]	L-PBF	maraging 300 steel	510 °C, 2 h and then air-cooled.	Study of the composition and morphology of precipitates in the structure of samples of 300 steel maraged by L-PBF and post-treated by aging at 510 °C for 2 h.
	Soheil Saedi, Ali Sadi Turabi, Mohsen Taheri Andani, Narges Shayesteh Moghaddam, Mohammad Elahinia, Haluk Ersin Karaca	[17]	L-PBF	Ni_50.8_Ti_49.2_ SMA	Temperatures from 350 °C to 600 °C for 30 min, 1 h and 1.5 h.	Study of the effect of laser beam bed fusion (L-PBF) fabrication on the microstructure and texture of Ni-rich NiTi alloy and aging heat treatment parameters (aging time and temperature) on transformation behavior.
	Shuo Yin, Chaoyue Chen, Xingchen Yan, Xiaohua Feng, Richard Jenkins, Peter O’Reilly, Min Liu, Hua Li, Rocco Lupoi	[20]	L-PBF	Maraging 18Ni-300 steel	Various temperatures from 390 °C to 590 °C and time from 1 h to 7 h without pre-solution treatment.	Study of the influence of aging temperature and time on the microstructure, mechanical properties (hardness, strength and ductility), and tribological properties (wear-resistance) of 18Ni-300 maraging L-PBF steel.
	Seong Jun Park, Seong Je Park, Yong Son, Il Hyuk Ahn	[54]	ME	ABS	100 bar and 350 °C, 1 h.	Analysis of the influence of aging heat treatments and application of hot isostatic pressure (WIP) on the mechanical properties and anisotropy of ABS parts produced by material extrusion technology.
	Mohammad Reza Khosravani, Zeljko Bozic, Ali Zolfagharian, Tamara Reinicke	[55]	ME	PLA	Temperature range of −5 °C, 5 h to +35 °C, 5 h.	Analysis of the influence of defects and accelerated thermal aging on the mechanical performance of samples made from PLA material using additive ME technology.
	Lihua Zhang, Wanqing Cao, Yun Zhang, Ripeng Jiang, Xiaoqian Li	[70]	WAAM	Al-Zn-Mg-Li alloy	Solute treatment 470 °C, 5 h and quenching. Two-stage aging at 120 °C, 6 h and 170 °C, 10 h.	Comparative studies to analyze the influence of multidirectional technologies consisting of forging and aging treatments on the microstructure evolution and mechanical properties of Al-Zn-Mg-Li alloy parts using additive technology.
Complex Treatments	Chuncheng Yang, Xiaoyong Tian, Dichen Li, Yi Cao, Feng Zhao, Changquan Shi	[28]	ME	PEEK	Air cooling from 100 °C to 20 °C, 10 min Furnace cooling from 100 °C to 20 °C, 10 min Quenching from 100 °C to 20 °C, 1 min Annealing, heating 20 min from 100 °C to 200 °C, holding 40 min and cooling to 20 °C in 60 min. Tempering, heating 30 min from 20 °C to 250 °C, holding 70 min and cooling to 20 °C in 80 min.	Calculate crystallinity and perform strength tests on samples subjected to different heat treatment conditions in the ME process and compare the results with the crystallinity and mechanical properties (tensile strength, Young’s modulus, and elongation) of the non-heat-treated PEEK material.
	Wei Zhang, Ali Chabok, Bart J. Kooi, Yutao Pei	[1]	L-PBF process	CoCrFeMnNi HEAs systems,	HIP, Annealing, Aging Heat Treatment	Study of the influence of post-treatment on the evolution of the resulting microstructure, relative structure, density, residual stress, grain structure, texture and dislocation networks, element distribution, precipitation, and mechanical properties including hardness, mechanical strength, compressive properties, cryogenic and high temperature properties, fatigue, creep.
	Ali N. Alagha, Shahadat Hussain, Wael Zaki	[91]	L-PBF and DED, LDED additive manufacturing	SMAs—NiTi.	Aging and Solution Annealing	Analysis of the application of additive manufacturing to different SMA systems, with a focus on the influence of process parameters and heat treatment on microstructure, printability and structural changes.
	Guodong Zhang, Neng Li, Jianshi Gao, Huaping Xiong, Huai Yu, Hong Yuan	[62]	Wire-fed EB-DED	Titan alloy—Ti-6Al-2Zr-1Mo-1V	Post-deposition single annealing treatment at 950 ◦C for 2 h, Air in an electrical resistance	Study of the effect of a single post-deposition annealing treatment on microstructure, texture, and tensile property anisotropy.
	Beth A. Bimber, Reginald F. Hamilton, Jayme Keist, Todd A. Palmer	[92]	LDED	NiTi—SMAs	Solutionized (1050 °C, 10 h in an inert environment) and aged (400 °C or 500 °C, for times ranging from 1 to 5 h) before mechanical deformation of austenite	Investigation of the relationship between microstructural anisotropy and over elasticity in large fabricated structures using directed energy deposition additive manufacturing.
	Chola Elangeswaran, Antonio Cutolo, Gokula Krishna Muralidharan, Charlotte de Formanoir, Filippo Berto, Kim Vanmeensel, Brecht Van Hooreweder	[93]	L-PBF	316L steel	Stress relief (SR) heat treatment was carried out at 470 ° C for 5 h in argon atmosphere.	Investigation of the influence of subsequent treatments on the fatigue properties of 316L stainless steel produced by Laser Powder Bed Fusion.
	F.F. Conde, J.D. Escobar, J.P. Oliveira, M. Béreš, A.L. Jardini, W.W. Bose, J.A. Avila	[9]	L-PBF	18 Ni maraging 300 steel	Homogenization cycles were applied between 820 and 980 °C for 1 h, then, two fast cycling steps of 5 min of soaking time were applied at 690 and 720 °C. Finally, aging was conducted at 420 and 480 °C using soaking times of 3 and 6 h.	Study the martensite-to-austenite reversion and its effect on hardness and bending strength after laser beam bed fusion of a maraging 300 steel.
	M S I N Kamariah, W S W Harun, N Z Khalil, F Ahmad, M H Ismail, S Sharif	[94]	L-PBF	316L stainless steel	Different heat treatments of 650 °C, 950 °C, and 1100 °C for 2 h.	Investigation of the effect of heat treatments on the microhardness of compact parts made by L-PBF from 316L stainless steel.
	Bandar AlMangour, Jenn-Ming Yang	[64]	DMLS	17-4 stainless steel	80 °C, argon atmosphere	Study to test induced grain refinement by shot peening (SP) to improve the physical and mechanical properties of 17-4 stainless steel components produced by DMLS.
	Chaolin Tan, Kesong Zhoua, WenyouMa, Panpan Zhang, Min Liu, Tongchun Kuang	[11]	L-PBF	Maraging 300 steels	490 °C, 6 h for age hardening and a solution treatment 840 °C, 1 h, followed by aging at 490 °C for 6 h for comparison. All heat treatments were protected in argon atmosphere and air cooled after holding.	Characterization and analysis of microstructural evolution, nanoprecipitation behavior, and mechanical properties of parts made from maraging 300 steels by L-PBF technology and heat treated.
	Christoph Haberland, Mohammad Elahinia, Jason M Walker, Horst Meier, Jan Frenzel	[13]	L-PBF	Ni-Ti SMA	Annealing: 949.85 °C, 5.5 h, H_2_O aging: 349.85 °C, 24 h, H_2_O	How additive manufacturing and post-process heat-treated parts through annealing and aging affect the structural and functional properties of NiTi SMA parts, and how additive manufacturing can be influenced by optimal setting of process parameters to produce high quality NiTi SMA parts and components.
	James Mutua, Shinya Nakata, Tetsuhiko Onda, Zhong-Chun Chen	[14]	L-PBF	Maraging 300 steels	Solution treatment (annealing) at 820 °C, 1 h and aging at 460 °C, 5 h.	Study of the influence of different process parameters on the densification behavior, surface morphology, microstructure, and mechanical properties of parts produced by L-PBFed from post-heat-treated maraging 300 steel.
	Soheil Saedi, Sayed E. Saghaian, Ahmadreza Jahadakbar, Narges Shayesteh Moghaddam, Mohsen Taheri Andani, Sayed M. Saghaian, Y. Charles Lu, Mohammad Elahinia, Haluk E. Karaca1	[16]	L-PBF	Ni-Ti SMA	Samples placed in argon-filled quartz vials, separated using ceramic barriers and pure titanium to avoid oxidation, were solution annealed at 950 °C for 5.5 h in an oven and then quenched in water. Aging was performed in a vacuum at 350 °C for 15 min, followed by quenching in water.	Analysis of techniques to improve the superelasticity and thermo-mechanical properties of porous NiTi structures produced by additive technology and then heat-treated (solution annealing + aging at 350 °C for 15 min).
	Soheil Saedi, Ali Sadi Turabi, Mohsen Taheri Andani, Christoph Haberland, Haluk Karaca, Mohammad Elahinia	[18]	L-PBF	Ni_50.8_Ti_49.2_ SMA	Annealed at 950 °C, 5.5 h and then water quenched and aging at 350 °C and 450 °C, with time varied from 5 min up to 18 h.	Study of the effects of solution annealing and subsequent aging treatment on the shape memory behavior of Ni-rich Ni50.8Ti49.2 produced by laser beam bed fusion.
	Agnieszka Szust, Grzegorz Adamski	[32]	ME	PETG	Annealed at 60 °C, 1 h and 80 °C, 1 h. Salt remelting at 230 °C, 2 h.	Investigation of the effects of AM process parameters (print orientation and layer height), annealing heat treatment and salt re-melting process on the tensile strength and anisotropy of ME parts.
	Konrad Gruber, Patrycja Szymczyk-Ziołkowska, Szymon Dziuba, Szymon Duda, Paweł Zielonka, Stanislav Seitl, Grzegorz Lesiuk	[95]	L-PBF	Inconel 718	Variant A: Stress relief—1065 °C, 1.5 h; Hot isostatic pressing—n/a; Solutioning—1065 °C, 1 h; Ageing—720 °C, 8 h to 620 °C, 10 h; Argon atmosphere. Variant B: Stress relief—1065 °C, 1.5 h; Hot isostatic pressing—1150 °C, 4 h, 100 MPa; Solutioning—1065 °C, 1 h; Aging—720 °C, 8 h to 620 °C, 10 h; Argon atmosphere.	Study of the fatigue crack growth rate (FCGR) of Inconel 718 parts additively manufactured by laser powder bed fusion (L-PBF) and subjected to post-process heat treatment in two variants: without hot isostatic pressing (variant A) and with hot isostatic pressing (variant B).
	Santiago Aguado-Montero, Carlos Navarro, Jesús Vazquez, Fernando Lasagni, Sebastian Slawik, Jaime Domínguez	[67]	L-PBF (PBF)	TI6AL4V	SP, SP + CASE, LP and annealing 730 °C for 2 h, slow cooling in the furnace, high-vacuum argon atmosphere.	Investigation of the effect of surface treatments such as SP, SP + CASE, and LP on the fatigue behavior of Ti6Al4V specimens produced by additive-additive technology compared to specimens annealed and sandblasted only after AM, used as a reference.
	Shahriar Afkhami, Vahid Javaheri, Edris Dabiri, Heidi Piili, Timo Bjork	[96]	L-PBF	13Cr10Ni 1.7Mo2Al 0.4Mn0.4Si maraging stainless steel	Solution annealing at 850 °C for 30 min, then air cooling to room temperature (20 °C) and aging for 120 min at 525 °C, and, again, air cooling to room temperature	Analysis of the effects of design orientations, heat treatment, and mechanical machining (both as and after machining) on the microstructure, quasi-static mechanical properties, work-hardenability, strain-hardenability, notch toughness, and residual stress of maraging 13Cr10Ni1.7Mo2Al0.4Mn0.4Si stainless steel produced by L-PBF, known under the trade name CX.
	Cecilie V. Funch, Kinga Somlo, Thomas L. Christiansen, Marcel A.J. Somers	[22]	L-PBF	316L stainless steel	HTSN—at 1100 °C, 2 h at 300 mbar partial pressure of nitrogen (N_2_). Conventional austenitization in a high vacuum furnace at 1050 °C, 10 min. Austenitization at a low partial pressure of 45 mbar at 1080 °C, 10 min to avoid N_2_ evaporation.	Analysis of the effects of various post-processing thermochemical treatments on the microstructure and properties of austenitic stainless steel parts produced by additive technology.
	Kristina Navickaitė, Klaus Nestler, Falko Böttger-Hiller, Carmel Matias, Alex Diskin, Oz Golan, Andrey Garkun, Evgeny Strokin, Roman Biletskiy, Daniel Safranchik, Henning Zeidler	[97]	L-PBF	Titanium alloy 08Cr18Ni10Ti,	PEP and powder blasting combined with PEP PEP process, electrolyte temperature should be between 50 °C and 90 °C, while for the EP process temperatures in a range from 0 °C to 55 °C	Comparative analysis of two polishing machining techniques applied to the surfaces of specimens manufactured using the additive technology for titanium alloys.
	Maider Arana, Eneko Ukar, Iker Rodriguez, David Aguilar, Pedro Álvarez	[69]	WAAM	2319 aluminum	Annealing 535 °C, 1.5 h, water quenching and aging 160 °C, 3 h, 6 h, 9 h, 12 h, 15 h, 18 h and 180 °C, 18 h, 20 h, 22 h, 24 h, 26 h	Analysis of the influence of both deposition strategy (geometry, dwell time between passes and torch movement) and heat treatment on parts with different geometries of 2319 aluminum produced by WAAM technology.
	Wolfgang Tillmann, Nelson Filipe Lopes Dias, Dominic Stangier, Christopher Schaak, Simon Höges	[65]	BJP	17–4 PH stainless steel (X2CrNiMo17-12-2 (AISI 316L) and X5CrNiCuNb16-4 (AISI 630),	Precipitation heat treatment at 1020 °C, 0.5 h with quenching at air (solution treatment) and a second step at 480 °C, 3 h followed by air quenching. DLC coating.	Study of the influence of deposition coatings on additively manufactured steel on the hardness and tribological properties of 17-4 PH material parts produced by BJP additive technology and subjected to complex heat treatments.
	X.X. Zhang, A. Lutzb, H. Andrä, M. Lahres, W. Gong, S. Harjo, C. Emmelmann	[98]	L-PBF	AlSi3.5Mg2.5 alloy	Annealing at 380 °C, 1 h Aging at 170 °C, 1 h	Analysis of the influence of the complex post-treatment of annealing followed by aging applied to AlSi3.5Mg2.5 alloy parts produced by additive technology on the mechanical ductility property.
	Changhao Pei, Dong Shi, Huang Yuan, Huaixue Li	[23]	L-PBF	Nickel-base superalloy Inconel 718	Solution treatment (960 °C, 1 h, air cooling) + double aging (720 °C, 8 h, furnace cooling at 55 °C/h to 620 °C, 9.5 h air cooling), and homogenization process (1100 °C, 1.5 h, air cooling)	Investigation of the microstructure, deposition orientation dependence of mechanical properties, and fatigue performance of a nickel based superalloy Inconel 718 produced by L-PBF melting and subsequent heat treatment.
	Alessandro Carrozza, Alberta Aversa, Federico Mazzucato, Emilio Bassini, Diego Manfredi, Sara Biamino, Anna Valente, Paolo Fino	[61]	DED	Ti-6Al-4V alloy	Annealing 1050 °C, 1 h, furnace cooled and aging 540 °C, 4 h Annealing 1050 °C, 1 h, air cooled and aging 540 °C for 4 h Annealing 1050 °C, 1 h, water quenched and aging 540 °C for 4 h	Study of the effect of different multi-stage heat treatments on the microstructure, texture, and mechanical properties of Ti-6Al-4V alloy DED products.
	Jeferson T. Pacheco, Vitor H. Meura, Paulo Rafael A. Bloemer, Marcelo T. Veiga, Osmar C. de Moura Filho, Alexandre Cunha, Moises F. Teixeira	[99]	LDED	316L stainless steel	1040 °C, 2 h, cooling at the room temperature (25 °C) and a cycle of stress relief at 540 °C, 9 h, cooling at the room temperature.	Evaluation of the effect of the deposition direction on the mechanical properties of parts made from AISI 316L material using the L-DED process, in the as-deposited condition and under complex heat treatment conditions.
	Bo Zhang, Wu Wei, Wei Shi, Yanwu Guo, Shengping Wen, Xiaolan Wu, Kunyuan Gao, Li Rong, Hui Huang, Zuoren Nie	[100]	L-PBF	Al-7Si-0.6 Mg alloy	DA at 160 °C, 8 h; SR annealing at 300 °C, 2 h; Solution annealing at 540 °C, 1 h, quenching in cold water at room temperature, then, artificial aging at 160 °C, 8 h.	Study of the behavior of Al-7Si-0.6 Mg alloy parts produced by L-PBF technology and subjected to complex heat treatments, in terms of structural changes and mechanical properties (ductility, tensile, and yield strength).
	Yaozhong Zhang, Aljoscha Roch	[34]	ME	17-4PH stainless steel	1360 °C, 1 h, quenched to room temperature by N_2_ gas flow (30 L/min); 1360 °C, 1 h, quenched to room temperature by N_2_ gas flow (30 L/min) and annealed at 550 °C for 4 h; 1360 °C, 1 h, quenched to room temperature by N2 gas flow (30 L/min) and annealed at 1040 °C, 1 h and annealed at 550 °C, 4 h.	Analysis of the behavior of parts made from 17-4PH stainless steel using ME additive technology and subjected to post-work heat treatments in terms of structural changes and mechanical properties.

**Table 29:** The influence of annealing process on the mechanical properties of PEEK51 3D-printed samples [47].

PEEK51 vertical orientation	as printed	LAT 2 h	LAT 4 h	HAT 2 h	HAT 4 h	Difference,%			
						LAT 2 h	LAT 4 h	HAT 2 h	HAT 4 h
Elastic modulus, MPa	24,000	32,000	34,000	25,000	28,000	33.33	41.67	4.17	16.67
Strength, MPa	26	24	25	10	18	−7.69	−3.85	−61.54	−30.77
Elongation at break, %	2.70	2.20	2.50	0.80	1.50	−18.52	−7.41	−70.37	−44.44
**PEEK51** **flat orientation**	**as printed**	**LAT** **2 h**	**LAT 4 h**	**HAT 2 h**	**HAT 4 h**	**Difference,%**			
						**LAT 2 h**	**LAT 4 h**	**HAT 2 h**	**HAT 4 h**
Elastic modulus, MPa	2800	3000	3300	3100	3200	7.14	17.86	10.71	14.29
Strength, MPa	60	76	72	68	68	26.67	20.00	13.33	13.33
Elongation at break, %	9	13	12	8	13	44.44	33.33	−11.11	44.44
**PEEK51** **side orientation**	**as printed**	**LAT** **2 h**	**LAT 4 h**	**HAT 2 h**	**HAT 4 h**	**Difference,%**			
						**LAT 2 h**	**LAT 4 h**	**HAT 2 h**	**HAT 4 h**
Elastic modulus, MPa	25,000	29,000	33,000	27,000	28,000	16.00	32.00	8.00	12.00
Strength, MPa	50.00	48.00	46.00	60.00	46.00	−4.00	−8.00	20.00	−8.00
Elongation at break, %	8.00	5.00	4.00	8.00	4.00	−37.50	−50.00	0	−50.00

**Table 30:** The influence of annealing process on the mechanical properties of AM200 3D-printed samples [47].

AM200 vertical orientation	as printed	LAT 2 h	LAT 4 h	HAT 2 h	HAT 4 h	Difference,%			
						LAT 2 h	LAT 4 h	HAT 2 h	HAT 4 h
Elastic modulus, MPa	2200	2700	2600	3100	2800	22.73	18.18	40.91	27.27
Strength, MPa	40	42	44	38	39	5.00	10.00	−5.00	−2.50
Elongation at break, %	5.7	5.2	5	3.8	4.5	−8.77	−12.28	−33.3	−21.05
**AM 200** **flat orientation**	**as printed**	**LAT** **2 h**	**LAT 4 h**	**HAT 2 h**	**HAT 4 h**	**Difference,%**			
						**LAT 2 h**	**LAT 4 h**	**HAT 2 h**	**HAT 4 h**
Elastic modulus, MPa	2700	3300	3200	2900	2800	22.22	18.52	7.41	3.70
Strength, MPa	50	64	63	58	56	28.00	26.00	16.00	12.00
Elongation at break, %	23	15	14	12	9	−34.78	−39.13	−47.82	−60.87
**AM 200** **side orientation**	**as printed**	**LAT** **2 h**	**LAT 4 h**	**HAT 2 h**	**HAT 4 h**	**Difference,%**			
						**LAT 2 h**	**LAT 4 h**	**HAT 2 h**	**HAT 4 h**
Elastic modulus, MPa	2400	2600	2500	3500	3300	8.33	4.17	45.83	37.50
Strength, MPa	58	70	72	76	66	20.69	24.14	31.03	13.79
Elongation at break, %	17	16	17.5	18	14	−5.88	2.94	5.88	−17.65

**Table 31:** The effects of annealing on mechanical properties of 3D-printed parts [39].

Material	Annealing Temperature	Interlayer Tensile Strength	Interlayer Young’s Modulus	Interlayer Strain to Failure
PLA	90 °C	Minor increase	No change	Major increase
PLA-CF	90 °C	Major increase	Major increase	Major increase
PLA	120 °C	No change	No change	Minor increase
PLA-CF		No change	No change	No change
PETG		No change	No change	Minor increase
PETG-CF		Major increase	Major increase	Major increase

## Data Availability

Data are contained within the article.

## Abbreviations

AM	Additive Manufacturing
RM	Rapid Manufacturing
CAD	Computer-Aided Design
SS	Stainless Steel
FCG	Fatigue Crack Growth
PH	Precipitation Hardening
PLA	Polylactic Acid
PHB	Poly(3-HydroxyButyrate)
EVA	Ethylene Vinyl Acetate
ABS	Acrylonitrile Butadiene Styrene
ME	Material extrusion
ASA	Acrilonitril Stiren Acrilat
PEEK	Poly-Ether-Ether-Ketone
CNF	Cellulose Nanofibers
ME	Material extrusion
HEAs	High Entropy Alloys
HIP	Hot Isostatic Pressing
DED	Direct Energy Deposition
LDED	Laser Direct Energy Deposition
SMAs	Shape Memory Alloys
EB-DED	Electron Beam Directed Energy Deposition
L-PBF	Laser beam bed fusion
SEBM	Selective Electron Beam Melting
PETG	PolyEthylene Terephthalate Glycol
CFPETG	Carbon Fibers PETG reinforced
KFPETG	Kevlar Fibers (Aramid Fibers) PETG reinforced
MJF	Multi Jet Fusion
HEA	High Entropy Alloy
C-HEA	Carbon-High Entropy Alloy
LENS^TM^	Laser Engineered Net Shaping
L-PBF	Laser Powder Bed Fusion
LDW	Laser Deposition Welding
CLAM	China Low Activation Martensitic steel
SHT	Standard Heat Treatment
DMLS	Direct Metal Laser Sintering
PPS	PolyPhenylene Sulfide
PA 6	Polyamide 6 (Nylon 6)
PEEK151	Poly Ether Ketone
PAEK	Poly Acryl Ether Ketone
LAT	Low Annealing Temperature
HAT	High Annealing Temperature
WAAM	Wire-Arc Additive Manufacturing
CASE^®^	Chemical Assisted Surface Enhancement
SP	Shot Peening
LP	Laser Peening
HTSN	High Temperature Solution Nitriding
PEP	Plasma Electrolytic Polishing
EP	Electrochemical Polishing
BJP	Binder Jet Printed
DLC	Diamond-Like Carbon
DA	Direct Aging
SR	Stress Relief
CCNFs	Carbonized Cellulose Nanofibers
PEI	PolyEtherImide

## References

[1] Zhang W., Chabok A., Kooi B.J., Pei Y. (2022). Additive Manufactured High Entropy Alloys: A Review of the Microstructure and Properties. Mater. Des..

[2] Lin D., Xu L., Jing H., Han Y., Zhao L., Zhang Y., Li H. (2020). A Strong, Ductile, High-Entropy FeCoCrNi Alloy with Fine Grains Fabricated via Additive Manufacturing and a Single Cold Deformation and Annealing Cycle. Addit. Manuf..

[3] Song B., Dong S., Liu Q., Liao H., Coddet C. (2014). Vacuum Heat Treatment of Iron Parts Produced by Selective Laser Melting: Microstructure, Residual Stress and Tensile Behavior. Mater. Des..

[4] Aviles D.A. (2018). Metals An Investigation of the Microstructure and Fatigue Behavior of Additively Manufactured AISI 316L Stainless Steel with Regard to the Influence of Heat Treatment. Metals.

[5] Zhai Y., Huang B., Mao X., Zheng M. (2019). Effect of Hot Isostatic Pressing on Microstructure and Mechanical Properties of CLAM Steel Produced by Selective Laser Melting. J. Nucl. Mater..

[6] Salman O.O., Gammer C., Chaubey A.K., Eckert J., Scudino S. (2019). Effect of Heat Treatment on Microstructure and Mechanical Properties of 316L Steel Synthesized by Selective Laser Melting. Mater. Sci. Eng. A.

[7] Liverani E., Lutey A., Ascari A., Fortunato A. (2020). The Effects of Hot Isostatic Pressing (HIP) and Solubilization Heat Treatment on the Density, Mechanical Properties, and Microstructure of Austenitic Stainless Steel Parts Produced by Selective Laser Melting (SLM). Int. J. Adv. Manuf. Technol..

[8] Hu Y., Chen H., Jia X., Liang X., Lei J. (2022). Heat Treatment of Titanium Manufactured by Selective Laser Melting: Microstructure and Tensile Properties. J. Mater. Res. Technol..

[9] Conde F.F., Escobar J.D., Oliveira J.P., Béreš M., Jardini A.L., Bose W.W., Avila J.A. (2019). Effect of Thermal Cycling and Aging Stages on the Microstructure and Bending Strength of a Selective Laser Melted 300-Grade Maraging Steel. Mater. Sci. Eng. A.

[10] Zhu Z., Li W., Nguyen Q.B., An X., Lu W., Li Z., Ng F.L., Ling Nai S.M., Wei J. (2020). Enhanced Strength–Ductility Synergy and Transformation-Induced Plasticity of the Selective Laser Melting Fabricated 304L Stainless Steel. Addit. Manuf..

[11] Tan C., Zhou K., Ma W., Zhang P., Liu M., Kuang T. (2017). Microstructural Evolution, Nanoprecipitation Behavior and Mechanical Properties of Selective Laser Melted High-Performance Grade 300 Maraging Steel. Mater. Des..

[12] Bormann T., Müller B., Schinhammer M., Kessler A., Thalmann P., de Wild M. (2014). Microstructure of Selective Laser Melted Nickel–Titanium. Mater. Charact..

[13] Haberland C., Elahinia M., Walker J., Meier H., Frenzel J. (2014). On the Development of High Quality NiTi Shape Memory and Pseudoelastic Parts by Additive Manufacturing. Smart Mater. Struct..

[14] Mutua J., Nakata S., Onda T., Chen Z.-C. (2018). Optimization of Selective Laser Melting Parameters and Influence of Post Heat Treatment on Microstructure and Mechanical Properties of Maraging Steel. Mater. Des..

[15] Bodziak S., Al-Rubaie K.S., Valentina L.D., Lafratta F.H., Santos E.C., Zanatta A.M., Chen Y. (2019). Precipitation in 300 Grade Maraging Steel Built by Selective Laser Melting: Aging at 510 °C for 2 h. Mater. Charact..

[16] Saedi S., Saghaian S.E., Jahadakbar A., Shayesteh Moghaddam N., Taheri Andani M., Saghaian S.M., Lu Y.C., Elahinia M., Karaca H.E. (2018). Shape Memory Response of Porous NiTi Shape Memory Alloys Fabricated by Selective Laser Melting. J. Mater. Sci. Mater. Med..

[17] Saedi S., Turabi A.S., Andani M.T., Moghaddam N.S., Elahinia M., Karaca H.E. (2017). Texture, Aging, and Superelasticity of Selective Laser Melting Fabricated Ni-Rich NiTi Alloys. Mater. Sci. Eng. A.

[18] The Influence of Heat Treatment on the Thermomechanical Response of Ni-Rich NiTi Alloys Manufactured by Selective Laser Melting-ScienceDirect. https://www.sciencedirect.com/science/article/pii/S0925838816307459.

[19] Li S., Hassanin H., Attallah M.M., Adkins N.J.E., Essa K. (2016). The Development of TiNi-Based Negative Poisson’s Ratio Structure Using Selective Laser Melting. Acta Mater..

[20] Yin S., Chen C., Yan X., Feng X., Jenkins R., O’Reilly P., Liu M., Li H., Lupoi R. (2018). The Influence of Aging Temperature and Aging Time on the Mechanical and Tribological Properties of Selective Laser Melted Maraging 18Ni-300 Steel. Addit. Manuf..

[21] Effect of HIP Post-Processing at 850 °C/200 MPa in the Fatigue Behavior of Ti-6Al-4V Alloy Fabricated by Selective Laser Melting. https://www.x-mol.net/paper/article/1541103637099290624.

[22] Funch C.V., Somlo K., Christiansen T.L., Somers M.A.J. (2022). Thermochemical Post-Processing of Additively Manufactured Austenitic Stainless Steel. Surf. Coat. Technol..

[23] Pei C., Shi D., Yuan H., Li H. (2019). Assessment of Mechanical Properties and Fatigue Performance of a Selective Laser Melted Nickel-Base Superalloy Inconel 718. Mater. Sci. Eng. A.

[24] Bean G.E., McLouth T.D., Witkin D.B., Sitzman S.D., Adams P.M., Zaldivar R.J. (2019). Build Orientation Effects on Texture and Mechanical Properties of Selective Laser Melting Inconel 718. J. Mater. Eng. Perform..

[25] Butt J., Bhaskar R. (2020). Investigating the Effects of Annealing on the Mechanical Properties of FFF-Printed Thermoplastics. JMMP.

[26] Nassar A., Younis M., Elzareef M., Nassar E. (2021). Effects of Heat-Treatment on Tensile Behavior and Dimension Stability of 3D Printed Carbon Fiber Reinforced Composites. Polymers.

[27] Influence of Thermal Annealing Temperatures on Powder Mould Effectiveness to Avoid Deformations in ABS and PLA 3D-Printed Parts-PubMed. https://pubmed.ncbi.nlm.nih.gov/35808650/.

[28] Yang C., Tian X., Li D., Cao Y., Zhao F., Shi C. (2017). Influence of Thermal Processing Conditions in 3D Printing on the Crystallinity and Mechanical Properties of PEEK Material. J. Mater. Process. Technol..

[29] Slavković V., Grujović N., Disic A., Radovanović A. Influence of Annealing and Printing Directions on Mechanical Properties of PLA Shape Memory Polymer Produced by Fused Deposition Modeling. Proceedings of the 6th International Congress of Serbian Society of Mechanics Mountain Tara.

[30] Arjun P., Bidhun V.K., Lenin U.K., Amritha V.P., Pazhamannil R.V., Govindan P. (2022). Effects of Process Parameters and Annealing on the Tensile Strength of 3D Printed Carbon Fiber Reinforced Polylactic Acid. Mater. Today Proc..

[31] Yadav P., Angajala D.K., Singhal I., Sahai A., Sharma R.S. (2021). Evaluating Mechanical Strength of Three Dimensional Printed PLA Parts by Free Form Fabrication. Mater. Today Proc..

[32] Szust A., Adamski G. (2022). Using Thermal Annealing and Salt Remelting to Increase Tensile Properties of 3D FDM Prints. Eng. Fail. Anal..

[33] Rane R., Kulkarni A., Prajapati H., Taylor R., Jain A., Chen V. (2020). Post-Process Effects of Isothermal Annealing and Initially Applied Static Uniaxial Loading on the Ultimate Tensile Strength of Fused Filament Fabrication Parts. Materials.

[34] Zhang Y., Roch A. (2022). Fused Filament Fabrication and Sintering of 17-4PH Stainless Steel. Manuf. Lett..

[35] The Effect of Annealing on Additive Manufactured ULTEMTM 9085 Mechanical Properties-PMC. https://www.ncbi.nlm.nih.gov/pmc/articles/PMC8199117/.

[36] Dong J., Mei C., Han J., Lee S., Wu Q. (2019). 3D Printed Poly(Lactic Acid) Composites with Grafted Cellulose Nanofibers: Effect of Nanofiber and Post-Fabrication Annealing Treatment on Composite Flexural Properties. Addit. Manuf..

[37] Valvez S., Silva A.P., Reis P.N.B., Berto F. (2022). Annealing Effect on Mechanical Properties of 3D Printed Composites. Procedia Struct. Integr..

[38] Augmenting Effect of Infill Density and Annealing on Mechanical Properties of PETG and CFPETG Composites Fabricated by FDM-ScienceDirect. https://www.sciencedirect.com/science/article/pii/S2214785320376458.

[39] Bhandari S., Lopez-Anido R.A., Gardner D.J. (2019). Enhancing the Interlayer Tensile Strength of 3D Printed Short Carbon Fiber Reinforced PETG and PLA Composites via Annealing. Addit. Manuf..

[40] de Bruijn A.C., Gómez-Gras G., Pérez M.A. (2022). Thermal Annealing as a Post-Process for Additively Manufactured Ultem 9085 Parts. Procedia Comput. Sci..

[41] Papon E.A., Haque A., Spear S.K. (2020). Effects of Functionalization and Annealing in Enhancing the Interfacial Bonding and Mechanical Properties of 3D Printed Fiber-Reinforced Composites. Mater. Today Commun..

[42] Prajapati H., Chalise D., Ravoori D., Taylor R.M., Jain A. (2019). Improvement in Build-Direction Thermal Conductivity in Extrusion-Based Polymer Additive Manufacturing through Thermal Annealing. Addit. Manuf..

[43] Ferreira I., Melo C., Neto R., Machado M., Alves J., Mould S. (2020). Study of the Annealing Influence on the Mechanical Performance of PA12 and PA12 Fibre Reinforced FFF Printed Specimens. Rapid Prototyp. J..

[44] Wang S., Daelemans L., Fiorio R., Gou M., D’hooge D.R., De Clerck K., Cardon L. (2019). Improving Mechanical Properties for Extrusion-Based Additive Manufacturing of Poly(Lactic Acid) by Annealing and Blending with Poly(3-Hydroxybutyrate). Polymers.

[45] Wach R.A., Wolszczak P., Adamus-Wlodarczyk A. (2018). Enhancement of Mechanical Properties of FDM-PLA Parts via Thermal Annealing. Macromol. Mater. Eng..

[46] Kishore V., Chen X., Hassen A.A., Lindahl J., Kunc V., Duty C. (2020). Post-Process Annealing of Large-Scale 3D Printed Polyphenylene Sulfide Composites. Addit. Manuf..

[47] Yi N., Davies R., Chaplin A., McCutchion P., Ghita O. (2021). Slow and Fast Crystallising Poly Aryl Ether Ketones (PAEKs) in 3D Printing: Crystallisation Kinetics, Morphology, and Mechanical Properties. Addit. Manuf..

[48] Ivey M., Melenka G., Carey J., Ayranci C. (2016). Characterizing Short-Fiber-Reinforced Composites Produced Using Additive Manufacturing. Adv. Manuf. Polym. Compos. Sci..

[49] Dong J., Huang X., Muley P., Wu T., Barekati-Goudarzi M., Tang Z., Li M., Lee S., Boldor D., Wu Q. (2020). Carbonized Cellulose Nanofibers as Dielectric Heat Sources for Microwave Annealing 3D Printed PLA Composite. Compos. Part B Eng..

[50] An Experimental Study of Nozzle Temperature and Heat Treatment (Annealing) Effects on Mechanical Properties of High-Temperature Polylactic Acid in Fused Deposition Modeling-Akhoundi-2020-Polymer Engineering & Science-Wiley Online Library. https://4spepublications.onlinelibrary.wiley.com/doi/full/10.1002/pen.25353.

[51] Hart K.R., Dunn R.M., Sietins J.M., Hofmeister Mock C.M., Mackay M.E., Wetzel E.D. (2018). Increased Fracture Toughness of Additively Manufactured Amorphous Thermoplastics via Thermal Annealing. Polymer.

[52] Kumar Jain P.A., Sattar S., Mulqueen D., Pedrazzoli D., Kravchenko S.G., Kravchenko O.G. (2022). Role of Annealing and Isostatic Compaction on Mechanical Properties of 3D Printed Short Glass Fiber Nylon Composites. Addit. Manuf..

[53] van de Werken N., Koirala P., Ghorbani J., Doyle D., Tehrani M. (2021). Investigating the Hot Isostatic Pressing of an Additively Manufactured Continuous Carbon Fiber Reinforced PEEK Composite. Addit. Manuf..

[54] Park S.J., Park S.J., Son Y., Ahn I.H. (2022). Reducing Anisotropy of a Part Fabricated by Material Extrusion via Warm Isostatic Pressure (WIP) Process. Addit. Manuf..

[55] Khosravani M., Božić Ž., Zolfagharian A., Reinicke T. (2022). Failure Analysis of 3D-Printed PLA Components: Impact of Manufacturing Defects and Thermal Ageing. Eng. Fail. Anal..

[56] Geng P., Zhao j., Wu W., Wang Y., Wang B., Wang S., Li G. (2018). Effect of Thermal Processing and Heat Treatment Condition on 3D Printing PPS Properties. Polymers.

[57] Chikkanna N., Krishnapillai S., Ramachandran V. (2022). Static and Dynamic Flexural Behaviour of Printed Polylactic Acid with Thermal Annealing: Parametric Optimisation and Empirical Modelling. Int. J. Adv. Manuf. Technol..

[58] Rheology, Crystallinity, and Mechanical Investigation of Interlayer Adhesion Strength by Thermal Annealing of Polyetherimide (PEI/ULTEM 1010) Parts Produced by 3D Printing|SpringerLink. https://link.springer.com/article/10.1007/s11665-022-07049-z.

[59] Marattukalam J.J., Balla V.K., Das M., Bontha S., Kalpathy S.K. (2018). Effect of Heat Treatment on Microstructure, Corrosion, and Shape Memory Characteristics of Laser Deposited NiTi Alloy. J. Alloy. Compd..

[60] https://www.Manufacturingguide.Com/En/Laser-Engineered-Net-Shaping-Lens-0.

[61] Carrozza A., Aversa A., Mazzucato F., Bassini E., Manfredi D., Biamino S., Valente A., Fino P. (2022). An Investigation on the Effect of Different Multi-Step Heat Treatments on the Microstructure, Texture and Mechanical Properties of the DED-Produced Ti-6Al-4V Alloy. Mater. Charact..

[62] Zhang G., Li N., Gao J., Xiong H., Yu H., Yuan H. (2022). Wire-Fed Electron Beam Directed Energy Deposition of Ti–6Al–2Zr–1Mo–1V Alloy and the Effect of Annealing on the Microstructure, Texture, and Anisotropy of Tensile Properties. Addit. Manuf..

[63] Wang C., Tan X.P., Du Z., Chandra S., Sun Z., Lim C.W.J., Tor S.B., Lim C.S., Wong C.H. (2019). Additive Manufacturing of NiTi Shape Memory Alloys Using Pre-Mixed Powders. J. Mater. Process. Technol..

[64] AlMangour B., Yang J.-M. (2016). Improving the Surface Quality and Mechanical Properties by Shot-Peening of 17-4 Stainless Steel Fabricated by Additive Manufacturing. Mater. Des..

[65] Tillmann W., Lopes Dias N.F., Stangier D., Schaak C., Höges S. (2022). Heat Treatment of Binder Jet Printed 17–4 PH Stainless Steel for Subsequent Deposition of Tribo-Functional Diamond-like Carbon Coatings. Mater. Des..

[66] Ali M., Sari R., Sajjad U., Sultan M., Ali H. (2021). Effect of Annealing on Microstructures and Mechanical Properties of PA-12 Lattice Structures Proceeded by Multi Jet Fusion Technology. Addit. Manuf..

[67] Elmer J.W., Fisher K., Gibbs G., Sengthay J., Urabe D. (2022). Post-Build Thermomechanical Processing of Wire Arc Additively Manufactured Stainless Steel for Improved Mechanical Properties and Reduction of Crystallographic Texture. Addit. Manuf..

[68] Zhang Z., Wang L., Zhang R., Yin D., Zhao Z., Bai P., Liu B., Wang F. (2022). Effect of Solution Annealing on Microstructures and Corrosion Behavior of Wire and Arc Additive Manufactured AZ91 Magnesium Alloy in Sodium Chloride Solution. J. Mater. Res. Technol..

[69] Arana M., Ukar E., Rodriguez I., Aguilar D., Álvarez P. (2022). Influence of Deposition Strategy and Heat Treatment on Mechanical Properties and Microstructure of 2319 Aluminium WAAM Components. Mater. Des..

[70] Zhang L., Cao W., Zhang Y., Jiang R., Li X. (2022). Microstructure Evolution and Enhanced Mechanical Properties of Additive Manufacturing Al-Zn-Mg-Li Alloy via Forging and Aging Treatment. J. Mater. Res. Technol..

[71] da Silva Barbosa Ferreira E., Luna C.B.B., Siqueira D.D., Araújo E.M., de França D.C., Wellen R.M.R. (2022). Annealing Effect on Pla/Eva Blends Performance. J. Polym. Environ..

[72] Park J.M., Kim E.S., Kwon H., Sathiyamoorthi P., Kim K.T., Yu J.-H., Kim H.S. (2021). Effect of Heat Treatment on Microstructural Heterogeneity and Mechanical Properties of 1%C-CoCrFeMnNi Alloy Fabricated by Selective Laser Melting. Addit. Manuf..

[73] Hamilton R.F., Bimber B.A., Palmer T.A. (2018). Correlating Microstructure and Superelasticity of Directed Energy Deposition Additive Manufactured Ni-Rich NiTi Alloys. J. Alloys Compd..

[74] Lin D., Xu L., Jing H., Han Y., Zhao L., Minami F. (2020). Effects of Annealing on the Structure and Mechanical Properties of FeCoCrNi High-Entropy Alloy Fabricated via Selective Laser Melting. Addit. Manuf..

[75] Zhang M., Sun C.-N., Zhang X., Goh P., Wei J., Li H., Hardacre D. (2017). Elucidating the Relations Between Monotonic and Fatigue Properties of Laser Powder Bed Fusion Stainless Steel 316L. JOM.

[76] Nezhadfar P.D., Shrestha R., Phan N., Shamsaei N. (2019). Fatigue Behavior of Additively Manufactured 17-4 PH Stainless Steel: Synergistic Effects of Surface Roughness and Heat Treatment. Int. J. Fatigue.

[77] Nezhadfar P.D., Burford E., Anderson-Wedge K., Zhang B., Shao S., Daniewicz S.R., Shamsaei N. (2019). Fatigue Crack Growth Behavior of Additively Manufactured 17-4 PH Stainless Steel: Effects of Build Orientation and Microstructure. Int. J. Fatigue.

[78] Cheruvathur S., Lass E.A., Campbell C.E. (2016). Additive Manufacturing of 17-4 PH Stainless Steel: Post-Processing Heat Treatment to Achieve Uniform Reproducible Microstructure. JOM.

[79] Chmielewska A., Wysocki B., Kwaśniak P., Kruszewski M.J., Michalski B., Zielińska A., Adamczyk-Cieślak B., Krawczyńska A., Buhagiar J., Święszkowski W. (2022). Heat Treatment of NiTi Alloys Fabricated Using Laser Powder Bed Fusion (LPBF) from Elementally Blended Powders. Materials.

[80] Simmons H., Tiwary P., Colwell J.E., Kontopoulou M. (2019). Improvements in the Crystallinity and Mechanical Properties of PLA by Nucleation and Annealing. Polym. Degrad. Stab..

[81] Baran A., Polanski M. (2018). Microstructure and Properties of LENS (Laser Engineered Net Shaping) Manufactured Ni-Ti Shape Memory Alloy. J. Alloys Compd..

[82] Muñoz J.A., Elizalde S., Komissarov A., Cabrera J.M. (2022). Effect of Heat Treatments on the Mechanical and Microstructural Behavior of a Hypoeutectic Al Alloy Obtained by Laser Powder Bed Fusion. Mater. Sci. Eng. A.

[83] Salarvand V., Sohrabpoor H., Mohammadi M.A., Nazari M., Raghavendra R., Mostafaei A., Brabazon D. (2022). Microstructure and Corrosion Evaluation of As-Built and Heat-Treated 316L Stainless Steel Manufactured by Laser Powder Bed Fusion. J. Mater. Res. Technol..

[84] Carrozza A., Aversa A., Fino P., Lombardi M. (2022). Towards Customized Heat Treatments and Mechanical Properties in the LPBF-Processed Ti-6Al-2Sn-4Zr-6Mo Alloy. Mater. Des..

[85] Xu J., Brodin H., Peng R.L., Luzin V., Moverare J. (2022). Effect of Heat Treatment Temperature on the Microstructural Evolution of CM247LC Superalloy by Laser Powder Bed Fusion. Mater. Charact..

[86] Poulin J.-R., Kreitcberg A., Brailovski V. (2021). Effect of Hot Isostatic Pressing of Laser Powder Bed Fused Inconel 625 with Purposely Induced Defects on the Residual Porosity and Fatigue Crack Propagation Behavior. Addit. Manuf..

[87] Ardi D.T., Guowei L., Maharjan N., Mutiargo B., Leng S.H., Srinivasan R. (2020). Effects of Post-Processing Route on Fatigue Performance of Laser Powder Bed Fusion Inconel 718. Addit. Manuf..

[88] du Plessis A., Macdonald E. (2020). Hot Isostatic Pressing in Metal Additive Manufacturing: X-Ray Tomography Reveals Details of Pore Closure. Addit. Manuf..

[89] Wang H., Chen L., Dovgyy B., Xu W., Sha A., Li X., Tang H., Liu Y., Wu H., Pham M.-S. (2021). Micro-Cracking, Microstructure and Mechanical Properties of Hastelloy-X Alloy Printed by Laser Powder Bed Fusion: As-Built, Annealed and Hot-Isostatic Pressed. Addit. Manuf..

[90] Grech I., Sullivan J., Lancaster R., Plummer J., Lavery N. (2022). The Optimisation of Hot Isostatic Pressing Treatments for Enhanced Mechanical and Corrosion Performance of Stainless Steel 316L Produced by Laser Powder Bed Fusion. Addit. Manuf..

[91] Alagha A.N., Hussain S., Zaki W. (2021). Additive Manufacturing of Shape Memory Alloys: A Review with Emphasis on Powder Bed Systems. Mater. Des..

[92] Bimber B.A., Hamilton R.F., Keist J., Palmer T.A. (2016). Anisotropic Microstructure and Superelasticity of Additive Manufactured NiTi Alloy Bulk Builds Using Laser Directed Energy Deposition. Mater. Sci. Eng. A.

[93] Elangeswaran C., Cutolo A., Muralidharan G.K., de Formanoir C., Berto F., Vanmeensel K., Van Hooreweder B. (2019). Effect of Post-Treatments on the Fatigue Behaviour of 316L Stainless Steel Manufactured by Laser Powder Bed Fusion. Int. J. Fatigue.

[94] Kamariah M.S.I.N., Harun W.S.W., Khalil N.Z., Ahmad F., Ismail M.H., Sharif S. (2017). Effect of Heat Treatment on Mechanical Properties and Microstructure of Selective Laser Melting 316L Stainless Steel. IOP Conf. Ser. Mater. Sci. Eng..

[95] Gruber K., Szymczyk-Ziółkowska P., Dziuba S., Duda S., Zielonka P., Seitl S., Lesiuk G. (2023). Fatigue Crack Growth Characterization of Inconel 718 after Additive Manufacturing by Laser Powder Bed Fusion and Heat Treatment. Int. J. Fatigue.

[96] Afkhami S., Javaheri V., Dabiri E., Piili H., Björk T. (2021). Effects of Manufacturing Parameters, Heat Treatment, and Machining on the Physical and Mechanical Properties of 13Cr10Ni1.7Mo2Al0.4Mn0.4Si Steel Processed by Laser Powder Bed Fusion. Mater. Sci. Eng. A.

[97] Navickaitė K., Nestler K., Böttger-Hiller F., Matias C., Diskin A., Golan O., Garkun A., Strokin E., Biletskiy R., Safranchik D. (2022). Efficient Polishing of Additive Manufactured Titanium Alloys. Procedia CIRP.

[98] Zhang X.X., Lutz A., Andrä H., Lahres M., Gong W., Harjo S., Emmelmann C. (2022). Strain Hardening Behavior of Additively Manufactured and Annealed AlSi3.5Mg2.5 Alloy. J. Alloys Compd..

[99] Pacheco J.T., Meura V.H., Bloemer P.R.A., Veiga M.T., de Moura Filho O.C., Cunha A., Teixeira M.F. (2022). Laser Directed Energy Deposition of AISI 316L Stainless Steel: The Effect of Build Direction on Mechanical Properties in as-Built and Heat-Treated Conditions. Adv. Ind. Manuf. Eng..

[100] Zhang S., Zhang Y., Qi J., Zou Z., Qian Y. (2023). Effect of Heat Treatment on the Microstructure and Mechanical Properties of Additive Manufactured Ti-6.5Al-2Zr-1Mo-1V Alloy. Materials.

[101] Aguado-Montero S., Navarro C., Vázquez J., Lasagni F., Slawik S., Domínguez J. (2022). Fatigue Behaviour of PBF Additive Manufactured TI6AL4V Alloy after Shot and Laser Peening. Int. J. Fatigue.

[102] Zhang B., Wei W., Shi W., Guo Y., Wen S., Wu X., Gao K., Rong L., Huang H., Nie Z. (2022). Effect of Heat Treatment on the Microstructure and Mechanical Properties of Er-Containing Al–7Si–0.6 Mg Alloy by Laser Powder Bed Fusion. J. Mater. Res. Technol..

